# Recovery of RNA‐dependent RNA polymerase 6 gene‐knockout phenotypes in *Nicotiana benthamiana* via *in vivo* generation of inverted repeat construct of the trans‐acting short interference RNA3 sequence

**DOI:** 10.1111/tpj.70350

**Published:** 2025-07-18

**Authors:** Kouki Matsuo

**Affiliations:** ^1^ Biomanufacturing Process Research Center (BPRC) National Institute of Advanced Industrial Science and Technology (AIST) 2‐17‐2‐1 Tsukisamu‐Higashi, Toyohira‐ku Sapporo 062‐8517 Japan

**Keywords:** auxin response factor, miR390, *Nicotiana benthamiana*, *RNA‐dependent RNA polymerase 6*, *TAS3*, trans‐acting short interference RNA, transient expression

## Abstract

Pharmaceutical recombinant proteins are widely produced using plants; however, several challenges such as low productivity limit their application. To overcome these problems, *RNA‐dependent RNA polymerase 6*‐knockout *Nicotiana benthamiana* plants (*rdr6* plants) were previously produced for the mass production of recombinant proteins. These *rdr6* plants produced higher amounts of recombinant proteins than wild‐type plants, but their practical use for recombinant protein production is limited by their sterility and small leaf size. As an essential component of the microRNA 390‐trans‐acting short interference RNA3‐auxin response factor (miR390‐*TAS3*‐*ARF*) pathway, *rdr6* modulates leaf morphology, flower architecture, and embryo development. In fact, the expression levels of *ARF*3 and 4 genes differed in leaves, flowers, and buds of wild‐type and *rdr6* plants. Therefore, a dysfunctional miR390‐*TAS3*‐*ARF* pathway might be responsible for the sterility and small leaf size of the *rdr6* plants. Herein, to overcome the disadvantages of *rdr6* plants while maintaining the high productivity of recombinant proteins, the miR390‐*TAS3*‐*ARF* pathway was reconstructed. Transgenic *rdr6* plants expressing the inverted repeat constructs of the *TAS3* sequence of *N. benthamiana* were successfully produced (TAS3i plants). These plants produced seeds and large leaves, like the wild‐type plants, while maintaining a high productivity of recombinant proteins, like the *rdr6* plants. Thus, the TAS3i plants can be considered useful for producing recombinant proteins. This is the first report that the miR390‐*TAS3*‐*ARF* pathway can function in plants in the absence of *RDR6*.

## Significance Statement

*RNA‐dependent RNA polymerase 6*‐knockout *Nicotiana benthamiana* (*rdr6* plants) produce recombinant proteins in large amounts but suffer from sterility and small leaf phenotypes. Therefore, we reconstructed the miR390‐*TAS3*‐*ARF* pathway, generating TAS3i plants. These plants retained high recombinant protein production levels of *rdr6* plants and wild‐type‐like seed production and large leaf phenotypes. This study is the first to demonstrate that the miR390‐*TAS3*‐*ARF* pathway functions without *RDR6*, making TAS3i plants valuable for recombinant protein production.

## INTRODUCTION

The production of recombinant pharmaceutical proteins in plants, including antibodies, cytokines, and vaccine components, is a safe and cost‐effective approach. For example, recombinant human glucocerebrosidase for Gaucher disease was produced in genetically modified carrot cells and approved by the US Food and Drug Administration (Drake et al., 2017). ZMapp, a drug comprising three anti‐Ebola virus monoclonal antibodies produced in *N. benthamiana* plants, was used to treat patients infected with the Ebola virus in 2014 (Lyon et al., 2014). Recently, plant‐based COVID‐19 vaccines containing virus‐like particles of the SARS‐CoV‐2 spike (S) protein were produced in *N. benthamiana* and approved for use in Canada (Su et al., 2023). Based on these successes, many plant‐based pharmaceuticals are in clinical trials (Sethi et al., 2021).

Studies have attempted to improve the productivity of recombinant proteins in plants (Desai et al., 2010) using several approaches, such as repressing the RNA‐silencing mechanism. siRNAs targeting transiently expressed transgenes in agroinfiltrated leaves were shown to be produced within 2 days postinfiltration (dpi) (Bhaskar et al., 2009; Kościańska et al., 2005). Our previous studies showed that the fluorescence of green fluorescent protein (GFP) in agroinfiltrated leaves of wild‐type (WT) *N. benthamiana* plants without co‐expression of RNA‐silencing suppressor proteins gradually decreased after 3 dpi because of degradation of GFP mRNA (Matsuo, 2022; Matsuo & Atsumi, 2019; Matsuo & Matsumura, 2017). Furthermore, viral gene‐silencing suppressor proteins have been used to improve the production of transiently expressed recombinant proteins (Garabagi et al., 2012; Lombardi et al., 2009). Previously, we created RNA‐silencing‐related gene‐repressed or knockout *N. benthamiana* plants, such as *RNA‐dependent RNA polymerase 6*‐knockout (*rdr6*) and *Dicer‐like 2* and *4* (*DCL 2* and *4*) double‐knockout (*dcl2dcl4*) plants (Matsuo, 2022; Matsuo & Atsumi, 2019; Matsuo & Matsumura, 2017). The productivity of recombinant proteins in these plants was significantly higher than in WT plants. However, the *rdr6* and *dcl2dcl4* plants were smaller than the WT plants, with relatively larger axillary buds and elongated leaves. Furthermore, the flowers of the *rdr6* plants were sterile and had torn petals. These features make the mass production of recombinant proteins from these plants challenging.

*RDR6* participates in the synthesis of double‐stranded RNAs (dsRNAs) that are subsequently processed into different types of small interfering RNAs (siRNAs), including trans‐acting siRNAs (ta‐siRNAs) (Olmedo‐Monfil et al., 2010; Peragine et al., 2004; Willmann et al., 2011). Reportedly, *RDR6* interacts with *SGS3* and other RNAs, causing them to form siRNA bodies in the cytoplasm (Tan et al., 2023). *SGS3* also plays an important role in the synthesis of ta‐siRNA (Adenot et al., 2006). Consequently, *RDR6* is essential for the development of leaves and juveniles, lateral root production, and post‐transcriptional gene silencing. The ta‐siRNAs are produced from noncoding transcripts that can regulate coding genes via mRNA cleavage *in trans* (Deng et al., 2018). MicroRNA390 (miR390), a noncoding RNA, targets the trans‐acting short interference RNA3 (*TAS3*) transcripts to control the auxin response factor (*ARF*) genes. The miR390‐*TAS3*‐*ARF* pathway is a typical example of ta‐siRNAs. In this pathway, miR390 preferentially binds to *Argonaute 7* (*AGO7*) to specifically cleave *TAS3* (Montgomery et al., 2008). The resultant *TAS3* products are transcribed by *RDR6* into dsRNAs and further cleaved by *DCL4* into 21 nt‐long siRNAs, known as *TAS3* ta‐siRNAs, which then target and regulate several *ARF*s, including *ARF2*, *ARF3*, and *ARF4* (Adenot et al., 2006; Gasciolli et al., 2005; Kou et al., 2022; Liu et al., 2024; Su et al., 2017; Williams et al., 2005; Xia et al., 2017; Yamamuro et al., 2016; Yoshikawa et al., 2005). These *ARF*s modulate the signaling pathway for auxin, an endogenous plant hormone that controls most plant developmental processes (Cancé et al., 2022). Furthermore, *ARF*s *2–4* were shown to be essential for plant growth and development, especially for female and male gametophyte development in Arabidopsis (Liu et al., 2018; Liu et al., 2024). The miR390‐*TAS3*‐*ARF* pathway mainly modulates leaf morphology, developmental transitions, flower and root architecture, embryo development, responses to biotic and abiotic stresses, and phytohormone crosstalk (Deng et al., 2018; Fahlgren et al., 2006; Li et al., 2024). In *rdr6* plants, the *TAS3* ta‐siRNAs are dysfunctional as the *TAS3* locus‐derived double‐stranded RNA synthesis is impaired by *RDR6*, resulting in various morphological changes compared to WT plants (Matsui et al., 2014).

**Figure 1:**
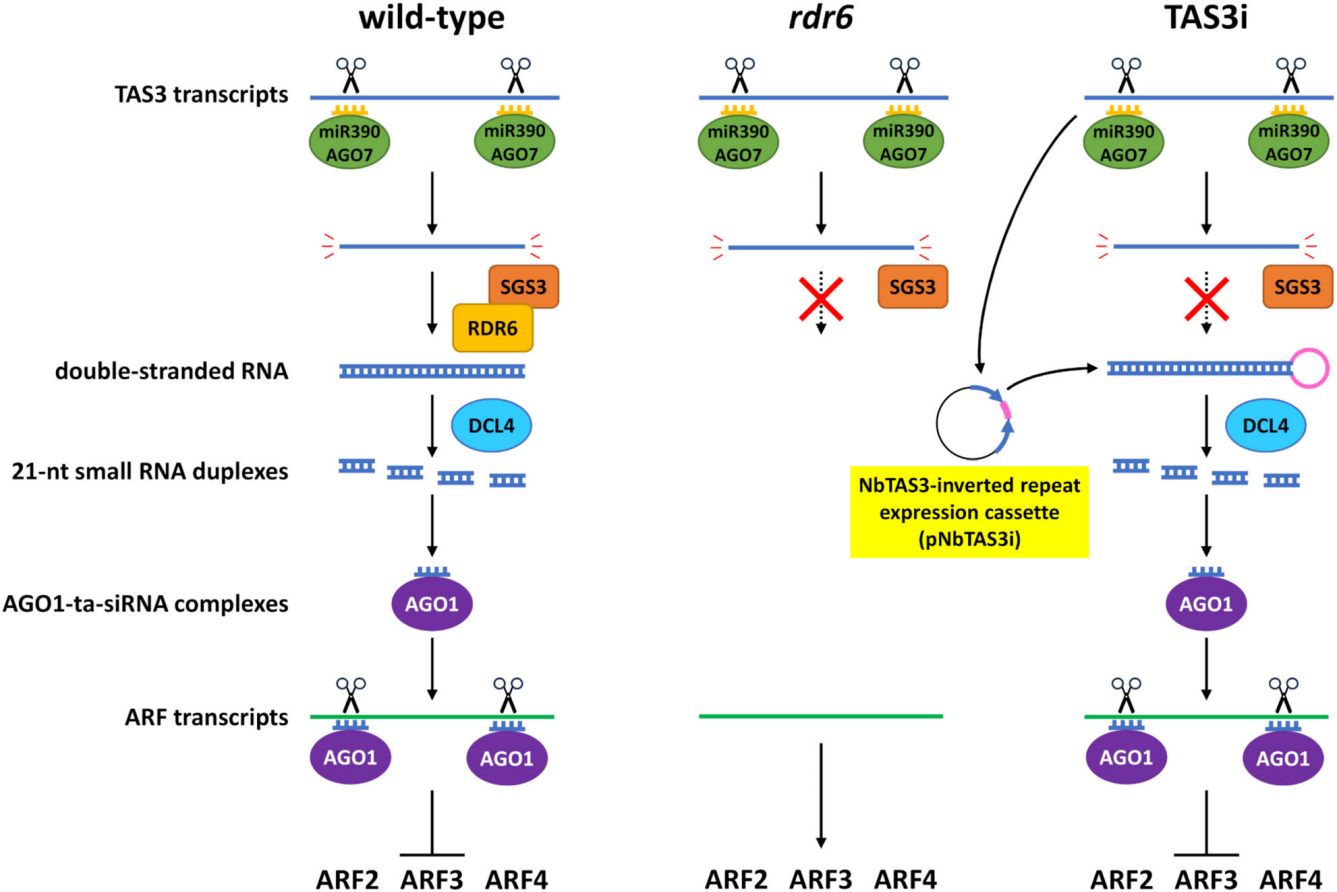
Schematic view of the miR390‐*TAS3*‐*ARF* pathway in the wild‐type, *rdr6*, and TAS3i plants. In the *rdr6* plants, the function of *RDR6* is lost, impairing the negative regulation of the *ARF* genes. In the TAS3i plants, the function of *RDR6* is lost; the double‐stranded RNA of the *NbTAS3* sequence is supplied from the *NbTAS3* inverted repeat expression cassette. Thus, the negative regulation of *ARF* genes could be recovered.

As mentioned above, despite their high productivity, the application of *rdr6* plants for recombinant protein production is limited by their sterility and small size. In this study, to overcome these disadvantages while maintaining their high productivity, attempts were made to improve the *rdr6* plants, especially in recovering their fertility. First, a comparison of the gene expression status of *rdr6* and WT plants using RNA‐seq analysis revealed that the expression levels of auxin‐activated signaling pathway‐related genes were increased in *rdr6* plants, while those related to pollen and anther formation were decreased. This indicated that *RDR6* is involved in auxin‐activated signaling pathways and the expression of reproductivity‐related genes in plants, probably through the miR390‐*TAS3*‐*ARF* pathway. To restore the fertility due to the loss of rdr6 function, transgenic *rdr6* plants expressing double‐stranded RNA derived from *Nicotiana benthamiana TAS3* were produced (TAS3i plants) to complement the function of *RDR6* in the miR390‐*TAS3*‐*ARF* pathway (Figure 1). The phenotypes of the TAS3i plants were restored to those of WT plants, including fertility. This is the first to show the genetic restoration of a defective miR390‐*TAS3*‐*ARF* pathway in plants.

## RESULTS

### RNA‐seq analysis of *rdr6* plants

To investigate the overall gene expression changes between WT and the *rdr6* plants, RNA‐seq analysis was performed. In this study, the *N. tabacum* reference genome Ntab‐TN90 (NCBI Reference sequence: GCF_000715135.1) was used for differentially expressed genes (DEGsgene (DEG) and gene ontology (GO)) analyses as a reliable *N. benthamiana* database was unavailable at that time (Kurotani et al., 2025). The leaves, flowers, and buds from the WT and *rdr6* plants were used for RNA‐seq analysis (Tables 1, S1 and Figure S1). Fisher's exact test results showed that 14 and 8 genes were up‐ and downregulated in the leaves of the *rdr6* plants compared to the WT plants, respectively (Table 1; Table S1 and Figure S1). The expression level of *RDR6* in the *rdr6* plants was significantly reduced compared to that of WT plants, indicating that it was inactivated in these plants (Table 1). DEG and GO analyses of the leaves revealed that the auxin‐activated signaling pathway‐related genes were upregulated in the *rdr6* plants compared to those in WT plants (Tables 1 and 2; Table S4), suggesting that the miR390‐*TAS3*‐*ARF* pathway might be dysfunctional. Interestingly, the endopeptidase inhibitor‐related genes were also upregulated in the leaves of the *rdr6* plants (Tables S10 and S4). Genes with variable expression levels in the *rdr6* plants were predominantly seen in the flowers and buds; namely, 24 and 51 genes in flowers and 104 and 67 genes in buds were up‐ and downregulated, respectively (Tables S1 and Figure S1). This observation revealed that *rdr6* might be closely associated with the reproductive organs in plants. GO analysis showed that the genes determining the floral meristem were upregulated in the flowers from the *rdr6* plants, which might be responsible for the abnormal flower morphology in these plants (Table S6). Conversely, anther‐ and spermidine biosynthesis‐related genes were downregulated in the buds of the *rdr6* plants (Table S9). As polyamines, including spermidine, are essential for pollen formation and fertilization (Aloisi et al., 2016, 2022), this downregulation might affect the sterility of the *rdr6* plants. As shown in Table 2, the expression levels of *ARF4* in the *rdr6* plants were upregulated in all tested tissues compared to WT plants (Tables 1 and 2; Tables S2 and S3). The expression levels of *ARF3* were also upregulated in the *rdr6* flowers, but no significant differences were observed between these levels in the leaves and buds (Table 2). No differences were observed in the expression levels of *ARF2* between WT and the *rdr6* plants in all tested tissues (Table 2).

**Table 1:** The differentially expressed genes in leaves (wild‐type vs. *rdr6*).

Gene_ID	Gene	Gbkey	Expression amounts in the leaves of		m.value [log2(expression ratio)]	Products
			Wild‐type plants	*rdr6* plants		
rna‐XM_016584313.1	LOC107765621	mRNA	2	60	5.018	Probable glutathione S‐transferase
rna‐XM_016638433.1	LOC107813195	mRNA	16	286	4.271	Uncharacterized LOC107813195, transcript variant X2
rna‐XM_016638425.1	LOC107813195	mRNA	16	286	4.271	Uncharacterized LOC107813195, transcript variant X1
rna‐XM_016642073.1	LOC107816366	mRNA	50	227	2.294	Cysteine protease inhibitor 8‐like
rna‐XM_016653169.1	LOC107826218	mRNA	236	813	1.895	**Auxin response factor 4‐like**
rna‐XM_016606813.1	LOC107785490	mRNA	293	1003	1.886	**Auxin response factor 4‐like**
gene‐LOC107796867	LOC107796867	exon	94	316	1.860	BURP domain protein RD22‐like
gene‐LOC107778472	LOC107778472	exon	79	256	1.807	Linoleate 13S‐lipoxygenase 2–1, chloroplastic‐like
rna‐XM_016655118.1	LOC107827891	mRNA	141	392	1.586	Proteinase inhibitor I‐B‐like
rna‐XM_016578159.1	LOC107760143	mRNA	90	245	1.556	Probable glutathione S‐transferase parA
rna‐XM_016602129.1	LOC107781430	mRNA	286	659	1.315	Wound‐induced proteinase inhibitor 2‐like
rna‐XM_016618457.1	LOC107795773	mRNA	205	447	1.236	Uncharacterized LOC107795773
rna‐XM_016618800.1	LOC107796079	mRNA	731	1301	0.943	UDP‐glycosyltransferase 74E2‐like
rna‐XM_016605601.1	LOC107784467	mRNA	1156	2049	0.937	Uncharacterized LOC107784467
rna‐XM_016582029.1	LOC107763541	mRNA	12 053	6369	−0.809	S‐adenosylmethionine synthase 2‐like
rna‐XM_016597252.1	LOC107777255	mRNA	1357	615	−1.031	Sulfate transporter 4.1, chloroplastic‐like
rna‐XM_016626077.1	LOC107802564	mRNA	792	272	−1.431	Ribonuclease Y‐like
rna‐XM_016623777.1	LOC107800585	mRNA	1076	334	−1.577	Isoflavone reductase homolog
gene‐LOC107798614	LOC107798614	exon	1369	423	−1.583	Isoflavone reductase homolog
rna‐XM_016599844.1	LOC107779408	mRNA	666	180	−1.776	Putative glutathione‐specific gamma‐glutamylcyclotransferase 2
rna‐XM_016610965.1	LOC107789186	mRNA	1043	266	−1.860	Putative glutathione‐specific gamma‐glutamylcyclotransferase 2
rna‐XM_016621745.1	LOC107798716	mRNA	287	71	−1.904	RNA‐dependent RNA polymerase 6

**Table 2:** The auxin response factor sequences used for RNA‐seq analysis and the derived *m*. values [log2(expression ratio)] of differentially expressed ARFs.

Product	Gene_ID	Gbkey	*m*. values [log2(expression ratio)]								
			Wild‐type versus *rdr6* in			Wild‐type versus TAS3i in			*rdr6* versus TAS3i in		
			Leaves	Flowers	Buds	Leaves	Flowers	Buds	Leaves	Flowers	Buds
Auxin response factor 2	rna‐XM_016582006.1	mRNA	—	—	—	—	—	—	—	—	—
Auxin response factor 2‐like	rna‐XM_016628009.1	mRNA	—	—	—	—	—	—	—	—	—
Auxin response factor 2‐like	rna‐XM_016621326.1	mRNA	—	—	—	—	—	—	—	—	—
*Auxin response factor 2‐like* ^a^	*rna‐XM_016586158.1*	*mRNA*	—	—	—	—	—	—	—	—	—
Auxin response factor 2‐like, transcript variant X1	rna‐XM_016659409.1	mRNA	—	—	—	—	—	—	—	—	—
Auxin response factor 2‐like, transcript variant X1	rna‐XM_016611230.1	mRNA	—	—	—	—	—	—	—	—	—
Auxin response factor 2‐like, transcript variant X2	rna‐XM_016659410.1	mRNA	—	—	—	—	—	—	—	—	—
Auxin response factor 2‐like, transcript variant X2	rna‐XM_016611231.1	mRNA	—	—	—	—	—	—	—	—	—
Auxin response factor 2‐like, transcript variant X3	rna‐XM_016659411.1	mRNA	—	—	—	—	—	—	—	—	—
Auxin response factor 2‐like, transcript variant X3	rna‐XM_016611232.1	mRNA	—	—	—	—	—	—	—	—	—
Auxin response factor 3‐like, transcript variant X1	rna‐XR_001652067.1	misc_RNA	—	1.117	—	−2.093	—	−1.733	−2.600	−2.142	−2.061
Auxin response factor 3‐like, transcript variant X1	rna‐XR_001647814.1	misc_RNA	—	1.246	—	−1.994	—	−1.795	−2.586	−2.256	−2.026
Auxin response factor 3‐like, transcript variant X2	rna‐XM_016627510.1	mRNA	—	1.118	—	−2.080	—	−1.733	−2.588	−2.140	−2.061
Auxin response factor 3‐like, transcript variant X2	rna‐XM_016606410.1	mRNA	—	1.243	—	−1.994	—	−1.795	−2.586	−2.256	−2.026
Auxin response factor 3‐like, transcript variant X3	rna‐XR_001652068.1	misc_RNA	—	1.117	—	−2.092	—	−1.733	−2.600	−2.142	−2.061
Auxin response factor 3‐like, transcript variant X3	rna‐XR_001647815.1	misc_RNA	—	1.246	—	−1.994	—	−1.795	−2.586	−2.256	−2.026
Auxin response factor 4‐like	rna‐XM_016653169.1	mRNA	1.895	2.176	1.402	−2.338	—	−2.322	−4.243	−3.290	−3.731
Auxin response factor 4‐like	rna‐XM_016606813.1	mRNA	1.886	1.903	1.448	−2.541	—	−2.607	−4.437	−3.071	−3.522

^a^ No matched sequences in *N. benthamiana*.

### Production of TAS3i plants

**Figure 2:**
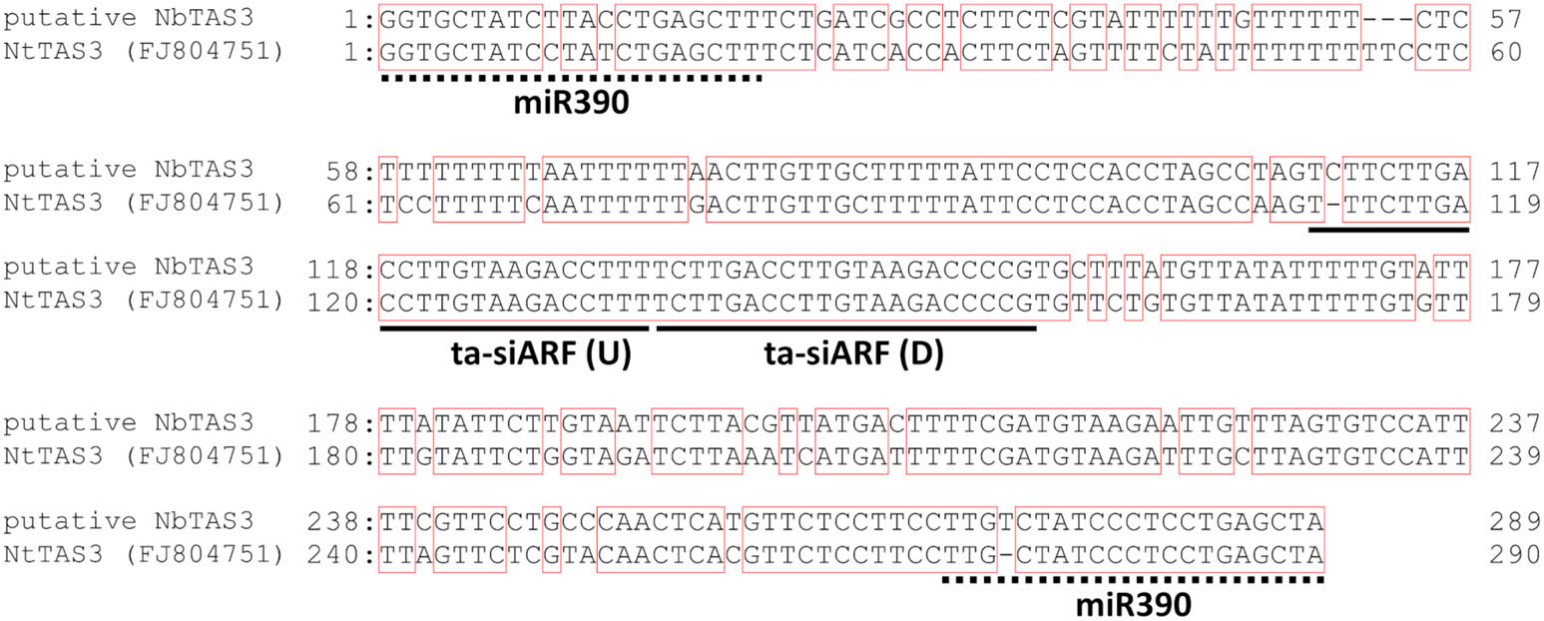
Alignment of the Nt*TAS3* and putative Nb*TAS3* sequences. Nucleotide sequences of ta‐siARF loci from *Nicotiana benthamiana* (Niben101Scf07184Ctg012:211877‐212 317). The cloned Nb*TAS3* and previously reported *TAS3* sequences for *N. tabacum* (NCBI accession number FJ804751) were aligned and compared. The putative miR390 and ta‐si*ARF* sequences, ta‐*siARF* (U, upstream) and ta‐si*ARF* (D, downstream), are indicated by the dotted and black lines, respectively.

To regulate the expression levels of *ARF* genes by reconstituting the miR390‐*TAS3*‐*ARF* pathway in the *rdr6* plants, transgenic *rdr6* plants expressing the inverted repeat of the *TAS3* sequence were produced. The *TAS3* sequence of *N. benthamiana*, *NbTAS3* was cloned using a primer pair (*NbTAS3*‐F and *NbTAS3*‐R) based on the previously reported *TAS3*‐like sequence (Krasnikova et al., 2009). Interestingly, sequencing analysis revealed that the cloned *NbTAS3* sequence was very similar to that of *N. tabacum* but different from that of *N. benthamiana* (Figure 2). The *NbTAS3* sequence used in this study was obtained from Niben101Scf07184g02002.1:211877–212 317 (as a reverse strand, 441 bp). As expected, the *NbTAS3* sequence had two putative miR390 and two ta‐siARF sequences (Figure 2; Figure S2). To produce the TAS3i plants, an inverted repeat construct of *NbTAS3* was introduced into the *rdr6* plants by *Agrobacterium*‐mediated transformation. The T_1_ transgenic TAS3i plants with the inverted repeat construct were used for the subsequent experiments.

**Figure 3:**
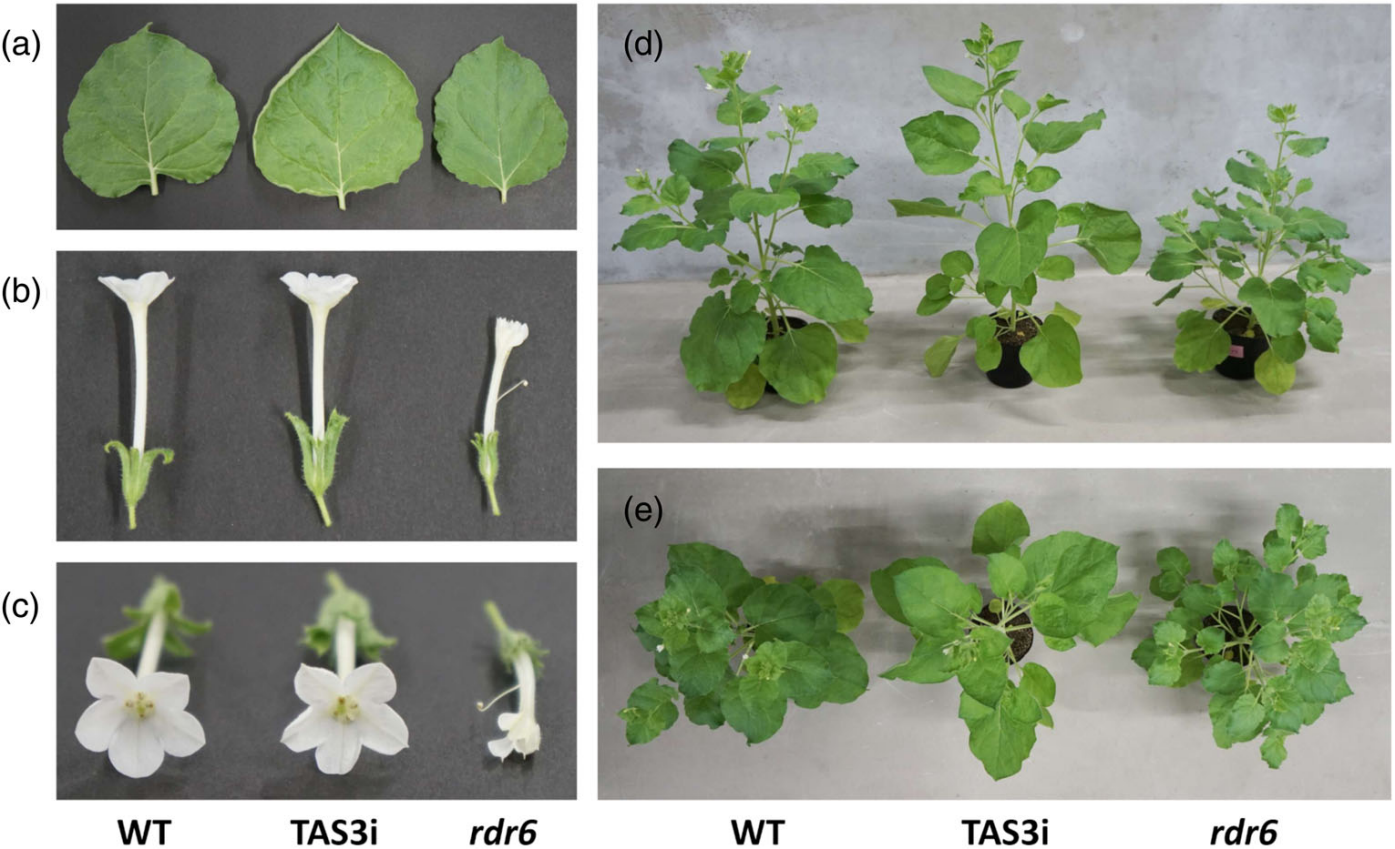
The phenotypes of the TAS3i and *rdr6* plants at 58 days after seeding. Leaves (a) and flowers (b and c) were collected from the respective plants. The whole plant bodies were photographed from the side (d) and above (e).

Unlike the *rdr6* plants, the TAS3i plants were as large as the WT plants, and their leaves had slightly curled edges (Figure 3a). Furthermore, the shapes of the TAS3i flowers were almost similar to those of the WT plants, except for the slightly pointed petal tips compared to WT flowers. These flowers could produce seeds, unlike *rdr6* plants (Figure 3b,c). The sizes of the body and leaves of the TAS3i plants were similar to those of the WT plants (Figure 3d,e).

The numbers of flowers and ovaries (before enlargement, with ovules, and without ovules) of the WT, TAS3i, and *rdr6* plants were counted at 91 days after seeding (Figures S3 and S4). The average numbers of total flowers of the WT, TAS3i, and *rdr6* plants were 72.8, 71,8, and 128.3, respectively (Figure S3a). These results show that the *rdr6* knockout increased the number of flowers, but the expression of the inverted Nb*TAS3* repeat restored this number to the same level as the WT plants. Similarly, the average numbers of flowers in bloom, ovaries before enlargement, ovaries with ovules, and ovaries without ovules in TAS3i plants were similar to those of the WT plants (Figure S3b–e). These findings indicate that the ovaries of the TAS3i plants might take longer to enlarge than the WT plants because the TAS3i plants have more ovaries before enlargement than the WT plants. Consequently, the seed‐forming ability was recovered in the TAS3i plants. The germination rate of the seeds from the TAS3i plants was 98% (49/50).

### RNA‐seq analysis of TAS3i plants

Similar to the *rdr6* plants, leaves, flowers, and buds collected from the TAS3i plants were subjected to RNA‐seq analysis, revealing variable gene expression levels between the WT and TAS3i plants (Tables S11–S20). In the leaves of the TAS3i plants, 42 and 12 genes were up‐ and downregulated compared to the WT plants, respectively (Table S1 and Figure S1). In the TAS3i plants, 10 and 53 genes in the flowers and 61 and 126 genes in the buds were up‐ and downregulated, respectively (Table S1 and Figure S1). The number of upregulated genes in the flowers and buds of the *rdr6* plants was significantly lower than those of the WT plants. Furthermore, compared to WT plants, the number of downregulated genes in the buds was higher in the TAS3i plants than in the *rdr6* plants. These results indicate that the gene expression profile in the TAS3i plants was altered by the restoration of the miR390‐*TAS3*‐*ARF* pathway. The expression level of *RDR6* in the TAS3i leaves was significantly lower than that of the WT leaves, similar to the *rdr6* plants. In the TAS3i leaves, several defense‐related genes were upregulated compared to WT plants, similar to the *rdr6* plants (Tables S11, S12, S15, and S16). Interestingly, contrary to the case in the leaves, the expression of defense‐related genes was reduced in the TAS3i flowers compared to the WT flowers (Tables S11, S13, S17, and S18). Conversely, except for four genes related to adenylate cyclase activity and cAMP biosynthesis, none were significantly upregulated in the TAS3i flowers compared to the WT flowers (Tables S11, S13, S17, and S18). In the TAS3i buds, the genes related to chitin metabolism and pollen outer wall formation were upregulated compared to WT plants (Tables S11, S14, S19, and S20). These findings indicate that these genes might be associated with the miR390‐*TAS3* pathway. The expression of anther‐related genes was downregulated in the TAS3i plants, similar to *rdr6* plants, but that of spermidine biosynthesis‐related genes was unaffected in the buds of TAS3i plants, unlike the rdr6 buds.

The results of the DEG and GO analyses were compared between *rdr6* and TAS3i plants (Tables S21–S30). In the TAS3i plants, auxin‐activated signaling pathway‐related genes were downregulated in all tested tissues compared to those in the *rdr6* plants (Table S21). Several gene groups upregulated in the *rdr6* plants compared to the WT plants were downregulated in the TAS3i plants (Tables S10 and S21). While there was no clear trend in the expression of upregulated genes in the TAS3i plants compared to the *rdr6* plants, the expression of photosynthesis‐related genes was higher. The expression levels of defense‐related genes that were upregulated in the *rdr6* leaves were higher in the TAS3i leaves than in the *rdr6* leaves.

No significant differences were observed in the *ARF* expression levels in the flowers of the WT, rdr6, and TAS3i plants (Table 2). However, the expression of *ARF3* and *ARF4* was downregulated in the TAS3i plants compared to the *rdr6* plants in all tested tissues, indicating the recovery of the miR390‐*TAS3*‐*ARF* pathway (Table 2). Furthermore, no differences were observed in the *ARF2* expression levels between *rdr6* and TAS3i plants in all tested tissues (Table 2). These results indicate that *ARF2* might be controlled differently than *ARF3/ARF4* in *N. benthamiana*.

### miRNA analysis

To determine the changes in the ta‐siARF levels in the leaves, flowers, and buds, miRNAs were purified from the WT, TAS3i, and *rdr6* plants. Table 3 shows the number of miRNA sequences detected from the ta‐siARF sequences (20–24 nt long). The abundance of many siRNAs in the WT plants was relatively low, while that of the U3 and U7 upstream sequences was relatively high in the flowers and buds. In contrast, all siRNA levels, including U3 and U7, were relatively low in all tissues in the *rdr6* plants, presumably because the *rdr6* knockout prevented the production of ta‐siARF. In the TAS3i plants, all siRNA levels were markedly elevated compared to those of *rdr6* plants, possibly because each siRNA was produced due to the formation of an RNA double‐strand from a ta‐siARF inverted repeat in *vivo*. The increased amounts of these siRNAs might alter the expression levels of *ARF*s, particularly *ARF3* and *4* (Table 2).

**Table 3:** The number of miRNA sequences detected arising from the ta‐siARF sequences (20–24 nt long).

	Sequence (nt)	Leaves			Flowers			Buds		
Upstream ta‐siRNA sequence	5′‐TCTTCTTGACCTTGTAAGACCTTT‐3′	Wild‐type	TAS3i	*rdr6*	Wild‐type	TAS3i	*rdr6*	Wild‐type	TAS3i	*rdr6*
U1	5′‐GTCTTCTTGACCTTGTAAGAC‐3′ (21)	0	46	0	3	142	0	3	43	0
U2	5′‐AAAAGGTCTTACAAGGTCAAGA‐3′ (22)	0	21	0	1	70	0	2	46	0
U3	5′‐AAAAGGTCTTACAAGGTCAAGAA‐3′ (23)	3	553	1	36	1505	3	29	1127	2
U4	5′‐AAGAAAAGGTCTTACAAGGTCAAG‐3′ (24)	0	25	0	6	59	0	4	28	0
U5	5′‐GAAAAGGTCTTACAAGGTCAAG‐3′ (22)	0	20	0	3	65	0	1	27	0
U6	5′‐AGGTCTTACAAGGTCAAGAA‐3′ (20)	0	196	0	7	874	0	2	377	2
U7	5′‐GGTCTTACAAGGTCAAGAAG‐3′ (20)	1	364	0	300	1666	1	80	647	4
U8	5′‐GGTCTTACAAGGTCAAGAAGA‐3′ (21)	0	45	0	5	93	0	3	105	0
U9	5′‐GTCTTACAAGGTCAAGAAGA‐3′ (20)	0	80	1	2	239	0	0	140	0
U10	5′‐GTCTTACAAGGTCAAGAAGAC‐3′ (21)	0	95	0	8	279	0	0	130	0

	Sequence (nt)	Leaves			Flowers			Buds		
Downstream ta‐siRNA sequence	5′‐TCTTGACCTTGTAAGACCCCG‐3′	Wild‐type	TAS3i	rdr6	Wild‐type	TAS3i	rdr6	Wild‐type	TAS3i	rdr6
D1	5′‐TCTTGACCTTGTAAGACCCCG‐3′ (21)	1	121	0	2	361	1	2	181	0
D2	5′‐TTCTTGACCTTGTAAGACCCC‐3′ (20)	1	129	0	4	581	1	4	281	1
D3	5′‐TTCTTGACCTTGTAAGACCCCG‐3′ (21)	1	8	0	1	37	0	0	5	0
D4	5′‐TTTCTTGACCTTGTAAGACCCC‐3′ (21)	1	4	0	0	48	0	0	28	0
D5	5′‐TTTCTTGACCTTGTAAGACCCCG‐3′ (22)	0	60	0	3	131	0	2	39	1
D6	5′‐TTTTCTTGACCTTGTAAGACCCC ‐3′(22)	0	27	0	1	98	0	0	50	0
D7	5′‐CTTGACCTTGTAAGACCCCGT‐3′ (21)	1	58	0	1	127	0	1	65	1
D8	5′‐TCTTGACCTTGTAAGACCCCG‐3′ (21)	1	121	0	2	361	1	2	181	0
D9	5′‐TTCTTGACCTTGTAAGACCCCG‐3′ (22)	1	8	0	1	37	0	0	5	0
D10	5′‐TTTCTTGACCTTGTAAGACCCCG‐3′ (23)	0	60	0	3	131	0	2	39	1
D11	5′‐ACGGGGTCTTACAAGGTCAAG‐3′ (21)	0	55	0	3	163	0	3	89	0

### Productivity of recombinant GFP and FGF1 in the TAS3i plants

**Figure 4:**
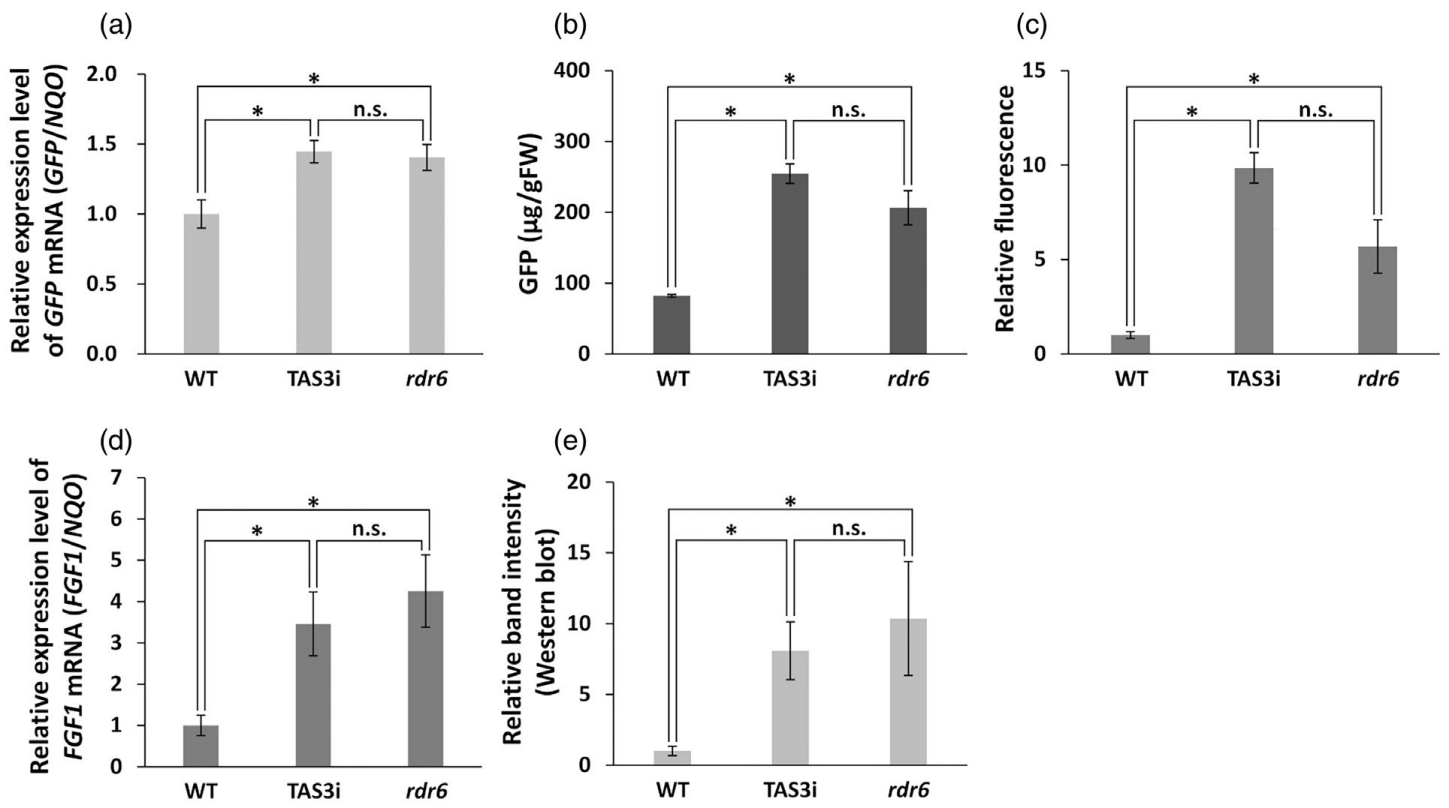
Transient expression of GFP and human FGF1 in WT, TAS3i, and *rdr6* plants. GFP and human FGF1 were transiently expressed in WT, TAS3i, and *rdr6* plants, and the infiltrated leaves were harvested at 5 dpi. (a) The relative expression level of GFP mRNA was estimated by real‐time RT‐PCR using *NAD(P)H dehydrogenase (quinone)* (*NQO*) as the housekeeping gene. (b) The amount of GFP protein was estimated using SDS‐PAGE followed by Coomassie blue staining. (c) Relative GFP fluorescence was measured by 20‐fold diluting the centrifuged supernatant of the crude extract (prepared in PBS at five times the volume of the leaves) from GFP‐expressing leaves. (d) The relative expression level of FGF1 mRNA was estimated by real‐time RT‐PCR using *NQO* as the housekeeping gene. (e) Relative band intensities of FGF1 were estimated from Western blot analysis. Data are shown as mean ± standard deviation (*n* = 3). *t*‐test; *P < 0.05; n.s., not significant.

To confirm the productivity of recombinant proteins in the TAS3i plants, GFP was transiently expressed in the WT, TAS3i, and *rdr6* plants using vacuum infiltration (Figure 4; Figure S5a). Real‐time Reverse Transcription Polymerase Chain Reaction (RT‐PCR) analysis of the GFP mRNA expression levels in the tested plants at 5 dpi indicated that the GFP mRNA levels in the TAS3i and *rdr6* plants were approximately 1.45 and 1.40‐fold higher than those of the WT plants (Figure 4a). As estimated by SDS‐PAGE followed by Coomassie staining, the amounts of GFP expression in the WT, TAS3i, and *rdr6* plants were 82.2, 254.6, and 206.6 μg/gFW, respectively (Figure 4b; Figure S5a), indicating that the TAS3i plants expressed larger amounts of recombinant proteins than the WT and *rdr6* plants.

To evaluate the potential of TAS3i plants for recombinant protein production, we performed a transient expression assay of the human FGF1 gene using hydroponically grown plants (Figure 4; Figure S5). At 5 dpi, TAS3i plants exhibited FGF1 mRNA levels that were approximately 3.46‐fold higher than in WT plants (Figure 4d). Moreover, FGF1 protein accumulation in TAS3i plants was found to be 8.09‐fold higher than in WT plants (Figure 4e; Figure S5b,c), demonstrating enhanced recombinant protein production in the TAS3i line.

## DISCUSSION

Previously, we reported that RNA‐silencing machinery‐repressed plants produced larger amounts of recombinant proteins than WT plants (Matsuo, 2021; Matsuo & Atsumi, 2019; Matsuo & Matsumura, 2017). However, these plants, including the *rdr6* plants, had disadvantages, such as sterility. Although the *rdr6* plants can be obtained from the seeds of chimera plants in which the *RDR6* gene is not completely knocked out (Matsuo & Atsumi, 2019), only about 20% of the seedlings turned into *rdr6* plants. Conversely, all the progeny of certain TAS3i lines possessed the NbTAS3i‐expression cassettes, making the production of TAS3i plants easy. Furthermore, the TAS3i plants were fertile, unlike the *rdr6* plants (Figure 3; Figures S3 and S4). Because almost all seeds from the TAS3i plants germinated, it is easy to expand the cultivation scale of the TAS3i plants to mass‐produce recombinant proteins.

The GFP mRNA levels in the TAS3i and *rdr6* plants were approximately 1.4 times that in the WT. However, the amounts of recombinant GFP expressed in the TAS3i and *rdr6* plants were about 3.1 and 2.5 times higher than those in WT plants, respectively. After centrifugation, the GFP fluorescence in the supernatant also confirmed the high productivity of recombinant proteins in TAS3i plants, indicating that the repression of RNA‐silencing machinery was maintained in the TAS3i plants, like the *rdr6* plants. However, the GFP mRNA and recombinant GFP amounts in the WT, TAS3i, and *rdr6* plants were not correlated, probably owing to the high stability of GFP. In contrast, recombinant human FGF1 accumulation in TAS3i and *rdr6* plants was approximately 8.09‐fold and 10.36‐fold higher than WT plants, respectively (Figure 4; Figure S5). Correspondingly, FGF1 mRNA levels in TAS3i plants were 3.46 times higher than in WT plants. Although the protein and mRNA levels of TAS3i plants were lower than those of *rdr6* plants, in contrast to the trend seen for GFP, TAS3i plants maintained an enhanced ability to produce recombinant proteins. Furthermore, transient expression of FGF1 in hydroponically grown TAS3i plants confirmed their superior productivity relative to WT. This finding was consistent with results obtained for GFP expression in soil‐grown TAS3i plants. Overall, our findings indicate that TAS3i plants grown under a variety of growing conditions can be used for recombinant protein production. As the sizes of the body and leaves of the TAS3i plants were similar to those of the WT plants (Figure 3), higher levels of recombinant protein can be produced per plant. This emphasizes the potential of the TAS3i plants for efficient recombinant protein production. In this study, the pRI201‐AN vector was used to express recombinant proteins. It remains unknown whether the pRI201‐AN vector has ever been used to produce medical proteins in plants. Owing to its wide application in research, we hypothesize that this vector will be used to produce medical proteins in plants in the future.

The miR390‐*TAS3*‐*ARF* pathway is critical for modulating leaf morphology, developmental transitions, flower and root architecture, embryo development, responses to biotic and abiotic stresses, and phytohormone crosstalk (Deng et al., 2018). *RDR6* and *DCL4* are crucial for the miR390‐*TAS3*‐*ARF* pathway. Unlike the *rdr6* plants, the *dcl2dcl4* plants were not sterile despite the lack of *DCL4*. As *DCL1* was shown to be involved in the generation of ta‐siRNA (Williams et al., 2005; Wu et al., 2012), *DCL1* or other components might complement the function of *DCL4* in the *dcl2dcl4* plants. Several differences might exist between the WT, *rdr6*, TAS3i, and *dcl2dcl4* plants. Elucidating these differences in future studies will benefit plant research.

The negative effects of the *RDR6* gene‐knockout, especially on fertility, plant height, and leaf size, were recovered by the expression of an inverted repeat of the Nb*TAS3* sequence. This observation revealed that *RDR6* is closely associated with the miR390‐*TAS3*‐*ARF* pathway. Several reports have shown the expression of artificial siRNA in plants (Cisneros & Carbonell, 2020; Zhang, 2014). In previous studies, dsRNAs were synthesized in plant cells via endogenous RDRs, including *RDR6*. To the best of our knowledge, this paper is the first study to report the restoration of the miR390‐*TAS3*‐*ARF* pathway in the absence of *RDR6* in *N. benthamiana*. We expressed the *NbTAS3* sequence‐derived dsRNA by expressing an inverted repeat construct of the *NbTAS3* sequence in the plants containing an *RDR6* knockout. This production allowed downstream processing by Dicer‐like enzymes, similar to RNA interference, without *RDR6*. Understanding the mechanism through which the TAS3 inverted repeat construct is processed in TAS3i plant bodies is an important challenge for the future. However, genetic manipulation of other downstream components, such as *ARF3* and *ARF4*, would provide stronger evidence and help establish a causal relationship between miR390‐*TAS3*‐*ARF* pathway disruption and the resulting phenotype. However, modulation of *ARF3* and *ARF4* is technically challenging and can lead to unintended phenotypic effects. In contrast, the introduction of the TAS3i inverted repeat construct can mimic the natural regulatory network (i.e., the miR390‐*TAS3*‐*ARF* pathway), allowing for gradual adjustment of expression levels while avoiding side effects associated with overexpression or complete knockout. In addition, inverted repeat constructs are a well‐established method for inducing small RNAs efficiently and stably in plant cells. Therefore, we selected this strategy as a practical and broadly applicable solution that is compatible with the *rdr6* mutant background. *RDR6* is also involved in the generation of other siRNAs, which may account for differences in RNA‐seq profiles between WT and *rdr6* plants. We also observed differences in RNA‐seq profiles between TAS3i and WT plants, suggesting that regeneration of the miR390‐*TAS3*‐*ARF* system alone is not sufficient to restore changes between WT and *rdr6* mutants. Thus, a more careful comparison of WT and *rdr6* plants may provide a better understanding of *RDR6* function.

Quantitative analysis of the *ARF*s in the WT, TAS3i, and *rdr6* plants revealed that the expression levels of *ARF2* differed from those of *ARF3* and *ARF4* (Table 2). RNA‐Seq analysis showed no significant differences in the expression levels of *ARF2* between WT, *rdr6*, and TAS3i plants, indicating that the changes in *ARF2* expression levels in the transgenic plants might be minimal compared to those of *ARF3* and *ARF4*, as reported previously (Adenot et al., 2006). However, the expression levels of *ARF3/4* were greatly increased in the *rdr6* plants, but significantly reduced in the TAS3i plants compared to those of WT and *rdr6* plants. These observations suggest that the regulation mechanism for *ARF2* might be different from those of *ARF3* and *ARF4* in *N. benthamiana*. Although these *ARF*s are considered a single set, many studies have examined only *ARF3* and *ARF4*, not *ARF2* (Hunter et al., 2006; Matsui et al., 2014; Yifhar et al., 2012), indicating the differences between *ARF2* and the others in *N. benthamiana*. The levels of siRNA against ta‐siRNA sequences in the respective plants were also determined. Consequently, lower levels of ta‐si*ARF*s were observed in the *rdr6* plants than in the WT plants. However, the generation of artificial double‐stranded RNA containing the ta‐si*ARF* sequence increases ta‐si*ARF*s despite the *rdr6* knockout (Table 3). As these alterations were particularly pronounced in the flowers and buds, they might be responsible for restoring the fertility of the TAS3i plants. The difference noted between the ta‐siRNA levels in the WT and *rdr6* leaves was minor, although the ta‐siRNA levels were slightly higher in the WT plants than in the *rdr6* plants. Thus, the amount of ta‐siRNA required for determining leaf morphology might be minimal. Interestingly, genes presumed to be directly or indirectly related to *ARF*s, including *TEX1*, *YABBY* (*YAB*), *auxin‐responsive protein IAA* (*IAA*), and *KANADI* family genes (*KAN*), were not found in our DEG list. These observations can be attributed to the timing of sample collection or to differences in the miR390‐*TAS3*‐*ARF* pathway between *A. thaliana* and *N. benthamiana* and are a topic for future investigation. Although transcriptome data for ta‐siRNA‐insensitive *ARF N. benthamiana* mutants are currently unavailable, a comparative analysis with existing *Arabidopsis* datasets (e.g., ARF3m mutants) may offer meaningful information, especially regarding conserved target genes and regulatory patterns. Thus, a comparison between both datasets is also an important subject for future work.

This study revealed new findings on the sterility of *rdr6* plants. As previously reported, *rdr6* plants are naturally sterile (Ludman & Fátyol, 2019; Matsuo & Atsumi, 2019). The *rdr6* plants showed variations in gene expression related to flower, anther, and pollen formation likely affecting floral morphology (Figures S3 and S4). In *rdr6* plants, the pistil was shortened but was restored to WT levels in TAS3i plants (Figure S6). Additionally, while WT and TAS3i flowers pointed diagonally upward toward the ground, *rdr6* flowers were horizontal (Figure S7). These differences were assumed to cause sterility. Accordingly, artificial pollination was performed. As a result, *rdr6* plants produced seeds; however, they were smaller than those of WT and TAS3i plants (Figure S8). This may be due to altered pollen‐related gene expression only in buds, not flowers. The germination rate of *rdr6*‐derived seeds was 100% (60/60), suggesting that sterility is caused by abnormal floral morphology, including pistil length.

In this study, we successfully produced TAS3i plants with high recombinant protein productivity and fertility. Moreover, we reconfirmed the significant relationship between *RDR6* and the miR390‐*TAS3*‐*ARF* pathway, and used *rdr6* and TAS3i plants to conduct a detailed examination of the functions of *RDR6* and the miR390‐*TAS3*‐*ARF* pathway in plants. Furthermore, we have produced a variety of transgenic *N. benthamiana* plant lines, and seeds of these plants are available by request following the conclusion of a material transfer agreement with our institute.

## MATERIALS AND METHODS

### Construction of an expression vector for the inverted repeat of the *NbTAS3* sequence

Genomic DNA was purified from 100 mg of *N. benthamiana* and *Arabidopsis thaliana* leaf tissues using the GeneJET Plant Genomic DNA Purification Mini Kit (Thermo Fisher Scientific, Waltham, MA, USA) according to the manufacturer's instructions. For the polymerase chain reaction (PCR), a 25 μL reaction solution of KOD FX Neo (Toyobo, Osaka, Japan) containing 1 μL genomic DNA and 5 pmol of each primer was used. The primer pair NbTAS3‐F (5′‐CATCTGCACCTGCAACTTCTTC‐3′) and NbTAS3‐R (5′‐GCATTAGTACCACTCTTGATGAC‐3′) was designed based on a previously reported sequence (Krasnikova et al., 2009) and used to amplify the *NbTAS3* sequence from the *N. benthamiana* genomic DNA. Amplified PCR products were directly cloned into a pTA2 vector using TArget Clone‐Plus (Toyobo). To construct the dsRNA expression vectors (pNbTAS3i), the inverted repeat construct of the *NbTAS3* sequence (NbTAS3i) was generated on the binary vector pBE2113 (Mitsuhara et al., 1996) using an intron sequence of the β‐1,2‐xylosyltransferase gene from *A. thaliana* (AtXTint) (Matsuo & Matsumura, 2011; Matsuo & Matsumura, 2017). *Agrobacterium tumefaciens* strain LBA4404 was transformed with the pNbTAS3i vector and used for leaf disk transformation of the *RDR6* gene‐knockout *N. benthamiana*, *rdr6* plant (Matsuo & Atsumi, 2019) to generate the NbTAS3i‐expressed *rdr6* plants (TAS3i plants). The integration of the introduced inverted repeat construct into the transgenic plants was confirmed with conventional direct genomic PCR (Matsuo, 2021) using the following primers: T3inv‐RT‐F (5′‐CATTCTACAACTACATCTAGACATC‐3′) and T3inv‐RT‐R (5′‐CTTCAAATCCACCAGATCAATGAG‐3′) and the Extract‐N‐Amp Plant PCR Kit (Sigma‐Aldrich, St. Louis, MO, USA) according to the manufacturer's instructions with some modifications (Matsuo, 2021).

### Transient expression of GFP and human FGF1

For the transient expression of GFP, agroinfiltration was performed as previously described (Matsuo, 2022). *Rhizobium radiobacter* (*Agrobacterium tumefaciens*) LBA4404 strains along with pBE2113:GFP (Matsuo & Matsumura, 2017; Matsuo & Atsumi, 2019; Matsuo, 2022) was pre‐cultured in 50 mL of yeast extract peptone (YEP) medium containing kanamycin (50 μg/mL), streptomycin (300 μg/mL), and rifampicin (100 μg/mL) overnight at 28°C with vigorous shaking. The resulting pre‐cultures were then added to 750 mL of YEP medium containing antibiotics and cultured overnight at 28°C with vigorous shaking. The *Rhizobium* cell pellet was obtained by centrifuging at 5000 ***g*** for 10 min at 23°C, which was then resuspended in 10 mM MES‐KOH (pH 5.7) containing 10 mM MgCl_2_ and 150 μM acetosyringone. The optical density was adjusted to 0.5. The *Rhizobium* solution was kept at 23°C–25°C for at least 2 h before infiltration. The T_1_ transgenic TAS3i, T_3_ transgenic *rdr6*, and WT *N. benthamiana* plants used for the agroinfiltration were cultivated in a greenhouse at room temperature (23°C–25°C). All plants were subjected to agroinfiltration 4 weeks after seeding. These plants were submerged in the *Rhizobium* suspension and placed in a vacuum chamber at room temperature. The vacuum was maintained for 1 minute and then released rapidly. Following vacuum infiltration, the plants were placed in a growth chamber at 23°C under a 16/8 h light/dark cycle. Three individuals from the WT, TAS3i, and *rdr6* plant groups were used for this experiment. To assess the expression of human fibroblast growth factor 1 (FGF1) (NCBI Accession No. NM_000800), leaves were infiltrated with *R. radiobacter* strain LBA4404 carrying the pBE2113:FGF1 construct using needleless plastic syringes, as described by Matsuo (2022). In addition, LBA4404 harboring pBE2113:GFP was also infiltrated as a negative control. In both cases, at 5 days after infiltration, infiltrated leaves were collected and then subjected to further experiments.

### mRNA quantification using real‐time RT‐PCR

Total RNA was extracted from the leaves, flowers, and buds using the RNeasy Plant Mini Kit (Qiagen, Hilden, Germany). A 50 mg fully expanded leaf tissue (Figure 3a), five flowers in anthesis, including sepal (Figures 3b,c), and five buds (sampled prior to petal emergence) from each plant were used for total RNA extraction. The DNA contamination in the total RNA samples was removed using TURBO DNase (Thermo Fisher Scientific, Waltham, MA, USA). The first‐strand cDNA was synthesized from 1 μg of total RNA using the PrimeScript II 1st strand cDNA Synthesis Kit (Takara Bio, Otsu, Japan) with 50 pmol of random hexamer primers. Real‐time RT‐PCR was performed using a 10 μL reaction volume containing 2.5 μL of each RT reaction mixture diluted 100‐fold, 2 pmol of the respective primers, and FastStart Essential DNA Green Master Mix (Roche, Basel, Switzerland). The relative expression level of the GFP gene was calculated using the 2^−ΔΔCq^ method, using the *NAD(P)H dehydrogenase (quinone) (NQO)* gene as a housekeeping gene (Pombo et al., 2019). For respective gene‐specific amplification, the following primer sets were used: GFP‐F (5′‐GAGGGATACGTGCAGGAGAG‐3′) and GFP‐R (5′‐ATCCTGTTGACGAGGGTGTC‐3′), FGF1‐F (5′‐TGGAAAGGCTGGAGGAGAAC‐3′) and FGF1‐R (5′‐CGTTTGCAGCTCCCATTCTTC‐3′), and NbNQO‐F (5′‐AAGGCGGTGGTCAAGAAA‐3′) and NbNQO‐R (5′‐CAAACATACCAGCACCGAATG‐3′).

### RNA‐seq analysis and GO analysis

Total RNA was extracted from the leaves, flowers, and buds using the method described in the previous section. For RNA‐seq analysis, tissue samples were collected from WT, *rdr6*, and TAS3i plants. Approximately 50 mg of fully expanded leaf tissue (Figure 3a), one flower in anthesis, including sepal (Figures 3b,c), and three buds (sampled prior to petal emergence) were harvested from each plant. All samples were taken 53 days after seeding. Total RNA was then extracted from the corresponding tissues of four WT and *rdr6* plants and five TAS3i plants. RNA was pooled by tissue type (i.e., leaf, flower, and bud) before being subjected to RNA‐seq analysis.

Next‐generation sequencing (NGS) was performed using the total RNAs at the Bioengineering Lab (Sagamihara, Kanagawa, Japan). The concentrations of the RNA samples were measured using the Synergy LX (Bio Tek, Winooski, VT, USA) and QuantiFluor RNA system (Promega, Madison, WI, USA) and quality‐checked with the Agilent HS RNA kit (Agilent Technologies, Santa Clara, CA, USA) using the 5200 Fragment Analyzer System. The NGS library was prepared using the MGIEasy RNA Directional Library Prep Set (MGI Tech, Shenzhen, China) according to the manufacturer's protocol. The concentrations of the library were measured using Synergy LX and QuantiFluor dsDNA System. The library was quality‐checked with a Fragment Analyzer and dsDNA 915 Reagent Kit (Agilent Technologies). Circular DNA was constructed from the library using the MGIEasy Circularization Kit (MGI Tech). The DNA nanoball (DNB) was made with the DNBSEQ‐G400RS High throughput Sequencing kit (MGI Tech) according to the manufacturer's protocol. Sequencing was performed with DNBSEQ‐G400 for at least 2 × 100 bp reads. The adapter sequences from the delivered fastq files were removed using cutadapt (ver. 4.0). The short‐read sequences under 20 and low‐quality score reads (under 40) were removed with a sickle (ver. 1.33). HISAT2 (ver. 2.2.1) was used to analyze the alignment and mapping of the *N. tabacum* reference genome Ntab‐TN90 (NCBI Reference sequence: GCF_000715135.1). After reading and writing the alignment data in the SAM and BAM formats with Samtools, the data were sorted and indexed. The read counts per gene were obtained using featureCounts (ver. 1.16.1). The relative expression levels of each gene were normalized by the reads per kilobase million and transcripts per million methods. The registered sequences were compared with the sequences in the GO database (geneontology.org/) using the BLASTX command (very sensitive mode) in Diamond (ver. 2.0.4). The GO terms were assigned to the gene sequences (*E*‐value <10^−5^). The up‐ and downregulated genes were listed and subjected to Fisher's exact test.

### miRNA‐seq analysis

miRNA was extracted from the leaves, flowers, and buds using a microRNA Purification Kit (Norgen Biotek Corp., Thorold, Canada). For miRNA‐seq analysis, approximately 20 mg of fully expanded leaf tissue (Figure 3a), one flower in anthesis with sepal (Figures 3b,c), and three buds (sampled prior to petal emergence) from three members of the WT, *rdr6*, and TAS3i plant groups were collected. Samples were taken from plants 55 days after seeding. miRNAs were then extracted from each tissue type for each genotype and pooled by tissue and genotype before being subjected to miRNA sequencing. Finally, miRNA‐seq analysis was performed at a Bioengineering Lab (Sagamihara, Kanagawa, Japan). DNA contamination in the miRNA was digested with RNA Clean & Concentrator‐5 with DNase I (Zymo Research, Irvine, CA, USA). The miRNA was quality‐checked using a Fragment Analyzer and HS RNA kit (Agilent Technologies). The library was prepared using the MGIEasy Small RNA Library Prep Kit (MGI Tech, Shenzhen, China) according to the manufacturer's protocol. Size selection of the library was performed using MGIEasy DNA Clean Beads (MGI Tech). The concentrations of the library were measured with a Qubit 3.0 Fluorometer and dsDNA HS assay kit (Thermo Fisher Scientific) and quality‐checked with the High Sensitivity DNA kit (Agilent Technologies) using the Agilent 2100 Bioanalyzer. Circular DNA was made from the library using the MGIEasy Small RNA Library Prep Kit (MGI Tech) according to the manufacturer's protocol. DNA nanoball (DNB) was made using the DNBSEQ‐G400RS High throughput Sequencing kit (Small RNA FCL SE50, MGI Tech) according to the manufacturer's protocol. The DNBSEQ‐G400 was sequenced for 1 × 50 bp read. The adapter sequences from the delivered fastq files were removed using cutadapt (ver. 4.0). The short‐read sequences under 15 and low‐quality score reads (under 20) were removed with a sickle (ver. 1.33). TopHat2 (ver. 2.2.1) was used to read the alignment and mapping of the *N. benthamiana* reference genomes (Kurotani et al., 2025; https://nbenthamiana.jp/).

### Other materials and methods

For sodium dodecyl sulfate‐polyacrylamide gel electrophoresis (SDS‐PAGE) and Western blot analysis, we first ground 100 mg of leaf samples with 500 μL of phosphate‐buffered saline (PBS). The resultant suspension was centrifuged at 23000 ***g*** for 10 min at 4°C. Then, 5 μL of the supernatant was loaded onto a 15% (w/v) SDS‐PAGE gel (Atto, Tokyo, Japan). The separated proteins were stained using the GelCode Blue Safe Protein Stain (Thermo Fisher Scientific). For Western blot analysis, proteins separated by electrophoresis were first transferred onto a PVDF membrane (Atto). To detect human FGF1 expression, anti‐FGF‐1 (B‐3), human (mouse) (Santa Cruz Biotechnology, Dallas, TX, USA, #SC‐55520) was used as the primary antibody. Antimouse Immunoglobulin G, peroxidase‐linked species‐specific whole antibodies from sheep (GE Healthcare, Chicago, IL, USA; catalog #NA931) were used as secondary antibodies. The amount of recombinant GFP and FGF1 was estimated using the Image Lab software (Bio‐Rad Laboratories, Hercules, CA, USA). For transient expression of GFP and observation of morphological changes in the TAS3i plants, the TAS3i, *rdr6*, and WT *N. benthamiana* plants were cultivated in a greenhouse through soil cultivation at room temperature (23°C–25°C). Flowers and ovaries (before enlarged, with ovules, and ovule less) were counted at 91 days after seeding. For transient expression of FGF1, plants were hydroponically cultured in a greenhouse at room temperature (23°C–25°C) using Vegetable Life A (OAT AGRIO, Tokyo, Japan) hydroponic nutrient solution.

### Statistical analyses

Statistical analyses were performed using Microsoft Excel, including a Student's *t*‐test (*P* < 0.05) to compare the means of the two groups. When the number of plants is not the same, the t‐test was performed both under the assumption of equal variances and under the assumption that the variances were not equal. Data are presented as the mean ± standard deviation (SD).

## Accession Number

The RNA‐seq and miRNA‐seq analysis data have been deposited with links to BioProject accession numbers PRJDB16423 and PRJDB19185 in the DDBJ BioProject database (https://www.ddbj.nig.ac.jp/dra/index‐e.html), respectively.

## Supporting information


**Figure S1.** MA plot for differential expression analysis. Differentially expressed genes are indicated by pink circles.
**Figure S2.** The nucleotide sequence of the ta‐si*ARF* loci from *Nicotiana benthamiana* (Niben101Scf07184Ctg012:211877‐212 317).
**Figure S3.** Comparison of the number of flowers and ovaries between the wild‐type, *rdr6*, and TAS3i plants.
**Figure S4.** Flowers and ovaries of WT, TAS3i, and *rdr6* plants photographed 91 days after seeding.
**Figure S5.** Transient expression of GFP and human FGF1.
**Figure S6.** Differences in pistil lengths of WT, TAS3i, and *rdr6* plants.
**Figure S7.** Flowers of WT, TAS3i, and *rdr6* plants.
**Figure S8.** Seeds of WT, TAS3i, and *rdr6* plants.

**Table S1.** Numbers of differentially expressed genes.
**Table S2.** Differentially expressed genes in flowers of wild‐type plants versus *rdr6* plants.
**Table S3.** Differentially expressed genes in buds of wild‐type plants versus *rdr6* plants.
**Table S4.** GO analysis of upregulated genes in leaf samples (wild‐type vs. *rdr6* plants).
**Table S5.** GO analysis of downregulated genes in leaf samples (wild‐type vs. *rdr6* plants).
**Table S6.** GO analysis of upregulated genes in flower samples (wild‐type vs. *rdr6* plants).
**Table S7.** GO analysis of downregulated genes in flower samples (wild‐type vs. *rdr6* plants).
**Table S8.** GO analysis of upregulated genes in bud samples (wild‐type vs. *rdr6* plants).
**Table S9.** GO analysis of downregulated genes in bud samples (wild‐type vs. *rdr6* plants).
**Table S10.** Top 10 gene ontology terms among differentially expressed genes^†^ (wild‐type vs. *rdr6*).
**Table S11.** Top 10 gene ontology terms among differentially expressed genes^†^ (wild‐type vs. TAS3i plants).
**Table S12.** Differentially expressed genes in leaves of wild‐type plants vs. TAS3i plants.
**Table S13.** Differentially expressed genes in flowers of wild‐type plants vs. TAS3i plants.
**Table S14.** Differentially expressed genes in buds of wild‐type plants vs. TAS3i plants.
**Table S15.** GO analysis of upregulated genes in leaf samples (wild‐type vs. TAS3i plants).
**Table S16.** GO analysis of downregulated genes in leaf samples (wild‐type vs. TAS3i plants).
**Table S17.** GO analysis of upregulated genes in flower samples (wild‐type vs. TAS3i plants).
**Table S18.** GO analysis of downregulated genes in flower samples (wild‐type vs. TAS3i plants).
**Table S19.** GO analysis of upregulated genes in bud samples (wild‐type vs. TAS3i plants).
**Table S20.** GO analysis of downregulated genes in bud samples (wild‐type vs. TAS3i plants).
**Table S21.** Top 10 gene ontology terms among differentially expressed genes^†^ (*rdr6* vs. TAS3i plants).
**Table S22.** Differentially expressed genes in leaves of *rdr6* plants versus TAS3i plants.
**Table S23.** Differentially expressed genes in flower samples of *rdr6* plants versus TAS3i plants.
**Table S24.** Differentially expressed genes in buds of *rdr6* plants versus TAS3i plants.
**Table S25.** GO analysis of upregulated genes in leaf samples (*rdr6* vs. TAS3i plants).
**Table S26.** GO analysis of downregulated genes in leaf samples (*rdr6* vs. TAS3i plants).
**Table S27.** GO analysis of upregulated genes in flower samples (*rdr6* vs. TAS3i plants).
**Table S28.** GO analysis of downregulated genes in flower samples (*rdr6* vs. TAS3i plants).
**Table S29.** GO analysis of upregulated genes in bud samples (*rdr6* vs. TAS3i plants).
**Table S30.** GO analysis of downregulated genes in bud samples (*rdr6* vs. TAS3i plants).

## Data Availability

The data that support the findings of this study are openly available in the DNA Data Bank of Japan (DDBJ) at reference numbers PRJDB16423, PRJDB19185. https://urldefense.com/v3/__https://ddbj.nig.ac.jp/search/entry/bioproject/PRJDB16423__;!!N11eV2iwtfs!uXd5qFs4KahflnlKZ3YPreM80SiUpDLU_F‐fhL‐SLhXB5kQp0kchUF0uy‐8YJ0DEZ_r2kMhqODU7P4Pa8uz56CzPqQ4$, https://urldefense.com/v3/__https://ddbj.nig.ac.jp/search/entry/bioproject/PRJDB19185__;!!N11eV2iwtfs!uXd5qFs4KahflnlKZ3YPreM80SiUpDLU_F‐fhL‐SLhXB5kQp0kchUF0uy‐8YJ0DEZ_r2kMhqODU7P4Pa8uz5bXOJJOA$.
