# Supplementary material for: Recovery of RNA‐dependent RNA polymerase 6 gene‐knockout phenotypes in Nicotiana benthamiana via in vivo generation of inverted repeat construct of the trans‐acting short interference RNA3 sequence
Source: Plant J. 2025 Jul 18;123(2):e70350. doi: 10.1111/tpj.70350 (PMC12274081; doi:10.1111/tpj.70350)
Supplement: Supplementary file 1 — Figure S1. MA plot for differential expression analysis. Differentially expressed genes are indicated by pink circles. Figure S2. The nucleotide sequence of the ta‐siARF loci from Nicotiana benthamiana (Niben101Scf07184Ctg012:211877‐212 317). Figure S3. Comparison of the number of flowers and ovaries between the wild‐type, rdr6, and TAS3i plants. Figure S4. Flowers and ovaries of WT, TAS3i, and rdr6 plants photographed 91 days after seeding. Figure S5. Transient expression of GFP and human FGF1. Figure S6. Differences in pistil lengths of WT, TAS3i, and rdr6 plants. Figure S7. Flowers of WT, TAS3i, and rdr6 plants. Figure S8. Seeds of WT, TAS3i, and rdr6 plants. [file TPJ-123-0-s001.pptx]

## Slide 1
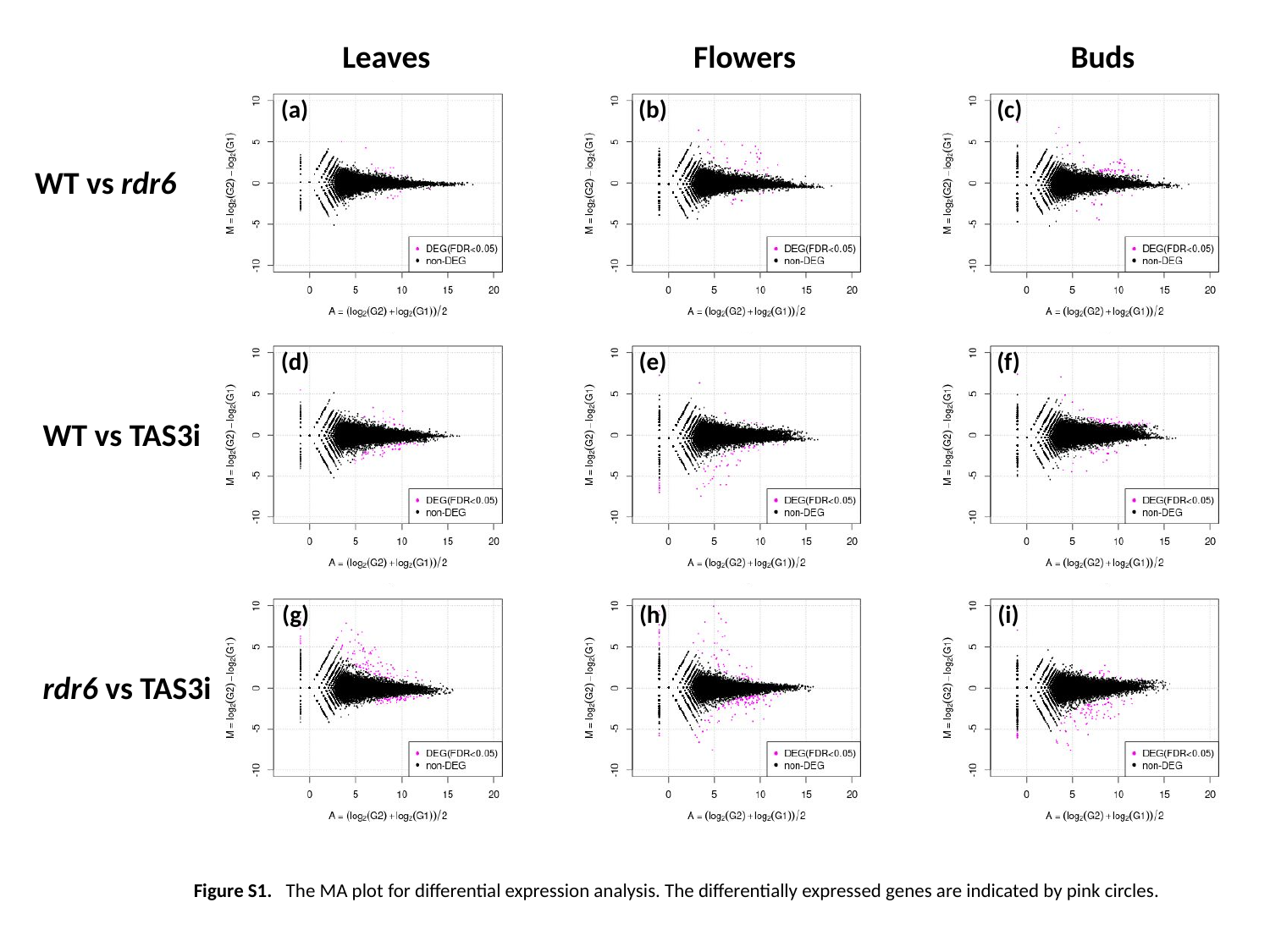

Leaves
Flowers
Buds
(a)
(b)
(c)
WT vs rdr6
(d)
(e)
(f)
WT vs TAS3i
(g)
(h)
(i)
rdr6 vs TAS3i
Figure S1. The MA plot for differential expression analysis. The differentially expressed genes are indicated by pink circles.

## Slide 2
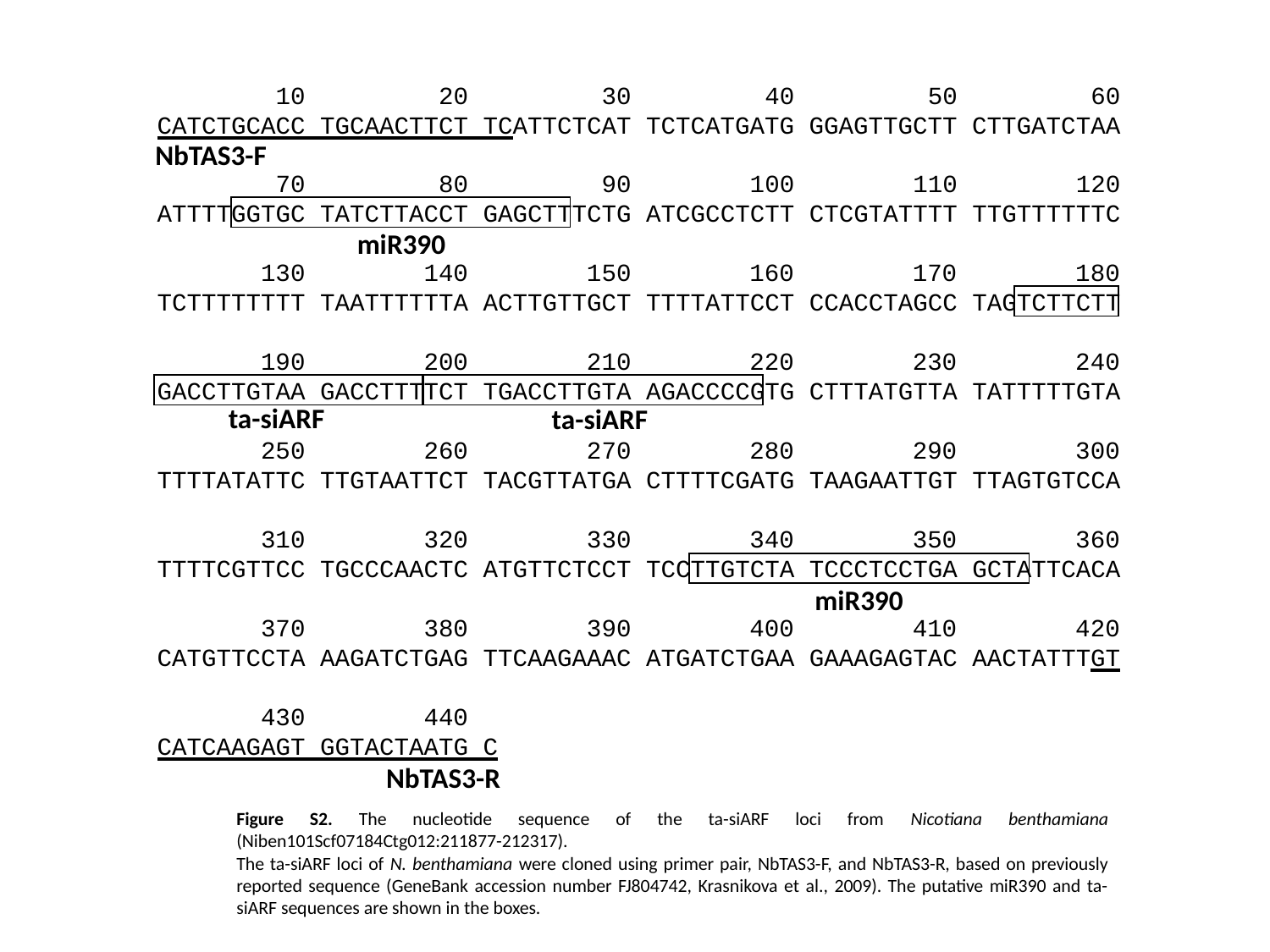

10 20 30 40 50 60
CATCTGCACC TGCAACTTCT TCATTCTCAT TCTCATGATG GGAGTTGCTT CTTGATCTAA
 70 80 90 100 110 120
ATTTTGGTGC TATCTTACCT GAGCTTTCTG ATCGCCTCTT CTCGTATTTT TTGTTTTTTC
 130 140 150 160 170 180
TCTTTTTTTT TAATTTTTTA ACTTGTTGCT TTTTATTCCT CCACCTAGCC TAGTCTTCTT
 190 200 210 220 230 240
GACCTTGTAA GACCTTTTCT TGACCTTGTA AGACCCCGTG CTTTATGTTA TATTTTTGTA
 250 260 270 280 290 300
TTTTATATTC TTGTAATTCT TACGTTATGA CTTTTCGATG TAAGAATTGT TTAGTGTCCA
 310 320 330 340 350 360
TTTTCGTTCC TGCCCAACTC ATGTTCTCCT TCCTTGTCTA TCCCTCCTGA GCTATTCACA
 370 380 390 400 410 420
CATGTTCCTA AAGATCTGAG TTCAAGAAAC ATGATCTGAA GAAAGAGTAC AACTATTTGT
 430 440
CATCAAGAGT GGTACTAATG C
NbTAS3-F
miR390
ta-siARF
ta-siARF
miR390
NbTAS3-R
Figure S2. The nucleotide sequence of the ta-siARF loci from Nicotiana benthamiana (Niben101Scf07184Ctg012:211877-212317).
The ta-siARF loci of N. benthamiana were cloned using primer pair, NbTAS3-F, and NbTAS3-R, based on previously reported sequence (GeneBank accession number FJ804742, Krasnikova et al., 2009). The putative miR390 and ta-siARF sequences are shown in the boxes.

## Slide 3
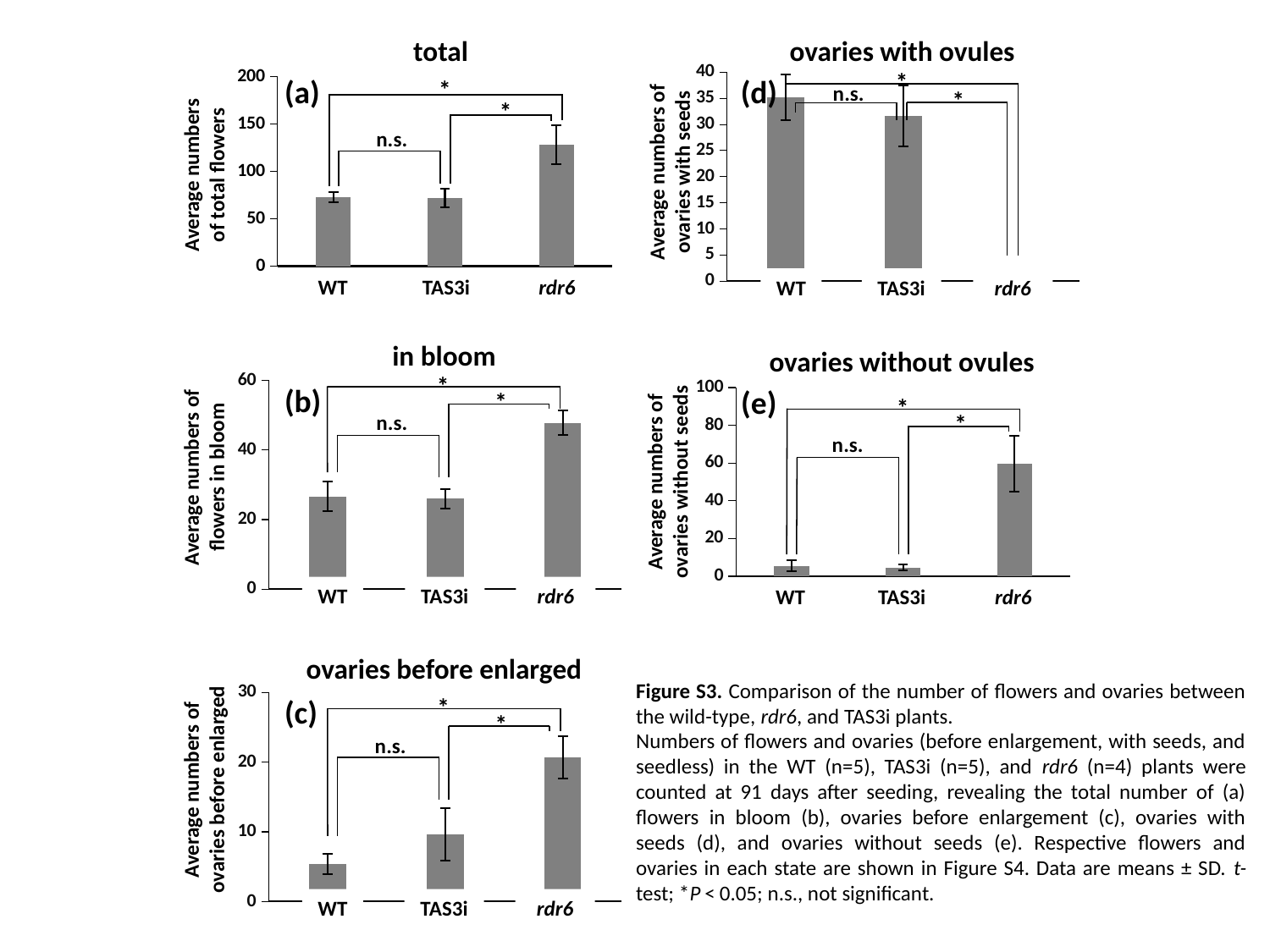

total
ovaries with ovules
### Chart
| Category | 種あり |
|---|---|
| WT | 35.2 |
| T3 | 31.6 |
| R6 | 0.0 |*
*
(d)
n.s.
Average numbers of ovaries with seeds
WT
TAS3i
rdr6
### Chart
| Category | 花の総計 |
|---|---|
| WT | 72.8 |
| T3 | 71.8 |
| R6 | 128.25 |(a)
*
*
n.s.
Average numbers of total flowers
WT
TAS3i
rdr6
in bloom
ovaries without ovules
*
*
### Chart
| Category | 咲いている花 |
|---|---|
| WT | 26.6 |
| T3 | 26.0 |
| R6 | 47.75 |(b)
n.s.
Average numbers of flowers in bloom
WT
TAS3i
rdr6
### Chart
| Category | 種無し |
|---|---|
| WT | 5.6 |
| T3 | 4.6 |
| R6 | 59.75 |(e)
*
*
n.s.
Average numbers of ovaries without seeds
WT
TAS3i
rdr6
ovaries before enlarged
### Chart
| Category | 中間 |
|---|---|
| WT | 5.4 |
| T3 | 9.6 |
| R6 | 20.75 |*
*
(c)
n.s.
Average numbers of ovaries before enlarged
WT
TAS3i
rdr6
Figure S3. Comparison of the number of flowers and ovaries between the wild-type, rdr6, and TAS3i plants.
Numbers of flowers and ovaries (before enlargement, with seeds, and seedless) in the WT (n=5), TAS3i (n=5), and rdr6 (n=4) plants were counted at 91 days after seeding, revealing the total number of (a) flowers in bloom (b), ovaries before enlargement (c), ovaries with seeds (d), and ovaries without seeds (e). Respective flowers and ovaries in each state are shown in Figure S4. Data are means ± SD. t-test; *P < 0.05; n.s., not significant.

## Slide 4
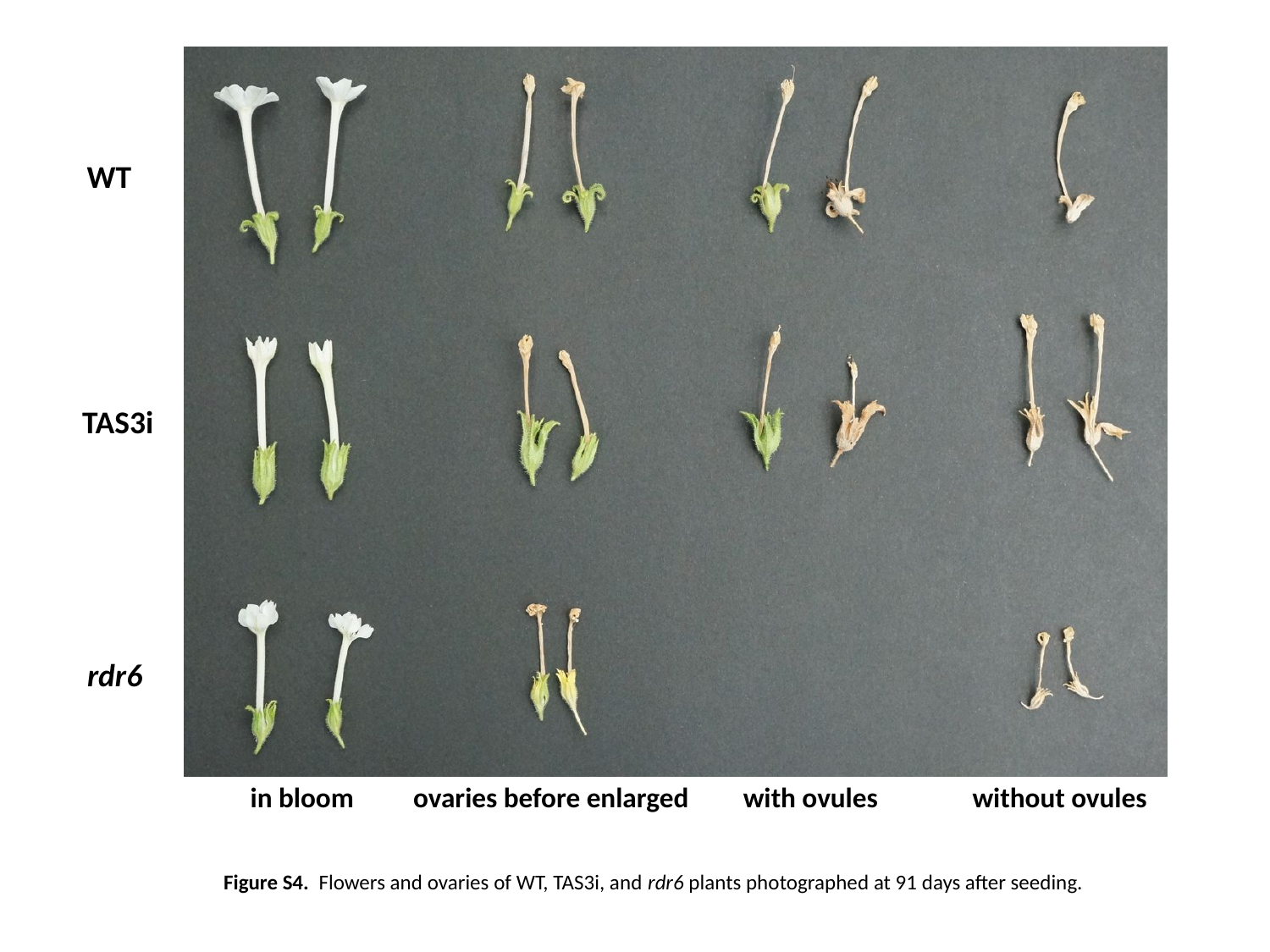

WT
TAS3i
rdr6
in bloom
ovaries before enlarged
with ovules
without ovules
Figure S4. Flowers and ovaries of WT, TAS3i, and rdr6 plants photographed at 91 days after seeding.

## Slide 5
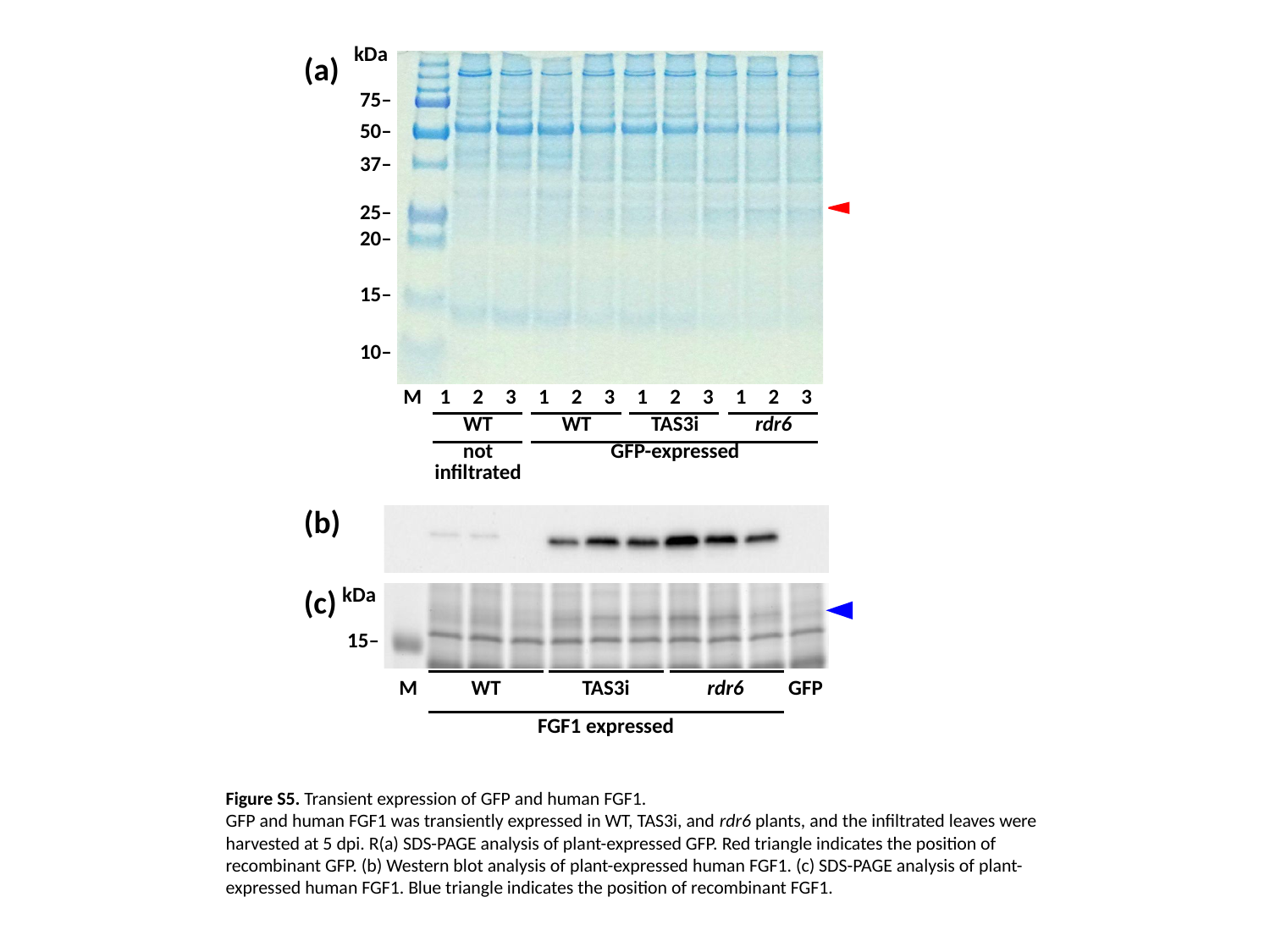

kDa
(a)
75–
50–
37–
25–
20–
15–
10–
| M | 1 | 2 | 3 | 1 | 2 | 3 | 1 | 2 | 3 | 1 | 2 | 3 |
| --- | --- | --- | --- | --- | --- | --- | --- | --- | --- | --- | --- | --- |
| | WT | | | WT | | | TAS3i | | | rdr6 | | |
| | not infiltrated | | | GFP-expressed | | | | | | | | |
(b)
kDa
(c)
15–
| M | WT | TAS3i | rdr6 | GFP |
| --- | --- | --- | --- | --- |
| | FGF1 expressed | | | |
Figure S5. Transient expression of GFP and human FGF1.
GFP and human FGF1 was transiently expressed in WT, TAS3i, and rdr6 plants, and the infiltrated leaves were harvested at 5 dpi. R(a) SDS-PAGE analysis of plant-expressed GFP. Red triangle indicates the position of recombinant GFP. (b) Western blot analysis of plant-expressed human FGF1. (c) SDS-PAGE analysis of plant-expressed human FGF1. Blue triangle indicates the position of recombinant FGF1.

## Slide 6
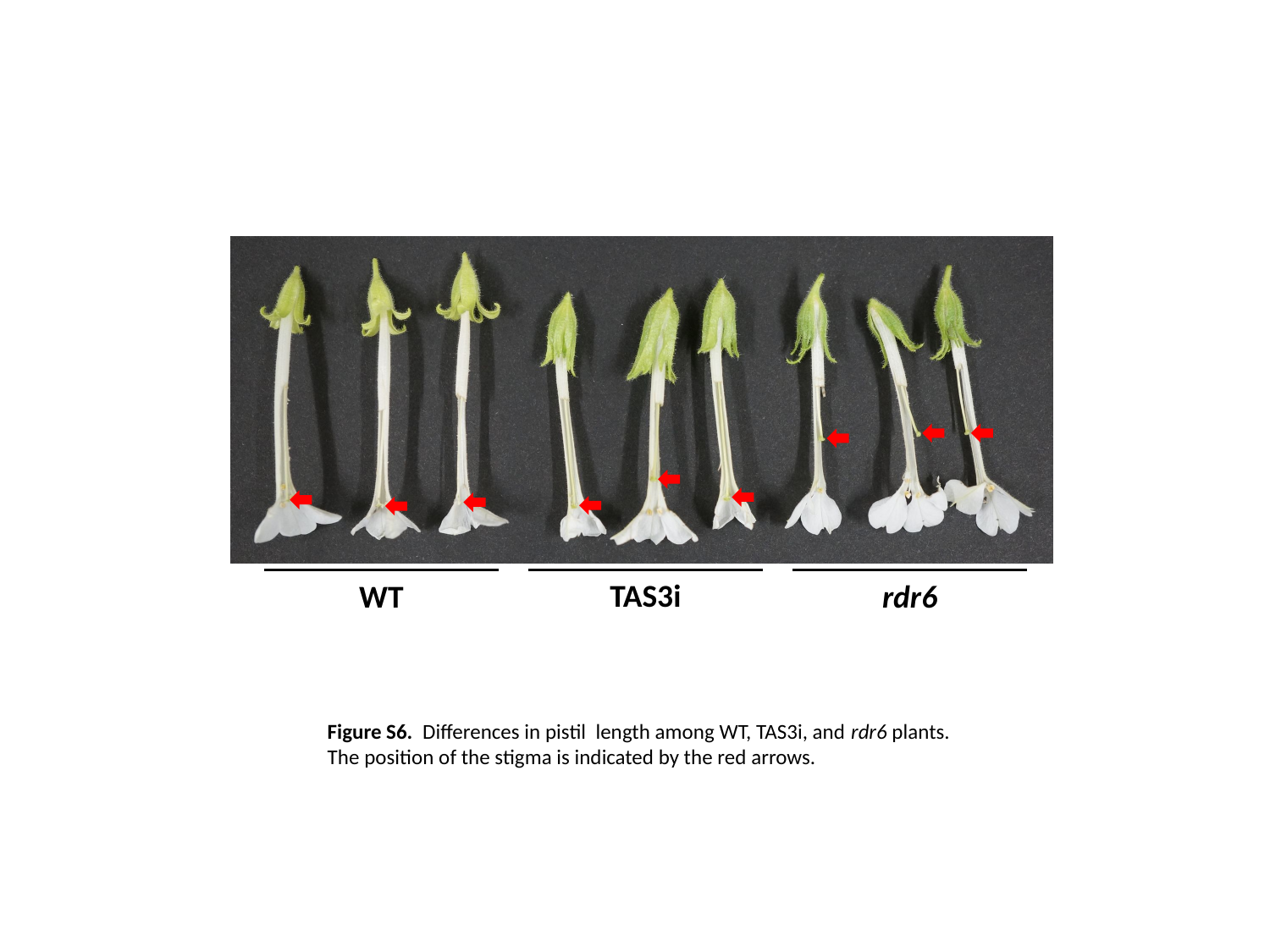

TAS3i
WT
rdr6
Figure S6. Differences in pistil length among WT, TAS3i, and rdr6 plants.
The position of the stigma is indicated by the red arrows.

## Slide 7
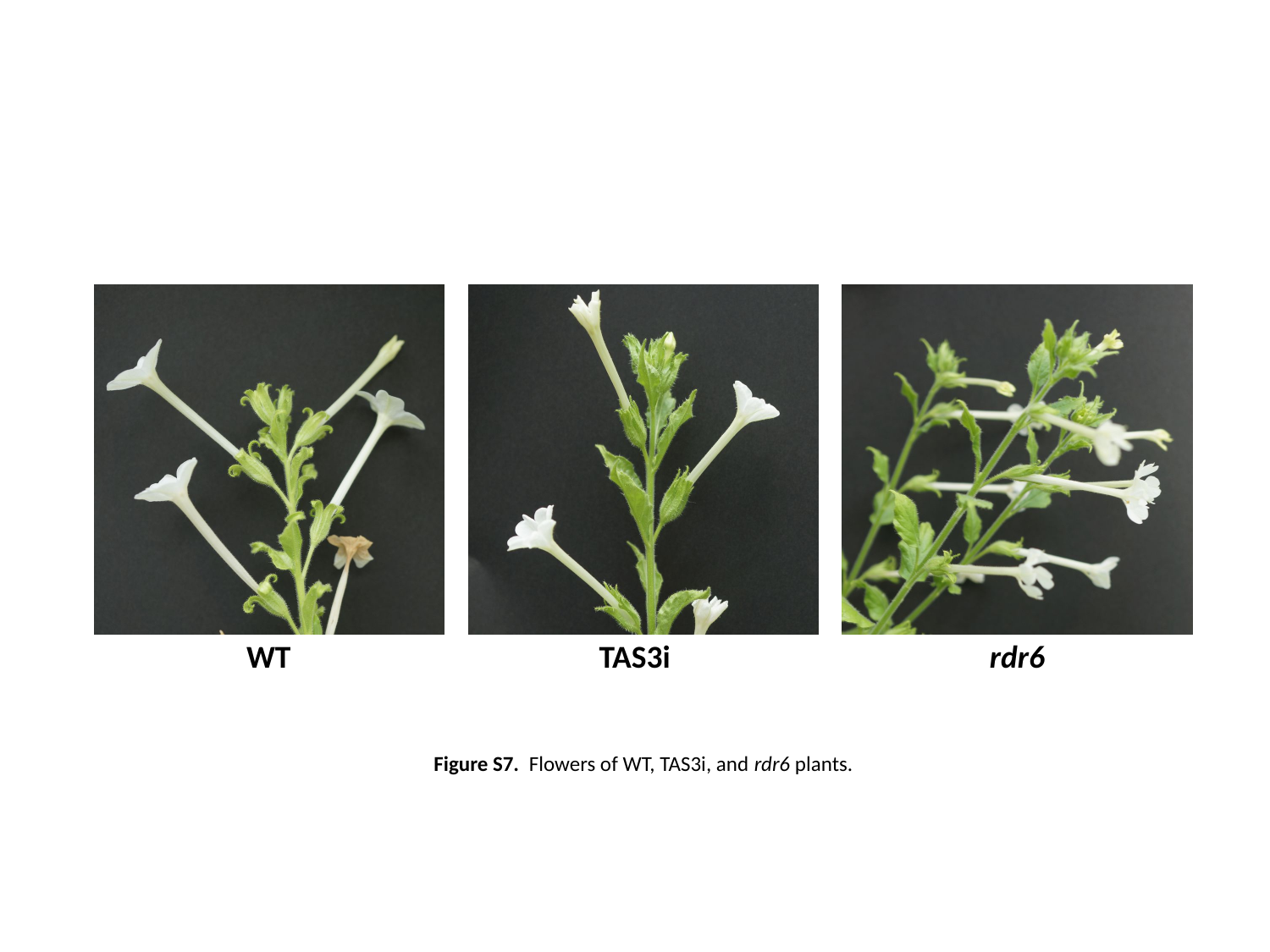

WT
TAS3i
rdr6
Figure S7. Flowers of WT, TAS3i, and rdr6 plants.

## Slide 8
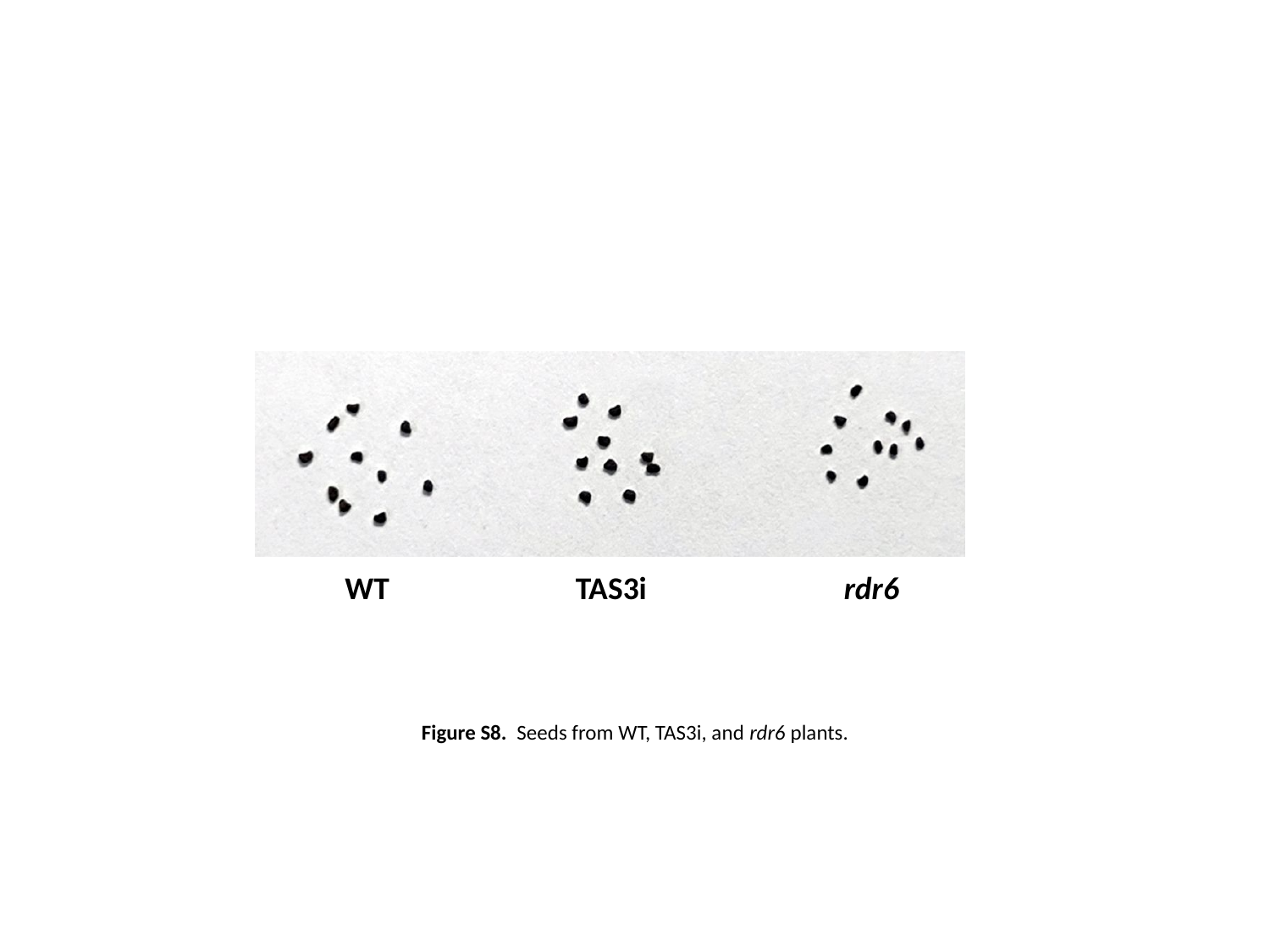

WT
TAS3i
rdr6
Figure S8. Seeds from WT, TAS3i, and rdr6 plants.
