# Supplementary material for: Recovery of RNA‐dependent RNA polymerase 6 gene‐knockout phenotypes in Nicotiana benthamiana via in vivo generation of inverted repeat construct of the trans‐acting short interference RNA3 sequence
Source: Plant J. 2025 Jul 18;123(2):e70350. doi: 10.1111/tpj.70350 (PMC12274081; doi:10.1111/tpj.70350)
Supplement: Supplementary file 2 — Table S1. Numbers of differentially expressed genes. Table S2. Differentially expressed genes in flowers of wild‐type plants versus rdr6 plants. Table S3. Differentially expressed genes in buds of wild‐type plants versus rdr6 plants. Table S4. GO analysis of upregulated genes in leaf samples (wild‐type vs. rdr6 plants). Table S5. GO analysis of downregulated genes in leaf samples (wild‐type vs. rdr6 plants). Table S6. GO analysis of upregulated genes in flower samples (wild‐type vs. rdr6 plants). Table S7. GO analysis of downregulated genes in flower samples (wild‐type vs. rdr6 plants). Table S8. GO analysis of upregulated genes in bud samples (wild‐type vs. rdr6 plants). Table S9. GO analysis of downregulated genes in bud samples (wild‐type vs. rdr6 plants). Table S10. Top 10 gene ontology terms among differentially expressed genes† (wild‐type vs. rdr6). Table S11. Top 10 gene ontology terms among differentially expressed genes† (wild‐type vs. TAS3i plants). Table S12. Differentially expressed genes in leaves of wild‐type plants vs. TAS3i plants. Table S13. Differentially expressed genes in flowers of wild‐type plants vs. TAS3i plants. Table S14. Differentially expressed genes in buds of wild‐type plants vs. TAS3i plants. Table S15. GO analysis of upregulated genes in leaf samples (wild‐type vs. TAS3i plants). Table S16. GO analysis of downregulated genes in leaf samples (wild‐type vs. TAS3i plants). Table S17. GO analysis of upregulated genes in flower samples (wild‐type vs. TAS3i plants). Table S18. GO analysis of downregulated genes in flower samples (wild‐type vs. TAS3i plants). Table S19. GO analysis of upregulated genes in bud samples (wild‐type vs. TAS3i plants). Table S20. GO analysis of downregulated genes in bud samples (wild‐type vs. TAS3i plants). Table S21. Top 10 gene ontology terms among differentially expressed genes† (rdr6 vs. TAS3i plants). Table S22. Differentially expressed genes in leaves of rdr6 plants versus TAS3i plants. Table S23. Different [file TPJ-123-0-s002.docx]

Table S1. The number of differentially expressed genes

|  | Wild-type vs *rdr6* | | Wild-type vs TAS3i | | *rdr6* vs TAS3i | |
| --- | --- | --- | --- | --- | --- | --- |
|  | UP | DOWN | UP | DOWN | UP | DOWN |
| Leaves | 14 | 8 | 42 | 12 | 60 | 16 |
| Flowers | 24 | 51 | 10 | 53 | 46 | 26 |
| Buds | 104 | 67 | 61 | 126 | 19 | 96 |

Table S2. The differentially expressed genes in flowers of wild-type plants vs *rdr6* plants

|  |  |  |  | expression amounts in flowers of | |  |  |
| --- | --- | --- | --- | --- | --- | --- | --- |
|  | Gene_ID | Gene | Gbkey | wild type plants | rdr6 plants | m.value [log2(expression ratio)] | Product |
| 1 | rna-XM_016609581.1 | LOC107787955 | mRNA | 0 | 47 | 5.496 | heavy metal-associated isoprenylated plant protein 22-like |
| 2 | rna-XM_016655118.1 | LOC107827891 | mRNA | 36 | 380 | 3.341 | proteinase inhibitor I-B-like |
| 3 | rna-XM_016647624.1 | LOC107821202 | mRNA | 388 | 2951 | 2.868 | galactinol synthase 2 |
| 4 | rna-XM_016604873.1 | LOC107783847 | mRNA | 93 | 700 | 2.853 | galactinol synthase 2-like |
| 5 | rna-XM_016652590.1 | LOC107825700 | mRNA | 56 | 283 | 2.278 | serine carboxypeptidase-like |
| 6 | rna-XM_016582423.1 | LOC107763917 | mRNA | 28 | 138 | 2.242 | COBRA-like protein 6, transcript variant X3 |
| 7 | rna-XM_016582422.1 | LOC107763917 | mRNA | 28 | 138 | 2.242 | COBRA-like protein 6, transcript variant X2 |
| 8 | rna-XM_016582421.1 | LOC107763917 | mRNA | 28 | 138 | 2.242 | COBRA-like protein 6, transcript variant X1 |
| 9 | rna-XM_016653169.1 | LOC107826218 | mRNA | 194 | 913 | 2.176 | **auxin response factor 4-like** |
| 10 | rna-XM_016651218.1 | LOC107824458 | mRNA | 121 | 513 | 2.025 | serine carboxypeptidase-like |
| 11 | rna-XM_016606813.1 | LOC107785490 | mRNA | 280 | 1091 | 1.903 | **auxin response factor 4-like** |
| 12 | rna-XM_016609285.1 | LOC107787683 | mRNA | 100 | 323 | 1.633 | protein FAF-like, chloroplastic |
| 13 | rna-XM_016624615.1 | LOC107801304 | mRNA | 169 | 540 | 1.617 | bidirectional sugar transporter SWEET12-like |
| 14 | rna-XM_016625309.1 | LOC107801897 | mRNA | 226 | 715 | 1.603 | histone H3.3 |
| 15 | rna-XM_016659989.1 | LOC107832177 | mRNA | 351 | 913 | 1.320 | protein FAF-like, chloroplastic |
| 16 | rna-XM_016660375.1 | LOC107832511 | mRNA | 550 | 1368 | 1.256 | probable aspartic protease At2g35615 |
| 17 | rna-XR_001647814.1 | LOC107785172 | misc_RNA | 367 | 907 | 1.246 | **auxin response factor 3-like, transcript variant X1** |
| 18 | rna-XR_001647815.1 | LOC107785172 | misc_RNA | 367 | 907 | 1.246 | **auxin response factor 3-like, transcript variant X3** |
| 19 | rna-XM_016606410.1 | LOC107785172 | mRNA | 368 | 907 | 1.243 | **auxin response factor 3-like, transcript variant X2** |
| 20 | rna-XM_016623096.1 | LOC107799954 | mRNA | 368 | 891 | 1.217 | uncharacterized LOC107799954, transcript variant X2 |
| 21 | rna-XM_016623095.1 | LOC107799954 | mRNA | 368 | 891 | 1.217 | uncharacterized LOC107799954, transcript variant X1 |
| 22 | rna-XM_016627510.1 | LOC107803745 | mRNA | 745 | 1684 | 1.118 | **auxin response factor 3-like, transcript variant X2** |
| 23 | rna-XR_001652067.1 | LOC107803745 | misc_RNA | 745 | 1683 | 1.117 | **auxin response factor 3-like, transcript variant X1** |
| 24 | rna-XR_001652068.1 | LOC107803745 | misc_RNA | 745 | 1683 | 1.117 | **auxin response factor 3-like, transcript variant X3** |
| 1 | rna-XM_016637755.1 | LOC107812610 | mRNA | 3817 | 1994 | -0.996 | beta-galactosidase-like |
| 2 | rna-XM_016649545.1 | LOC107822960 | mRNA | 1616 | 825 | -1.029 | beta-D-xylosidase 1-like, transcript variant X2 |
| 3 | rna-XM_016649544.1 | LOC107822960 | mRNA | 1686 | 854 | -1.040 | beta-D-xylosidase 1-like, transcript variant X1 |
| 4 | rna-XM_016585496.1 | LOC107766677 | mRNA | 2748 | 1368 | -1.065 | secoisolariciresinol dehydrogenase-like |
| 5 | rna-XM_016644886.1 | LOC107818832 | mRNA | 3598 | 1759 | -1.091 | beta-D-xylosidase 1-like |
| 6 | rna-XM_016599069.1 | LOC107778767 | mRNA | 1193 | 581 | -1.097 | cytokinin riboside 5'-monophosphate phosphoribohydrolase LOG1-like, transcript variant X1 |
| 7 | rna-XM_016647571.1 | LOC107821152 | mRNA | 1713 | 828 | -1.108 | carbonic anhydrase 2-like, transcript variant X2 |
| 8 | rna-XM_016647564.1 | LOC107821152 | mRNA | 1733 | 837 | -1.109 | carbonic anhydrase 2-like, transcript variant X1 |
| 9 | rna-XM_016596694.1 | LOC107776772 | mRNA | 1407 | 679 | -1.110 | probable aminotransferase TAT2 |
| 10 | rna-XM_016630276.1 | LOC107806169 | mRNA | 1246 | 593 | -1.130 | probable aminotransferase TAT2, transcript variant X1 |
| 11 | rna-XM_016630277.1 | LOC107806169 | mRNA | 1246 | 593 | -1.130 | probable aminotransferase TAT2, transcript variant X2 |
| 12 | rna-XM_016658044.1 | LOC107830480 | mRNA | 1291 | 607 | -1.148 | cathepsin B-like |
| 13 | rna-XM_016628298.1 | LOC107804413 | mRNA | 1016 | 476 | -1.153 | heavy metal-associated isoprenylated plant protein 3-like |
| 14 | rna-XM_016646097.1 | LOC107819915 | mRNA | 2171 | 1009 | -1.164 | abscisic acid and environmental stress-inducible protein TAS14-like |
| 15 | rna-XM_016649299.1 | LOC107822731 | mRNA | 709 | 327 | -1.175 | protein EXORDIUM-like |
| 16 | rna-XM_016599645.1 | LOC107779248 | mRNA | 7832 | 3466 | -1.235 | senescence-specific cysteine protease SAG12-like |
| 17 | rna-XM_016607761.1 | LOC107786280 | mRNA | 5066 | 2224 | -1.247 | senescence-specific cysteine protease SAG12-like |
| 18 | rna-XM_016643669.1 | LOC107817799 | mRNA | 1252 | 548 | -1.251 | nicotianamine synthase-like |
| 19 | rna-XM_016642073.1 | LOC107816366 | mRNA | 936 | 384 | -1.344 | cysteine protease inhibitor 8-like |
| 20 | rna-XM_016617035.1 | LOC107794537 | mRNA | 629 | 248 | -1.402 | ammonium transporter 3 member 1-like |
| 21 | rna-XM_016596103.1 | LOC107776233 | mRNA | 590 | 229 | -1.424 | sulfate transporter 3.1-like |
| 22 | rna-XM_016639720.1 | LOC107814322 | mRNA | 589 | 227 | -1.434 | U-box domain-containing protein 4-like |
| 23 | gene-LOC107796435 | LOC107796435 | exon | 466 | 172 | -1.497 | (+)-neomenthol dehydrogenase-like |
| 24 | rna-XM_016609426.1 | LOC107787819 | mRNA | 359 | 128 | -1.547 | osmotin |
| 25 | rna-XM_016599660.1 | LOC107779266 | mRNA | 1532 | 513 | -1.637 | beta-galactosidase-like |
| 26 | rna-XM_016581381.1 | LOC107762980 | mRNA | 1090 | 354 | -1.681 | beta-galactosidase-like |
| 27 | rna-XM_016577916.1 | LOC107759913 | mRNA | 906 | 293 | -1.688 | 1-aminocyclopropane-1-carboxylate oxidase-like |
| 28 | rna-XM_016601774.1 | LOC107781126 | mRNA | 1448 | 460 | -1.713 | 1-aminocyclopropane-1-carboxylate oxidase |
| 29 | rna-XM_016619110.1 | LOC107796351 | mRNA | 917 | 285 | -1.745 | proteinase inhibitor I-B-like |
| 30 | rna-XM_016629374.1 | LOC107805342 | mRNA | 1546 | 475 | -1.761 | (+)-neomenthol dehydrogenase-like |
| 31 | rna-XM_016604538.1 | LOC107783555 | mRNA | 214 | 62 | -1.846 | beta-D-glucosyl crocetin beta-1,6-glucosyltransferase-like |
| 32 | rna-XM_016604537.1 | LOC107783553 | mRNA | 441 | 126 | -1.866 | cyanidin-3-O-glucoside 2-O-glucuronosyltransferase-like |
| 33 | rna-XM_016629138.1 | LOC107805144 | mRNA | 455 | 122 | -1.958 | pectin acetylesterase 8-like |
| 34 | rna-XM_016624663.1 | LOC107801355 | mRNA | 266 | 65 | -2.092 | protein NEN4-like |
| 35 | gene-LOC107777219 | LOC107777219 | exon | 166 | 40 | -2.112 | probable pectate lyase 18 |
| 36 | rna-XM_016653822.1 | LOC107826794 | mRNA | 138 | 33 | -2.123 | endochitinase B |
| 37 | rna-XM_016615641.1 | LOC107793308 | mRNA | 577 | 135 | -2.155 | pectinesterase 2-like |
| 38 | rna-XM_016609816.1 | LOC107788163 | mRNA | 129 | 30 | -2.163 | monothiol glutaredoxin-S2-like |
| 39 | rna-XM_016641812.1 | LOC107816122 | mRNA | 519 | 112 | -2.271 | proteinase inhibitor type-2 |
| 40 | rna-XM_016645417.1 | LOC107819314 | mRNA | 168 | 36 | -2.281 | uncharacterized protein At5g22580-like |
| 41 | rna-XM_016600920.1 | LOC107780391 | mRNA | 149 | 31 | -2.324 | endochitinase 3-like |
| 42 | rna-XM_016658347.1 | LOC107830713 | mRNA | 119 | 24 | -2.369 | laccase-14-like |
| 43 | rna-XM_016591618.1 | LOC107772133 | mRNA | 452 | 89 | -2.403 | wound-induced protein WIN1-like |
| 44 | rna-XM_016589414.1 | LOC107770142 | mRNA | 271 | 52 | -2.441 | pectinesterase 2-like |
| 45 | rna-XM_016623821.1 | LOC107800618 | mRNA | 115 | 22 | -2.445 | cytochrome P450 78A6-like |
| 46 | rna-XM_016650053.1 | LOC107823411 | mRNA | 559 | 97 | -2.586 | glucan endo-1,3-beta-glucosidase, basic vacuolar |
| 47 | rna-XM_016624436.1 | LOC107801151 | mRNA | 152 | 22 | -2.847 | glucan endo-1,3-beta-glucosidase, basic vacuolar |
| 48 | rna-XM_016614910.1 | LOC107792673 | mRNA | 135 | 18 | -2.966 | laccase-7-like |
| 49 | rna-XM_016635289.1 | LOC107810500 | mRNA | 78 | 10 | -3.022 | polyphenol oxidase, chloroplastic-like |
| 50 | rna-XM_016644720.1 | LOC107818683 | mRNA | 92 | 11 | -3.123 | laccase-7-like |
| 51 | rna-XM_016609419.1 | LOC107787812 | mRNA | 97 | 9 | -3.489 | osmotin-like protein |

Table S3. The differentially expressed genes in buds of wild-type plants vs *rdr6* plants

|  |  |  |  | expression amounts in buds of | |  |  |
| --- | --- | --- | --- | --- | --- | --- | --- |
|  | Gene_ID | Gene | Gbkey | wild type plants | rdr6 plants | m.value [log2(expression ratio)] | Product |
| 1 | rna-XM_016623749.1 | LOC107800558 | mRNA | 0 | 355 | 8.503 | glycine-rich cell wall structural protein-like |
| 2 | rna-XM_016605916.1 | LOC107784740 | mRNA | 1 | 234 | 7.902 | stamen-specific protein FIL1-like |
| 3 | rna-XM_016611858.1 | LOC107789975 | mRNA | 0 | 194 | 7.631 | endochitinase EP3-like |
| 4 | rna-XM_016602246.1 | LOC107781529 | mRNA | 0 | 146 | 7.221 | AT-hook motif nuclear-localized protein 16-like |
| 5 | rna-XM_016639596.1 | LOC107814221 | mRNA | 2 | 269 | 7.103 | protein 108-like |
| 6 | rna-XM_016637635.1 | LOC107812512 | mRNA | 1 | 123 | 6.974 | protein trichome birefringence-like 28 |
| 7 | rna-XM_016625802.1 | LOC107802330 | mRNA | 1 | 117 | 6.902 | histone H2B-like |
| 8 | rna-XM_016621411.1 | LOC107798423 | mRNA | 5 | 540 | 6.786 | protein LIM1-like |
| 9 | rna-XM_016609278.1 | LOC107787674 | mRNA | 4 | 360 | 6.523 | glycine-rich cell wall structural protein-like |
| 10 | rna-XM_016609793.1 | LOC107788133 | mRNA | 0 | 79 | 6.335 | AT-hook motif nuclear-localized protein 16-like |
| 11 | rna-XM_016639178.1 | LOC107813865 | mRNA | 0 | 77 | 6.298 | classical arabinogalactan protein 6-like |
| 12 | rna-XM_016606551.1 | LOC107785281 | mRNA | 1 | 68 | 6.119 | stress-induced protein KIN1-like |
| 13 | rna-XR_001645813.1 | LOC107775692 | ncRNA | 0 | 63 | 6.009 | uncharacterized LOC107775692 |
| 14 | rna-XM_016630823.1 | LOC107806631 | mRNA | 1 | 57 | 5.864 | pectate lyase-like, transcript variant X1 |
| 15 | rna-XM_016630824.1 | LOC107806631 | mRNA | 1 | 57 | 5.864 | pectate lyase-like, transcript variant X2 |
| 16 | rna-XM_016578652.1 | LOC107760582 | mRNA | 4 | 223 | 5.832 | WAT1-related protein At2g39510-like |
| 17 | rna-XM_016585282.1 | LOC107766486 | mRNA | 0 | 55 | 5.813 | V-type proton ATPase subunit E-like |
| 18 | rna-XM_016609596.1 | LOC107787967 | mRNA | 3 | 146 | 5.636 | patatin-like protein 2 |
| 19 | rna-XM_016641191.1 | LOC107815555 | mRNA | 0 | 48 | 5.617 | stress-induced protein KIN2-like |
| 20 | rna-XM_016659821.1 | LOC107832019 | mRNA | 6 | 261 | 5.475 | pectinesterase 4-like, transcript variant X1 |
| 21 | rna-XM_016659822.1 | LOC107832019 | mRNA | 6 | 261 | 5.475 | pectinesterase 4-like, transcript variant X2 |
| 22 | rna-XM_016655631.1 | LOC107828342 | mRNA | 0 | 43 | 5.458 | mucin-1-like |
| 23 | rna-XM_016615505.1 | LOC107793208 | mRNA | 0 | 41 | 5.389 | uncharacterized LOC107793208 |
| 24 | rna-XR_001657526.1 | LOC107827448 | ncRNA | 1 | 41 | 5.389 | uncharacterized LOC107827448 |
| 25 | rna-XM_016578483.1 | LOC107760438 | mRNA | 0 | 40 | 5.353 | transcription repressor MYB4-like |
| 26 | rna-XM_016611533.1 | LOC107789674 | mRNA | 8 | 289 | 5.206 | protein TAP1-like |
| 27 | rna-XM_016578957.1 | LOC107760849 | mRNA | 2 | 71 | 5.181 | WAT1-related protein At2g39510-like |
| 28 | rna-XM_016660275.1 | LOC107832427 | mRNA | 2 | 67 | 5.098 | external alternative NAD(P)H-ubiquinone oxidoreductase B4, mitochondrial-like |
| 29 | rna-XM_016612677.1 | LOC107790719 | mRNA | 2 | 64 | 5.032 | vicilin-like seed storage protein At2g28490 |
| 30 | rna-XM_016604121.1 | LOC107783148 | mRNA | 24 | 726 | 4.950 | ATPase 8, plasma membrane-type |
| 31 | rna-XM_016593809.1 | LOC107774312 | mRNA | 2 | 58 | 4.890 | vicilin-like seed storage protein At2g28490 |
| 32 | rna-XM_016660016.1 | LOC107832171 | mRNA | 11 | 311 | 4.853 | late embryogenesis abundant protein 1-like |
| 33 | rna-XM_016583818.1 | LOC107765199 | mRNA | 2 | 53 | 4.759 | probable methyltransferase PMT15, transcript variant X2 |
| 34 | rna-XM_016583817.1 | LOC107765199 | mRNA | 2 | 53 | 4.759 | probable methyltransferase PMT15, transcript variant X1 |
| 35 | rna-XM_016591162.1 | LOC107771719 | mRNA | 11 | 284 | 4.722 | type III polyketide synthase B-like |
| 36 | rna-XM_016592973.1 | LOC107773574 | mRNA | 29 | 734 | 4.693 | L-ascorbate oxidase homolog |
| 37 | rna-XM_016647509.1 | LOC107821107 | mRNA | 9 | 206 | 4.548 | late embryogenesis abundant protein 1-like |
| 38 | rna-XM_016628700.1 | LOC107804769 | mRNA | 23 | 522 | 4.536 | ATPase 9, plasma membrane-type |
| 39 | gene-LOC107796867 | LOC107796867 | exon | 6 | 130 | 4.469 | BURP domain protein RD22-like |
| 40 | rna-XM_016626815.1 | LOC107803179 | mRNA | 12 | 259 | 4.463 | type III polyketide synthase B-like |
| 41 | rna-XM_016612117.1 | LOC107790214 | mRNA | 14 | 289 | 4.399 | stress-induced protein KIN2-like |
| 42 | rna-XM_016580587.1 | LOC107762241 | mRNA | 22 | 430 | 4.320 | anther-specific protein LAT52-like |
| 43 | rna-XM_016644487.1 | LOC107818462 | mRNA | 4 | 77 | 4.298 | external alternative NAD(P)H-ubiquinone oxidoreductase B3, mitochondrial-like |
| 44 | rna-XM_016660639.1 | LOC107832765 | mRNA | 6 | 108 | 4.201 | pectate lyase-like |
| 45 | rna-XM_016640257.1 | LOC107814800 | mRNA | 21 | 373 | 4.182 | non-specific lipid-transfer protein 2 |
| 46 | rna-XM_016588294.1 | LOC107769110 | mRNA | 24 | 425 | 4.178 | anther-specific protein LAT52-like |
| 47 | rna-XM_016586702.1 | LOC107767635 | mRNA | 7 | 123 | 4.167 | subtilisin-like protease SBT2.5 |
| 48 | rna-XM_016582050.1 | LOC107763563 | mRNA | 50 | 659 | 3.752 | beta-galactosidase 13-like |
| 49 | rna-XM_016656209.1 | LOC107828833 | mRNA | 18 | 225 | 3.675 | glycine-rich cell wall structural protein-like |
| 50 | rna-XM_016596613.1 | LOC107776702 | mRNA | 5 | 60 | 3.617 | putative non-specific lipid-transfer protein 14 |
| 51 | rna-XM_016587895.1 | LOC107768747 | mRNA | 7 | 77 | 3.491 | pectinesterase-like |
| 52 | rna-XM_016604204.1 | LOC107783237 | mRNA | 26 | 255 | 3.325 | glucan endo-1,3-beta-glucosidase 8-like |
| 53 | rna-XM_016656108.1 | LOC107828740 | mRNA | 24 | 211 | 3.168 | LRR receptor-like serine/threonine-protein kinase EFR |
| 54 | rna-XM_016621785.1 | LOC107798752 | mRNA | 71 | 615 | 3.146 | glucan endo-1,3-beta-glucosidase 8-like |
| 55 | rna-XM_016593286.1 | LOC107773859 | mRNA | 60 | 487 | 3.052 | beta-fructofuranosidase, insoluble isoenzyme 1-like |
| 56 | rna-XM_016658653.1 | LOC107830954 | mRNA | 27 | 211 | 2.998 | type III polyketide synthase A-like |
| 57 | rna-XM_016607676.1 | LOC107786229 | mRNA | 21 | 157 | 2.934 | cystathionine beta-lyase, chloroplastic-like |
| 58 | rna-XM_016607668.1 | LOC107786220 | mRNA | 20 | 149 | 2.929 | major pollen allergen Ole e 6-like |
| 59 | rna-XM_016601951.1 | LOC107781266 | mRNA | 120 | 883 | 2.911 | type III polyketide synthase A-like |
| 60 | rna-XM_016578650.1 | LOC107760581 | mRNA | 13 | 95 | 2.901 | cystathionine beta-lyase, chloroplastic-like, transcript variant X1 |
| 61 | rna-XM_016578651.1 | LOC107760581 | mRNA | 13 | 95 | 2.901 | cystathionine beta-lyase, chloroplastic-like, transcript variant X2 |
| 62 | rna-XM_016648345.1 | LOC107821889 | mRNA | 11 | 80 | 2.894 | protein STRICTOSIDINE SYNTHASE-LIKE 13-like |
| 63 | rna-XM_016602699.1 | LOC107781884 | mRNA | 25 | 173 | 2.822 | pectinesterase-like |
| 64 | rna-XM_016647544.1 | LOC107821133 | mRNA | 38 | 262 | 2.817 | polygalacturonase-like |
| 65 | rna-XM_016587497.1 | LOC107768376 | mRNA | 178 | 1198 | 2.782 | pectinesterase-like |
| 66 | rna-XM_016626713.1 | LOC107803097 | mRNA | 17 | 110 | 2.725 | dihydroflavonol-4-reductase-like |
| 67 | gene-LOC107832625 | LOC107832625 | exon | 13 | 84 | 2.723 | putative germin-like protein 2-1 |
| 68 | rna-XM_016653577.1 | LOC107826589 | mRNA | 32 | 203 | 2.697 | two-component response regulator-like APRR1 |
| 69 | rna-XM_016582845.1 | LOC107764290 | mRNA | 93 | 572 | 2.652 | pectinesterase-like |
| 70 | rna-XM_016620104.1 | LOC107797232 | mRNA | 48 | 292 | 2.636 | dihydroflavonol-4-reductase |
| 71 | rna-XM_016595805.1 | LOC107776000 | mRNA | 17 | 99 | 2.573 | phosphoenolpyruvate carboxykinase [ATP]-like |
| 72 | rna-XM_016644811.1 | LOC107818761 | mRNA | 19 | 106 | 2.512 | myb-related protein 305-like |
| 73 | rna-XM_016627603.1 | LOC107803813 | mRNA | 225 | 1219 | 2.469 | 4-coumarate--CoA ligase-like 1 |
| 74 | rna-XM_016597780.1 | LOC107777693 | mRNA | 25 | 133 | 2.443 | purple acid phosphatase 17-like |
| 75 | rna-XM_016604982.1 | LOC107783954 | mRNA | 37 | 188 | 2.377 | uncharacterized LOC107783954 |
| 76 | gene-LOC107778965 | LOC107778965 | exon | 31 | 155 | 2.353 | pectin acetylesterase 8-like |
| 77 | rna-XM_016581596.1 | LOC107763147 | mRNA | 262 | 1246 | 2.281 | sugar transport protein 8-like |
| 78 | rna-XM_016653236.1 | LOC107826272 | mRNA | 33 | 155 | 2.263 | la-related protein 6C-like |
| 79 | rna-XM_016639446.1 | LOC107814113 | mRNA | 161 | 744 | 2.240 | 4-coumarate--CoA ligase-like 1 |
| 80 | rna-XM_016608743.1 | LOC107787207 | mRNA | 753 | 3188 | 2.113 | sugar transport protein 8-like |
| 81 | rna-XM_016579984.1 | LOC107761721 | mRNA | 88 | 363 | 2.076 | protein STRICTOSIDINE SYNTHASE-LIKE 13-like |
| 82 | rna-XM_016591148.1 | LOC107771705 | mRNA | 75 | 295 | 2.007 | snakin-2-like |
| 83 | rna-XM_016596165.1 | LOC107776287 | mRNA | 87 | 342 | 2.006 | glyceraldehyde-3-phosphate dehydrogenase, cytosolic |
| 84 | rna-XM_016632387.1 | LOC107807930 | mRNA | 51 | 199 | 1.996 | lysine histidine transporter-like 5 |
| 85 | gene-LOC107777646 | LOC107777646 | exon | 45 | 167 | 1.923 | desiccation-related protein PCC13-62-like |
| 86 | rna-XM_016640326.1 | LOC107814848 | mRNA | 1545 | 5715 | 1.919 | L-ascorbate oxidase homolog |
| 87 | rna-XM_016585778.1 | LOC107766889 | mRNA | 238 | 797 | 1.775 | L-ascorbate oxidase homolog |
| 88 | rna-XM_016581465.1 | LOC107763048 | mRNA | 189 | 628 | 1.764 | tetraketide alpha-pyrone reductase 2-like |
| 89 | rna-XM_016582909.1 | LOC107764355 | mRNA | 70 | 227 | 1.729 | probable pectate lyase 18 |
| 90 | rna-XM_016622136.1 | LOC107799065 | mRNA | 75 | 242 | 1.722 | pectin acetylesterase 7-like |
| 91 | rna-XM_016607296.1 | LOC107785898 | mRNA | 108 | 345 | 1.707 | ADP,ATP carrier protein 1, mitochondrial-like, transcript variant X2 |
| 92 | rna-XM_016607294.1 | LOC107785898 | mRNA | 108 | 345 | 1.707 | ADP,ATP carrier protein 1, mitochondrial-like, transcript variant X1 |
| 93 | rna-XM_016636216.1 | LOC107811316 | mRNA | 78 | 246 | 1.689 | desiccation-related protein PCC13-62-like |
| 94 | rna-XM_016607696.1 | LOC107786249 | mRNA | 110 | 328 | 1.608 | caffeic acid 3-O-methyltransferase-like |
| 95 | rna-XM_016580091.1 | LOC107761808 | mRNA | 429 | 1211 | 1.529 | uncharacterized LOC107761808 |
| 96 | rna-XM_016606813.1 | LOC107785490 | mRNA | 973 | 2598 | 1.448 | **auxin response factor 4-like** |
| 97 | rna-XM_016606355.1 | LOC107785130 | mRNA | 286 | 753 | 1.428 | uncharacterized LOC107785130 |
| 98 | rna-XM_016610823.1 | LOC107789055 | mRNA | 138 | 357 | 1.403 | laccase-11-like |
| 99 | rna-XM_016653169.1 | LOC107826218 | mRNA | 815 | 2107 | 1.402 | **auxin response factor 4-like** |
| 100 | rna-XM_016624886.1 | LOC107801540 | mRNA | 187 | 461 | 1.333 | chaperone protein dnaJ 20, chloroplastic-like |
| 101 | rna-XM_016620041.1 | LOC107797177 | mRNA | 359 | 848 | 1.272 | sugar transport protein 10-like |
| 102 | rna-XM_016642899.1 | LOC107817126 | mRNA | 1901 | 4398 | 1.242 | anther-specific protein LAT52-like |
| 103 | rna-XM_016638610.1 | LOC107813352 | mRNA | 230 | 528 | 1.230 | non-classical arabinogalactan protein 31-like |
| 104 | rna-XM_016641252.1 | LOC107815637 | mRNA | 3336 | 6421 | 0.976 | asparagine synthetase [glutamine-hydrolyzing]-like |
| 1 | rna-XM_016656244.1 | LOC107828858 | mRNA | 6196 | 3123 | -0.957 | protein 108-like |
| 2 | rna-XM_016650109.1 | LOC107823443 | mRNA | 5568 | 2799 | -0.961 | protein 108-like |
| 3 | rna-XM_016640422.1 | LOC107814918 | mRNA | 2856 | 1416 | -0.981 | dehydrodolichyl diphosphate synthase 6-like |
| 4 | rna-XM_016578217.1 | LOC107760202 | mRNA | 4472 | 2203 | -0.990 | putative UDP-rhamnose:rhamnosyltransferase 1 |
| 5 | rna-XM_016654158.1 | LOC107827098 | mRNA | 2718 | 1299 | -1.034 | probable aquaporin NIP7-1 |
| 6 | rna-XM_016622413.1 | LOC107799313 | mRNA | 3704 | 1726 | -1.070 | thaumatin-like protein |
| 7 | rna-XM_016637879.1 | LOC107812723 | mRNA | 2065 | 955 | -1.081 | 7-deoxyloganetic acid glucosyltransferase-like |
| 8 | rna-XM_016659974.1 | LOC107832164 | mRNA | 2165 | 1000 | -1.083 | transcription factor ABORTED MICROSPORES-like, transcript variant X4 |
| 9 | rna-XM_016659971.1 | LOC107832164 | mRNA | 2165 | 1000 | -1.083 | transcription factor ABORTED MICROSPORES-like, transcript variant X1 |
| 10 | rna-XM_016659972.1 | LOC107832164 | mRNA | 2165 | 1000 | -1.083 | transcription factor ABORTED MICROSPORES-like, transcript variant X2 |
| 11 | rna-XM_016659976.1 | LOC107832164 | mRNA | 2165 | 998 | -1.086 | transcription factor ABORTED MICROSPORES-like, transcript variant X5 |
| 12 | rna-XM_016659973.1 | LOC107832164 | mRNA | 2165 | 998 | -1.086 | transcription factor ABORTED MICROSPORES-like, transcript variant X3 |
| 13 | rna-XM_016603966.1 | LOC107783011 | mRNA | 1416 | 638 | -1.119 | beta-glucosidase BoGH3B-like |
| 14 | rna-XM_016641160.1 | LOC107815552 | mRNA | 2432 | 1083 | -1.136 | probable aquaporin NIP7-1 |
| 15 | rna-XM_016606260.1 | LOC107785040 | mRNA | 1062 | 463 | -1.166 | ribulose bisphosphate carboxylase small chain S41, chloroplastic-like |
| 16 | rna-XM_016628326.1 | LOC107804431 | mRNA | 4879 | 2117 | -1.173 | ervatamin-B-like |
| 17 | rna-XM_016645620.1 | LOC107819508 | mRNA | 1104 | 475 | -1.185 | beta-amyrin 28-oxidase-like |
| 18 | gene-LOC107800256 | LOC107800256 | exon | 1384 | 587 | -1.206 | phenylalanine ammonia-lyase G4-like |
| 19 | rna-XM_016607774.1 | LOC107786313 | mRNA | 805 | 340 | -1.212 | non-specific lipid-transfer protein 13-like |
| 20 | rna-XM_016585396.1 | LOC107766587 | mRNA | 976 | 412 | -1.213 | sugar transport protein 14-like |
| 21 | rna-XM_016590154.1 | LOC107770822 | mRNA | 790 | 333 | -1.215 | L-ascorbate oxidase homolog |
| 22 | rna-XM_016657212.1 | LOC107829716 | mRNA | 1509 | 627 | -1.235 | probable beta-1,3-galactosyltransferase 8, transcript variant X5 |
| 23 | rna-XM_016657198.1 | LOC107829716 | mRNA | 1510 | 626 | -1.239 | probable beta-1,3-galactosyltransferase 8, transcript variant X3 |
| 24 | rna-XR_001654083.1 | LOC107812173 | ncRNA | 11434 | 4740 | -1.239 | uncharacterized LOC107812173 |
| 25 | rna-XM_016657191.1 | LOC107829716 | mRNA | 1513 | 626 | -1.242 | probable beta-1,3-galactosyltransferase 8, transcript variant X2 |
| 26 | rna-XM_016657206.1 | LOC107829716 | mRNA | 1519 | 627 | -1.245 | probable beta-1,3-galactosyltransferase 8, transcript variant X4 |
| 27 | rna-XM_016657184.1 | LOC107829716 | mRNA | 1517 | 626 | -1.245 | probable beta-1,3-galactosyltransferase 8, transcript variant X1 |
| 28 | rna-XM_016590153.1 | LOC107770821 | mRNA | 792 | 325 | -1.253 | L-ascorbate oxidase homolog |
| 29 | rna-XM_016622107.1 | LOC107799043 | mRNA | 688 | 281 | -1.260 | heparanase-like protein 2 |
| 30 | rna-XM_016592081.1 | LOC107772583 | mRNA | 452 | 178 | -1.313 | subtilisin-like protease SBT1.5 |
| 31 | rna-XM_016604064.1 | LOC107783099 | mRNA | 2149 | 845 | -1.315 | UDP-glycosyltransferase 83A1-like |
| 32 | rna-XM_016654250.1 | LOC107827166 | mRNA | 4025 | 1579 | -1.318 | spermidine hydroxycinnamoyl transferase-like, transcript variant X2 |
| 33 | rna-XM_016654249.1 | LOC107827166 | mRNA | 4025 | 1579 | -1.318 | spermidine hydroxycinnamoyl transferase-like, transcript variant X1 |
| 34 | rna-XM_016580156.1 | LOC107761866 | mRNA | 473 | 182 | -1.346 | WSC domain-containing protein ARB_07867-like |
| 35 | rna-XM_016626629.1 | LOC107803031 | mRNA | 463 | 177 | -1.356 | GDSL esterase/lipase EXL1-like, transcript variant X4 |
| 36 | rna-XM_016605912.1 | LOC107784736 | mRNA | 1220 | 463 | -1.366 | O-acyltransferase WSD1-like |
| 37 | rna-XM_016610227.1 | LOC107788550 | mRNA | 1513 | 572 | -1.372 | spermidine hydroxycinnamoyl transferase-like |
| 38 | rna-XM_016626626.1 | LOC107803031 | mRNA | 666 | 251 | -1.376 | GDSL esterase/lipase EXL1-like, transcript variant X1 |
| 39 | rna-XM_016656094.1 | LOC107828729 | mRNA | 1868 | 703 | -1.378 | O-acyltransferase WSD1-like |
| 40 | rna-XM_016626628.1 | LOC107803031 | mRNA | 666 | 250 | -1.382 | GDSL esterase/lipase EXL1-like, transcript variant X3 |
| 41 | rna-XM_016619076.1 | LOC107796325 | mRNA | 534 | 198 | -1.400 | proline-rich extensin-like protein EPR1 |
| 42 | rna-XM_016626627.1 | LOC107803031 | mRNA | 609 | 224 | -1.411 | GDSL esterase/lipase EXL1-like, transcript variant X2 |
| 43 | rna-XM_016658955.1 | LOC107831213 | mRNA | 1201 | 434 | -1.437 | phenylalanine ammonia-lyase G4-like |
| 44 | rna-XM_016632980.1 | LOC107808450 | mRNA | 1469 | 523 | -1.458 | non-specific lipid-transfer protein 13-like, transcript variant X3 |
| 45 | rna-XM_016632979.1 | LOC107808450 | mRNA | 1470 | 523 | -1.459 | non-specific lipid-transfer protein 13-like, transcript variant X2 |
| 46 | rna-XM_016632978.1 | LOC107808450 | mRNA | 1470 | 523 | -1.459 | non-specific lipid-transfer protein 13-like, transcript variant X1 |
| 47 | rna-XM_016584882.1 | LOC107766138 | mRNA | 317 | 112 | -1.469 | sugar carrier protein C-like |
| 48 | rna-XM_016650869.1 | LOC107824131 | mRNA | 2311 | 816 | -1.470 | beta-xylosidase/alpha-L-arabinofuranosidase 1-like |
| 49 | rna-XM_016628753.1 | LOC107804817 | mRNA | 686 | 242 | -1.472 | proline-rich extensin-like protein EPR1 |
| 50 | rna-XM_016648613.1 | LOC107822110 | mRNA | 695 | 245 | -1.473 | beta-galactosidase 15-like |
| 51 | rna-XM_016586939.1 | LOC107767841 | mRNA | 593 | 209 | -1.473 | probable pectinesterase 68 |
| 52 | rna-XM_016584553.1 | LOC107765852 | mRNA | 369 | 128 | -1.496 | GDSL esterase/lipase EXL3-like |
| 53 | rna-XM_016630367.1 | LOC107806241 | mRNA | 2188 | 758 | -1.498 | beta-galactosidase 15-like |
| 54 | rna-XR_001653136.1 | LOC107808450 | misc_RNA | 1176 | 406 | -1.503 | non-specific lipid-transfer protein 13-like, transcript variant X4 |
| 55 | rna-XM_016625886.1 | LOC107802398 | mRNA | 315 | 105 | -1.553 | uncharacterized LOC107802398, transcript variant X2 |
| 56 | rna-XM_016639985.1 | LOC107814550 | mRNA | 596 | 198 | -1.558 | 60S acidic ribosomal protein P1-like |
| 57 | rna-XM_016625887.1 | LOC107802398 | mRNA | 315 | 104 | -1.567 | uncharacterized LOC107802398, transcript variant X3 |
| 58 | rna-XM_016625885.1 | LOC107802398 | mRNA | 315 | 104 | -1.567 | uncharacterized LOC107802398, transcript variant X1 |
| 59 | rna-XM_016637959.1 | LOC107812789 | mRNA | 424 | 137 | -1.598 | phospholipase D delta-like |
| 60 | rna-XM_016628192.1 | LOC107804318 | mRNA | 804 | 242 | -1.701 | probable beta-D-xylosidase 5 |
| 61 | rna-XM_016600920.1 | LOC107780391 | mRNA | 449 | 133 | -1.724 | endochitinase 3-like |
| 62 | rna-XM_016628947.1 | LOC107804979 | mRNA | 258 | 76 | -1.732 | sugar transport protein 12-like |
| 63 | rna-XM_016636364.1 | LOC107811438 | mRNA | 1281 | 377 | -1.733 | phospholipase D delta-like |
| 64 | rna-XM_016605567.1 | LOC107784434 | mRNA | 396 | 110 | -1.816 | endochitinase 3 |
| 65 | rna-XM_016659495.1 | LOC107831707 | mRNA | 411 | 107 | -1.910 | 21 kDa protein-like |
| 66 | rna-XM_016649224.1 | LOC107822668 | mRNA | 658 | 171 | -1.913 | uncharacterized LOC107822668 |
| 67 | rna-XM_016648351.1 | LOC107821894 | mRNA | 332 | 80 | -2.022 | uncharacterized LOC107821894 |

Table S4. The GO analysis of up-regulated genes in leaves (wild-type vs *rdr6* plants)

| No | GO_term | GOid | All | DEG | P-value | Ontologies |
| --- | --- | --- | --- | --- | --- | --- |
| 1 | **auxin-activated signaling pathway** | GO:0009734 | 326 | 4 | 0.000247 | biological_process |
| 2 | vegetative phase change | GO:0010050 | 8 | 2 | 0.001128 | biological_process |
| 3 | abaxial cell fate specification | GO:0010158 | 10 | 2 | 0.001128 | biological_process |
| 4 | negative regulation of endopeptidase activity | GO:0010951 | 174 | 3 | 0.001178 | biological_process |
| 5 | serine-type endopeptidase inhibitor activity | GO:0004867 | 44 | 2 | 0.010584 | molecular_function |
| 6 | glutathione transferase activity | GO:0004364 | 100 | 2 | 0.04373 | molecular_function |
| 7 | response to hormone | GO:0009725 | 148 | 2 | 0.08105 | biological_process |

Table S5. The GO analysis of down-regulated genes in leaves (wild-type vs *rdr6* plants)*

| No | GO_term | GOid | All | DEG | P-value | Ontologies |
| --- | --- | --- | --- | --- | --- | --- |
| 1 | transferase activity | GO:0016740 | 4549 | 3 | 1 | molecular_function |
| 2 | transferase activity, transferring acyl groups | GO:0016746 | 440 | 2 | 1 | molecular_function |
| 3 | nucleotide binding | GO:0000166 | 6085 | 2 | 1 | molecular_function |
| 4 | transcription, RNA-templated | GO:0001172 | 18 | 1 | 1 | biological_process |
| 5 | RNA-directed RNA polymerase activity | GO:0003968 | 18 | 1 | 1 | molecular_function |
| 6 | methionine adenosyltransferase activity | GO:0004478 | 19 | 1 | 1 | molecular_function |
| 7 | S-adenosylmethionine biosynthetic process | GO:0006556 | 19 | 1 | 1 | biological_process |
| 8 | secondary active sulfate transmembrane transporter activity | GO:0008271 | 61 | 1 | 1 | molecular_function |
| 9 | sulfate transport | GO:0008272 | 61 | 1 | 1 | biological_process |
| 10 | sulfate transmembrane transporter activity | GO:0015116 | 61 | 1 | 1 | molecular_function |
| 11 | sulfate transmembrane transport | GO:1902358 | 61 | 1 | 1 | biological_process |
| 12 | one-carbon metabolic process | GO:0006730 | 96 | 1 | 1 | biological_process |
| 13 | cellular_component | GO:0005575 | 219 | 1 | 1 | cellular_component |
| 14 | integral component of plasma membrane | GO:0005887 | 224 | 1 | 1 | cellular_component |
| 15 | transmembrane transport | GO:0055085 | 2027 | 1 | 1 | biological_process |
| 16 | biological_process | GO:0008150 | 2045 | 1 | 1 | biological_process |
| 17 | transport | GO:0006810 | 2879 | 1 | 1 | biological_process |
| 18 | cytoplasm | GO:0005737 | 3451 | 1 | 1 | cellular_component |
| 19 | oxidoreductase activity | GO:0016491 | 3755 | 1 | 1 | molecular_function |
| 20 | oxidation-reduction process | GO:0055114 | 4882 | 1 | 1 | biological_process |
| 21 | metal ion binding | GO:0046872 | 7430 | 1 | 1 | molecular_function |
| 22 | ATP binding | GO:0005524 | 8057 | 1 | 1 | molecular_function |
| 23 | nucleic acid binding | GO:0003676 | 10114 | 1 | 1 | molecular_function |
| 24 | integral component of membrane | GO:0016021 | 22126 | 1 | 1 | cellular_component |
| 25 | membrane | GO:0016020 | 22913 | 1 | 1 | cellular_component |

*There were no GO terms with P-values less than 1.

Table S6. The GO analysis of up-regulated genes in flowers (wild-type vs *rdr6* plants)

| No | GO_term | GOid | All | DEG | P-value | Ontologies |
| --- | --- | --- | --- | --- | --- | --- |
| 1 | vegetative phase change | GO:0010050 | 8 | 6 | 5.95E-14 | biological_process |
| 2 | abaxial cell fate specification | GO:0010158 | 10 | 6 | 7.93E-14 | biological_process |
| 3 | response to hormone | GO:0009725 | 148 | 8 | 7.06E-12 | biological_process |
| 4 | **auxin-activated signaling pathway** | GO:0009734 | 326 | 8 | 2.42E-09 | biological_process |
| 5 | floral meristem determinacy | GO:0010582 | 22 | 4 | 3.70E-07 | biological_process |
| 6 | anchored component of membrane | GO:0031225 | 48 | 3 | 0.001056 | cellular_component |
| 7 | cellulose microfibril organization | GO:0010215 | 60 | 3 | 0.00172 | biological_process |
| 8 | cell growth | GO:0016049 | 84 | 3 | 0.003989 | biological_process |
| 9 | alpha-galactosidase activity | GO:0004557 | 15 | 2 | 0.010289 | molecular_function |
| 10 | raffinose alpha-galactosidase activity | GO:0052692 | 15 | 2 | 0.010289 | molecular_function |
| 11 | transcription, DNA-templated | GO:0006351 | 2881 | 8 | 0.012337 | biological_process |
| 12 | regulation of transcription, DNA-templated | GO:0006355 | 4728 | 8 | 0.29866 | biological_process |
| 13 | carboxypeptidase activity | GO:0004180 | 101 | 2 | 0.29866 | molecular_function |
| 14 | serine-type carboxypeptidase activity | GO:0004185 | 108 | 2 | 0.3159 | molecular_function |
| 15 | DNA binding | GO:0003677 | 6192 | 9 | 0.34748 | molecular_function |

Table S7. The GO analysis of down-regulated genes in flowers (wild-type vs *rdr6* plants)

| No | GO_term | GOid | All | DEG | P-value | Ontologies |
| --- | --- | --- | --- | --- | --- | --- |
| 1 | carbohydrate metabolic process | GO:0005975 | 1391 | 10 | 0.000279 | biological_process |
| 2 | apoplast | GO:0048046 | 279 | 6 | 0.000279 | cellular_component |
| 3 | seed coat development | GO:0010214 | 10 | 3 | 0.000279 | biological_process |
| 4 | hydrolase activity, hydrolyzing O-glycosyl compounds | GO:0004553 | 869 | 8 | 0.000538 | molecular_function |
| 5 | vacuole | GO:0005773 | 384 | 6 | 0.000538 | cellular_component |
| 6 | alpha-L-arabinofuranosidase activity | GO:0046556 | 19 | 3 | 0.000538 | molecular_function |
| 7 | hydrolase activity, acting on glycosyl bonds | GO:0016798 | 739 | 7 | 0.001356 | molecular_function |
| 8 | chitin binding | GO:0008061 | 30 | 3 | 0.001356 | molecular_function |
| 9 | hydrolase activity | GO:0016787 | 5182 | 16 | 0.001835 | molecular_function |
| 10 | 1-aminocyclopropane-1-carboxylate oxidase activity | GO:0009815 | 3 | 2 | 0.002646 | molecular_function |
| 11 | plant-type cell wall | GO:0009505 | 206 | 4 | 0.008905 | cellular_component |
| 12 | beta-galactosidase activity | GO:0004565 | 66 | 3 | 0.008905 | molecular_function |
| 13 | lignin catabolic process | GO:0046274 | 72 | 3 | 0.009361 | biological_process |
| 14 | hydroquinone:oxygen oxidoreductase activity | GO:0052716 | 72 | 3 | 0.009361 | molecular_function |
| 15 | ethylene biosynthetic process | GO:0009693 | 11 | 2 | 0.013701 | biological_process |
| 16 | cellular amino acid metabolic process | GO:0006520 | 128 | 3 | 0.043021 | biological_process |
| 17 | metabolic process | GO:0008152 | 3072 | 10 | 0.043265 | biological_process |
| 18 | cell wall macromolecule catabolic process | GO:0016998 | 25 | 2 | 0.05101 | biological_process |
| 19 | response to biotic stimulus | GO:0009607 | 158 | 3 | 0.06308 | biological_process |
| 20 | carbon utilization | GO:0015976 | 29 | 2 | 0.06308 | biological_process |
| 21 | defense response | GO:0006952 | 1665 | 7 | 0.07564 | biological_process |
| 22 | negative regulation of endopeptidase activity | GO:0010951 | 174 | 3 | 0.07564 | biological_process |
| 23 | transaminase activity | GO:0008483 | 194 | 3 | 0.0988 | molecular_function |
| 24 | serine-type endopeptidase inhibitor activity | GO:0004867 | 44 | 2 | 0.11174 | molecular_function |
| 25 | L-ascorbic acid binding | GO:0031418 | 48 | 2 | 0.1267 | molecular_function |
| 26 | oxidoreductase activity, oxidizing metal ions | GO:0016722 | 49 | 2 | 0.12674 | molecular_function |
| 27 | carbonate dehydratase activity | GO:0004089 | 56 | 2 | 0.15767 | molecular_function |
| 28 | cell wall | GO:0005618 | 559 | 4 | 0.162 | cellular_component |
| 29 | chitin catabolic process | GO:0006032 | 59 | 2 | 0.162 | biological_process |
| 30 | chitinase activity | GO:0004568 | 60 | 2 | 0.162 | molecular_function |
| 31 | copper ion binding | GO:0005507 | 314 | 3 | 0.28707 | molecular_function |
| 32 | polysaccharide catabolic process | GO:0000272 | 88 | 2 | 0.31713 | biological_process |
| 33 | oxidoreductase activity | GO:0016491 | 3755 | 9 | 0.38436 | molecular_function |
| 34 | pyridoxal phosphate binding | GO:0030170 | 387 | 3 | 0.45522 | molecular_function |
| 35 | nicotianamine synthase activity | GO:0030410 | 4 | 1 | 0.46669 | molecular_function |
| 36 | nicotianamine biosynthetic process | GO:0030418 | 4 | 1 | 0.46669 | biological_process |
| 37 | biosynthetic process | GO:0009058 | 415 | 3 | 0.50843 | biological_process |
| 38 | oxidation-reduction process | GO:0055114 | 4882 | 10 | 0.58179 | biological_process |
| 39 | response to water | GO:0009415 | 6 | 1 | 0.6039 | biological_process |
| 40 | nucleic acid binding | GO:0003676 | 10114 | 1 | 0.62989 | molecular_function |
| 41 | membrane | GO:0016020 | 22913 | 7 | 0.65686 | cellular_component |
| 42 | cell wall modification | GO:0042545 | 152 | 2 | 0.65686 | biological_process |
| 43 | aspartyl esterase activity | GO:0045330 | 152 | 2 | 0.65686 | molecular_function |
| 44 | pectinesterase activity | GO:0030599 | 154 | 2 | 0.65845 | molecular_function |
| 45 | integral component of membrane | GO:0016021 | 22126 | 7 | 0.79013 | cellular_component |
| 46 | glucan endo-1,3-beta-D-glucosidase activity | GO:0042973 | 12 | 1 | 0.95231 | molecular_function |
| 47 | enzyme inhibitor activity | GO:0004857 | 195 | 2 | 0.9656 | molecular_function |

Table S8. The GO analysis of up-regulated genes in buds (wild-type vs *rdr6* plants)

| No | GO_term | GOid | All | DEG | P-value | Ontologies |
| --- | --- | --- | --- | --- | --- | --- |
| 1 | pectin catabolic process | GO:0045490 | 212 | 10 | 2.68E-09 | biological_process |
| 2 | pollen exine formation | GO:0010584 | 19 | 4 | 5.36E-05 | biological_process |
| 3 | cell wall modification | GO:0042545 | 152 | 6 | 7.23E-05 | biological_process |
| 4 | aspartyl esterase activity | GO:0045330 | 152 | 6 | 7.23E-05 | molecular_function |
| 5 | pectinesterase activity | GO:0030599 | 154 | 6 | 7.23E-05 | molecular_function |
| 6 | extracellular space | GO:0005615 | 90 | 5 | 0.000104 | cellular_component |
| 7 | cystathionine beta-lyase activity | GO:0004121 | 6 | 3 | 0.000104 | molecular_function |
| 8 | 'de novo' L-methionine biosynthetic process | GO:0071266 | 6 | 3 | 0.000104 | biological_process |
| 9 | enzyme inhibitor activity | GO:0004857 | 195 | 6 | 0.000154 | molecular_function |
| 10 | sporopollenin biosynthetic process | GO:0080110 | 9 | 3 | 0.00021 | biological_process |
| 11 | cell wall | GO:0005618 | 559 | 8 | 0.0004 | cellular_component |
| 12 | negative regulation of catalytic activity | GO:0043086 | 259 | 6 | 0.000532 | biological_process |
| 13 | pectate lyase activity | GO:0030570 | 58 | 4 | 0.000532 | molecular_function |
| 14 | negative regulation of transposition | GO:0010529 | 2 | 2 | 0.002803 | biological_process |
| 15 | polyketide biosynthetic process | GO:0030639 | 2 | 2 | 0.002803 | biological_process |
| 16 | tetraketide alpha-pyrone synthase activity | GO:0090439 | 2 | 2 | 0.002803 | molecular_function |
| 17 | biosynthetic process | GO:0009058 | 415 | 6 | 0.005642 | biological_process |
| 18 | chromatin DNA binding | GO:0031490 | 6 | 2 | 0.011587 | molecular_function |
| 19 | nucleic acid binding | GO:0003676 | 10114 | 1 | 0.012846 | molecular_function |
| 20 | pollen wall assembly | GO:0010208 | 7 | 2 | 0.013397 | biological_process |
| 21 | vegetative phase change | GO:0010050 | 8 | 2 | 0.01521 | biological_process |
| 22 | positive regulation of histone H3-K9 dimethylation | GO:1900111 | 8 | 2 | 0.01521 | biological_process |
| 23 | transmembrane transporter activity | GO:0022857 | 355 | 5 | 0.021275 | molecular_function |
| 24 | abaxial cell fate specification | GO:0010158 | 10 | 2 | 0.021275 | biological_process |
| 25 | negative regulation of histone acetylation | GO:0035067 | 12 | 2 | 0.025938 | biological_process |
| 26 | hydrolase activity | GO:0016787 | 5182 | 18 | 0.03196 | molecular_function |
| 27 | extracellular region | GO:0005576 | 909 | 7 | 0.034665 | cellular_component |
| 28 | extrinsic component of mitochondrial inner membrane | GO:0031314 | 15 | 2 | 0.034665 | cellular_component |
| 29 | strictosidine synthase activity | GO:0016844 | 21 | 2 | 0.06202 | molecular_function |
| 30 | coenzyme binding | GO:0050662 | 118 | 3 | 0.07987 | molecular_function |
| 31 | transferase activity, transferring acyl groups other than amino-acyl groups | GO:0016747 | 306 | 4 | 0.10667 | molecular_function |
| 32 | hydrogen-exporting ATPase activity, phosphorylative mechanism | GO:0008553 | 30 | 2 | 0.10968 | molecular_function |
| 33 | copper ion binding | GO:0005507 | 314 | 4 | 0.11033 | molecular_function |
| 34 | pollen development | GO:0009555 | 157 | 3 | 0.15854 | biological_process |
| 35 | substrate-specific transmembrane transporter activity | GO:0022891 | 169 | 3 | 0.18468 | molecular_function |
| 36 | ATP biosynthetic process | GO:0006754 | 42 | 2 | 0.18468 | biological_process |
| 37 | catalytic activity | GO:0003824 | 3510 | 12 | 0.29046 | molecular_function |
| 38 | metabolic process | GO:0008152 | 3072 | 11 | 0.2912 | biological_process |
| 39 | dihydrokaempferol 4-reductase activity | GO:0045552 | 1 | 1 | 0.2912 | molecular_function |
| 40 | transferase activity, transferring acyl groups | GO:0016746 | 440 | 4 | 0.30788 | molecular_function |
| 41 | vegetative to reproductive phase transition of meristem | GO:0010228 | 75 | 2 | 0.48925 | biological_process |
| 42 | response to absence of light | GO:0009646 | 3 | 1 | 0.53941 | biological_process |
| 43 | transporter activity | GO:0005215 | 908 | 5 | 0.72396 | molecular_function |
| 44 | regulation of isopentenyl diphosphate biosynthetic process, methylerythritol 4-phosphate pathway | GO:0010322 | 5 | 1 | 0.75537 | biological_process |
| 45 | regulation of 1-deoxy-D-xylulose-5-phosphate synthase activity | GO:1902395 | 5 | 1 | 0.75537 | biological_process |
| 46 | anther wall tapetum development | GO:0048658 | 6 | 1 | 0.844 | biological_process |
| 47 | carbohydrate metabolic process | GO:0005975 | 1391 | 6 | 0.88981 | biological_process |
| 48 | phosphoenolpyruvate carboxykinase activity | GO:0004611 | 7 | 1 | 0.88981 | molecular_function |
| 49 | phosphoenolpyruvate carboxykinase (ATP) activity | GO:0004612 | 7 | 1 | 0.88981 | molecular_function |
| 50 | purine nucleotide binding | GO:0017076 | 7 | 1 | 0.88981 | molecular_function |
| 51 | lyase activity | GO:0016829 | 671 | 4 | 0.99575 | molecular_function |

Table S9. The GO analysis of down-regulated genes in buds (wild-type vs *rdr6* plants)

| No | GO_term | GOid | All | DEG | P-value | Ontologies |
| --- | --- | --- | --- | --- | --- | --- |
| 1 | anther morphogenesis | GO:0048654 | 6 | 5 | 1.02E-09 | biological_process |
| 2 | anther wall tapetum cell differentiation | GO:0048657 | 6 | 5 | 1.02E-09 | biological_process |
| 3 | anther development | GO:0048653 | 8 | 5 | 1.89E-09 | biological_process |
| 4 | spermidine:sinapoyl CoA N-acyltransferase activity | GO:0080072 | 3 | 3 | 1.12E-05 | molecular_function |
| 5 | spermidine:coumaroyl CoA N-acyltransferase activity | GO:0080073 | 3 | 3 | 1.12E-05 | molecular_function |
| 6 | spermidine:caffeoyl CoA N-acyltransferase activity | GO:0080074 | 3 | 3 | 1.12E-05 | molecular_function |
| 7 | spermidine:feruloyl CoA N-acyltransferase activity | GO:0080075 | 3 | 3 | 1.12E-05 | molecular_function |
| 8 | spermidine hydroxycinnamate conjugate biosynthetic process | GO:0080088 | 3 | 3 | 1.12E-05 | biological_process |
| 9 | galactosyltransferase activity | GO:0008378 | 81 | 5 | 1.60E-05 | molecular_function |
| 10 | N-acyltransferase activity | GO:0016410 | 5 | 3 | 2.51E-05 | molecular_function |
| 11 | protein glycosylation | GO:0006486 | 165 | 5 | 0.000391753 | biological_process |
| 12 | pollen exine formation | GO:0010584 | 19 | 3 | 0.000568642 | biological_process |
| 13 | Golgi membrane | GO:0000139 | 267 | 5 | 0.003269351 | cellular_component |
| 14 | hydrolase activity, acting on glycosyl bonds | GO:0016798 | 739 | 7 | 0.004054067 | molecular_function |
| 15 | carbohydrate metabolic process | GO:0005975 | 1391 | 9 | 0.004539115 | biological_process |
| 16 | borate transmembrane transport | GO:0035445 | 6 | 2 | 0.007396387 | biological_process |
| 17 | borate transmembrane transporter activity | GO:0046715 | 6 | 2 | 0.007396387 | molecular_function |
| 18 | hydrolase activity, hydrolyzing O-glycosyl compounds | GO:0004553 | 869 | 7 | 0.00872675 | molecular_function |
| 19 | hydrolase activity, acting on ester bonds | GO:0016788 | 405 | 5 | 0.01574504 | molecular_function |
| 20 | cell wall macromolecule catabolic process | GO:0016998 | 25 | 2 | 0.07411734 | biological_process |
| 21 | transferase activity, transferring glycosyl groups | GO:0016757 | 953 | 6 | 0.09510598 | molecular_function |
| 22 | chitin binding | GO:0008061 | 30 | 2 | 0.09531154 | molecular_function |
| 23 | vacuole | GO:0005773 | 384 | 4 | 0.1088143 | cellular_component |
| 24 | pollen development | GO:0009555 | 157 | 3 | 0.1088143 | biological_process |
| 25 | substrate-specific transmembrane transporter activity | GO:0022891 | 169 | 3 | 0.1088143 | molecular_function |
| 26 | glycerolipid biosynthetic process | GO:0045017 | 34 | 2 | 0.1088143 | biological_process |
| 27 | phosphatidylcholine metabolic process | GO:0046470 | 36 | 2 | 0.1088143 | biological_process |
| 28 | N-acylphosphatidylethanolamine-specific phospholipase D activity | GO:0070290 | 36 | 2 | 0.1088143 | molecular_function |
| 29 | phospholipase D activity | GO:0004630 | 37 | 2 | 0.1088143 | molecular_function |
| 30 | hydrolase activity | GO:0016787 | 5182 | 14 | 0.1285412 | molecular_function |
| 31 | Golgi apparatus | GO:0005794 | 734 | 5 | 0.1334226 | cellular_component |
| 32 | diacylglycerol O-acyltransferase activity | GO:0004144 | 48 | 2 | 0.1556326 | molecular_function |
| 33 | chitin catabolic process | GO:0006032 | 59 | 2 | 0.220393 | biological_process |
| 34 | chitinase activity | GO:0004568 | 60 | 2 | 0.2214378 | molecular_function |
| 35 | ATPase activity, coupled to transmembrane movement of substances | GO:0042626 | 240 | 3 | 0.2338176 | molecular_function |
| 36 | beta-galactosidase activity | GO:0004565 | 66 | 2 | 0.2520738 | molecular_function |
| 37 | transporter activity | GO:0005215 | 908 | 5 | 0.2759692 | molecular_function |
| 38 | cell wall | GO:0005618 | 559 | 4 | 0.2907729 | cellular_component |
| 39 | polysaccharide catabolic process | GO:0000272 | 88 | 2 | 0.3961511 | biological_process |
| 40 | transferase activity, transferring acyl groups other than amino-acyl groups | GO:0016747 | 306 | 3 | 0.3965759 | molecular_function |
| 41 | metabolic process | GO:0008152 | 3072 | 9 | 0.4351129 | biological_process |
| 42 | transmembrane transporter activity | GO:0022857 | 355 | 3 | 0.5698521 | molecular_function |
| 43 | positive regulation of protein kinase activity | GO:0045860 | 5 | 1 | 0.5742914 | biological_process |
| 44 | protein kinase activator activity | GO:0030295 | 6 | 1 | 0.6557206 | molecular_function |
| 45 | transferase activity, transferring hexosyl groups | GO:0016758 | 399 | 3 | 0.73412 | molecular_function |
| 46 | cinnamic acid biosynthetic process | GO:0009800 | 8 | 1 | 0.7627721 | biological_process |
| 47 | ammonia-lyase activity | GO:0016841 | 8 | 1 | 0.7627721 | molecular_function |
| 48 | phenylalanine ammonia-lyase activity | GO:0045548 | 8 | 1 | 0.7627721 | molecular_function |
| 49 | metal ion binding | GO:0046872 | 7430 | 1 | 0.7627721 | molecular_function |
| 50 | transport | GO:0006810 | 2879 | 8 | 0.7849578 | biological_process |
| 51 | lipid catabolic process | GO:0016042 | 151 | 2 | 0.862696 | biological_process |
| 52 | L-phenylalanine catabolic process | GO:0006559 | 11 | 1 | 0.9442438 | biological_process |
| 53 | reductive pentose-phosphate cycle | GO:0019253 | 11 | 1 | 0.9442438 | biological_process |

Table S10. The top ten gene ontology terms among the differentially expressed genes^†^ (wild-type vs *rdr6*)

|  | GO_term^‡^ (All, DEGs^§^) | GO_term (All, DEGs) | GO_term (All, DEGs) |
| --- | --- | --- | --- |
|  | Leaves | Flowers | Buds |
| UP | **auxin-activated signaling pathway (326, 4)**  vegetative phase change (8, 2)  abaxial cell fate specification (10, 2)  negative regulation of endopeptidase activity (174, 3)  serine-type endopeptidase inhibitor activity (44, 2)  glutathione transferase activity (100, 2)  response to hormones (148, 2)  transferase activity (4549, 3)  chloroplast (2576, 2)  transcription, DNA-templated (2881, 2) | vegetative phase change (8, 6)  abaxial cell fate specification (10, 6)  response to hormones (148, 8)  **auxin-activated signaling pathway** (326, 8)  floral meristem determinacy (22, 4)  anchored component of the membrane (48, 3)  cellulose microfibril organization (60, 3)  cell growth (84, 3)  alpha-galactosidase activity (15, 2)  raffinose alpha-galactosidase activity (15, 2) | pectin catabolic process (212, 10)  pollen exine formation (19, 4)  cell wall modification (152, 6)  aspartyl esterase activity (152, 6)  pectinesterase activity (154, 6)  extracellular space (90, 5)  cystathionine beta-lyase activity (6, 3)  'de novo' L-methionine biosynthetic process (6, 3)  enzyme inhibitor activity (195, 6)  sporopollenin biosynthetic process (9, 3) |
| DOWN | transferase activity (4549, 3)  transferase activity, transferring acyl groups (440, 2)  nucleotide binding (6085, 2)  transcription, RNA-templated (1)  RNA-directed RNA polymerase activity (18, 1)  methionine adenosyltransferase activity (18, 1)  S-adenosylmethionine biosynthetic process (19, 1)  secondary active sulfate transmembrane transporter activity (19, 1)  sulfate transport (61, 1)  sulfate transmembrane transporter activity (61, 1) | carbohydrate metabolic process (1391, 10)  apoplast (279, 6)  seed coat development (10, 3)  hydrolase activity, hydrolyzing O-glycosyl compounds (869, 8)  vacuole (384, 6)  alpha-L-arabinofuranosidase activity (19, 3)  hydrolase activity, acting on glycosyl bonds (739, 7)  chitin binding (30, 3)  hydrolase activity (5182, 16)  1-aminocyclopropane-1-carboxylate oxidase activity (3, 2) | anther morphogenesis (6, 5)  anther wall tapetum cell differentiation (6, 5)  anther development (8, 5)  spermidine: sinapoyl CoA N-acyltransferase activity (3, 3)  spermidine: coumaroyl CoA N-acyltransferase activity (3, 3)  spermidine: caffeoyl CoA N-acyltransferase activity (3, 3)  spermidine: feruloyl CoA N-acyltransferase activity (3, 3)  spermidine hydroxycinnamate conjugate biosynthetic process (3, 3)  galactosyltransferase activity (81, 5)  N-acyltransferase activity (5, 3) |

^†^Sorted by p-values; ^‡^ GO – gene ontology; ^§^ DEGs – differentially expressed genes

Table S11. The top ten gene ontology terms among the differentially expressed genes^†^ (wild-type vs TAS3i plants)

|  | GO_term^‡^ (All, DEGs)^§^ | GO_term (All, DEGs) | GO_term (All, DEGs) |
| --- | --- | --- | --- |
|  | Leaves | Flowers | Buds |
| UP | carbon utilization (29, 11)  carbonate dehydratase activity (56, 11)  serine-type endopeptidase inhibitor activity (44, 8)  negative regulation of endopeptidase activity (174, 10)  lyase activity (671, 13)  killing of cells of other organisms (46, 3)  response to wounding (134, 3)  glucose-1-phosphate adenylyltransferase activity (41, 2)  defense response to fungus (175, 3)  endopeptidase inhibitor activity (31, 2) | adenylate cyclase activity (5, 2)  cAMP biosynthetic process (5, 2)  integral component of membrane (22126, 4)  membrane (22913, 4)  lyase activity (671, 2)  chloroplast (2576, 2)  central vacuole (9, 1)  cellular water homeostasis (19, 1)  water channel activity (19, 1)  glycerol channel activity (19, 1) | pollen exine formation (19, 4)  cell wall macromolecule catabolic process (25, 4)  chitin binding (30, 4)  sporopollenin biosynthetic process (9, 3)  chitin catabolic process (59, 4)  chitinase activity (60, 4)  negative regulation of transposition (2, 2)  polyketide biosynthetic process (2, 2)  seed trichome initiation (2, 2)  tetraketide alpha-pyrone synthase activity (2, 2) |
| DOWN | vegetative phase change (8, 6)  abaxial cell fate specification (10, 6)  response to hormones (148, 8)  **auxin-activated signaling pathway (326, 8**)  floral meristem determinacy (22, 4)  transcription, DNA-templated (2881, 8)  regulation of transcription, DNA-templated (4728, 8)  DNA binding (6192, 8)  nucleus (7597, 8)  transcription factor activity, sequence-specific DNA binding (2085, 4) | chitin binding (30, 6)  response to biotic stimulus (158, 7)  defense response (1665, 14)  cell wall macromolecule catabolic process (25, 4)  hydrolase activity, acting on glycosyl bonds (739, 9)  carbohydrate metabolic process (1391, 11)  vacuole (384, 7)  apoplast (279, 6)  chitinase activity (60, 4)  chitin catabolic process (59, 4) | anther wall tapetum cell differentiation (6, 6)  anther morphogenesis (6, 6)  anther development (8, 6)  vegetative phase change (8, 6)  abaxial cell fate specification (10, 6)  pollen exine formation (19, 6)  cellular ion homeostasis (15, 5)  response to hormones (148, 8)  voltage-gated anion channel activity (19, 5)  carbohydrate metabolic process (1391, 16) |

^†^Sorted by p-values; ^‡^ GO – gene ontology; ^§^ DEGs – differentially expressed genes

Table S12. The differentially expressed genes in leaves of wild-type plants vs TAS3i plants

|  |  |  |  | expression amounts in leaves of | |  |  |
| --- | --- | --- | --- | --- | --- | --- | --- |
|  | Gene_ID | Gene | Gbkey | wild type plants | TAS3i plants | m.value [log2(expression ratio)] | Product |
| 1 | rna-XM_016641299.1 | LOC107815675 | mRNA | 0 | 202 | 7.531 | zinc finger BED domain-containing protein RICESLEEPER 3-like |
| 2 | rna-XM_016647397.1 | LOC107821006 | mRNA | 1 | 93 | 6.412 | wound-induced proteinase inhibitor 2-like, transcript variant X3 |
| 3 | rna-XM_016647395.1 | LOC107821006 | mRNA | 1 | 93 | 6.412 | wound-induced proteinase inhibitor 2-like, transcript variant X1 |
| 4 | rna-XM_016647396.1 | LOC107821006 | mRNA | 1 | 93 | 6.412 | wound-induced proteinase inhibitor 2-like, transcript variant X2 |
| 5 | rna-XM_016613856.1 | LOC107791735 | mRNA | 3 | 129 | 5.299 | defensin-like protein |
| 6 | rna-XM_016659547.1 | LOC107831753 | mRNA | 9 | 322 | 5.034 | defensin-like protein |
| 7 | rna-XM_016623020.1 | LOC107799889 | mRNA | 13 | 369 | 4.700 | cysteine protease inhibitor 8-like |
| 8 | rna-XM_016642073.1 | LOC107816366 | mRNA | 50 | 1341 | 4.618 | cysteine protease inhibitor 8-like |
| 9 | rna-XM_016655118.1 | LOC107827891 | mRNA | 141 | 3486 | 4.500 | proteinase inhibitor I-B-like |
| 10 | rna-XM_016659546.1 | LOC107831752 | mRNA | 5 | 116 | 4.409 | defensin-like protein |
| 11 | rna-XM_016618457.1 | LOC107795773 | mRNA | 205 | 3740 | 4.062 | uncharacterized LOC107795773 |
| 12 | rna-XM_016621555.1 | LOC107798543 | mRNA | 5 | 73 | 3.741 | (-)-camphene/tricyclene synthase, chloroplastic-like |
| 13 | rna-XM_016612493.1 | LOC107790557 | mRNA | 127 | 1827 | 3.719 | proteinase inhibitor I-B-like |
| 14 | rna-XM_016602129.1 | LOC107781430 | mRNA | 286 | 3974 | 3.669 | wound-induced proteinase inhibitor 2-like |
| 15 | rna-XM_016614664.1 | LOC107792454 | mRNA | 205 | 2773 | 3.630 | wound-induced proteinase inhibitor 2-like |
| 16 | rna-XM_016659003.1 | LOC107831250 | mRNA | 6 | 80 | 3.610 | (-)-alpha-terpineol synthase-like |
| 17 | rna-XM_016637536.1 | LOC107812321 | mRNA | 255 | 2506 | 3.169 | carbonic anhydrase, chloroplastic-like, transcript variant X3 |
| 18 | rna-XM_016655152.1 | LOC107827919 | mRNA | 10 | 92 | 3.074 | putative UPF0481 protein At3g02645, transcript variant X2 |
| 19 | rna-XM_016655151.1 | LOC107827919 | mRNA | 10 | 92 | 3.074 | putative UPF0481 protein At3g02645, transcript variant X1 |
| 20 | rna-XM_016631900.1 | LOC107807494 | mRNA | 28 | 251 | 3.037 | pelargonidin 3-O-(6-caffeoylglucoside) 5-O-(6-O-malonylglucoside) 4'''-malonyltransferase-like |
| 21 | rna-XM_016637608.1 | LOC107812321 | mRNA | 328 | 2831 | 2.982 | carbonic anhydrase, chloroplastic-like, transcript variant X4 |
| 22 | rna-XM_016637681.1 | LOC107812321 | mRNA | 328 | 2831 | 2.982 | carbonic anhydrase, chloroplastic-like, transcript variant X5 |
| 23 | rna-XM_016637404.1 | LOC107812321 | mRNA | 328 | 2831 | 2.982 | carbonic anhydrase, chloroplastic-like, transcript variant X1 |
| 24 | rna-XM_016637478.1 | LOC107812321 | mRNA | 328 | 2831 | 2.982 | carbonic anhydrase, chloroplastic-like, transcript variant X2 |
| 25 | rna-XM_016588188.1 | LOC107769016 | mRNA | 132 | 920 | 2.674 | beta carbonic anhydrase 6, mitochondrial-like, transcript variant X1 |
| 26 | rna-XM_016588189.1 | LOC107769016 | mRNA | 132 | 920 | 2.674 | beta carbonic anhydrase 6, mitochondrial-like, transcript variant X2 |
| 27 | rna-XR_001650672.1 | LOC107797352 | ncRNA | 317 | 1994 | 2.526 | uncharacterized LOC107797352 |
| 28 | rna-XM_016630746.1 | LOC107806576 | mRNA | 34 | 213 | 2.520 | carbonic anhydrase, chloroplastic-like |
| 29 | rna-XM_016586888.1 | LOC107767796 | mRNA | 138 | 762 | 2.338 | carbonic anhydrase, chloroplastic-like, transcript variant X1 |
| 30 | rna-XM_016586889.1 | LOC107767796 | mRNA | 138 | 762 | 2.338 | carbonic anhydrase, chloroplastic-like, transcript variant X2 |
| 31 | rna-XM_016619110.1 | LOC107796351 | mRNA | 1697 | 8673 | 2.226 | proteinase inhibitor I-B-like |
| 32 | gene-LOC107812172 | LOC107812172 | exon | 51 | 209 | 1.908 |  |
| 33 | rna-XM_016655179.1 | LOC107827944 | mRNA | 899 | 3603 | 1.875 | putative UDP-rhamnose:rhamnosyltransferase 1 |
| 34 | rna-XM_016589864.1 | LOC107770515 | mRNA | 138 | 468 | 1.634 | beta-amyrin synthase-like, transcript variant X1 |
| 35 | rna-XM_016589922.1 | LOC107770515 | mRNA | 138 | 468 | 1.634 | beta-amyrin synthase-like, transcript variant X2 |
| 36 | rna-XM_016618800.1 | LOC107796079 | mRNA | 731 | 2380 | 1.576 | UDP-glycosyltransferase 74E2-like |
| 37 | rna-XM_016595792.1 | LOC107775984 | mRNA | 104 | 334 | 1.556 | annexin D4-like |
| 38 | rna-XM_016656810.1 | LOC107829337 | mRNA | 114 | 362 | 1.540 | UDP-glycosyltransferase 74E2-like |
| 39 | rna-XM_016578154.1 | LOC107760136 | mRNA | 453 | 1377 | 1.477 | transmembrane protein 184C-like |
| 40 | rna-XM_016617806.1 | LOC107795210 | mRNA | 242 | 661 | 1.322 | alcohol dehydrogenase-like |
| 41 | rna-XM_016653552.1 | LOC107826565 | mRNA | 1142 | 2681 | 1.104 | glucose-1-phosphate adenylyltransferase large subunit 1-like, transcript variant X1 |
| 42 | rna-XM_016653553.1 | LOC107826565 | mRNA | 1141 | 2673 | 1.101 | glucose-1-phosphate adenylyltransferase large subunit 1-like, transcript variant X2 |
| 1 | rna-XM_016626020.1 | LOC107802501 | mRNA | 3768 | 1864 | -1.143 | aquaporin TIP2-1-like |
| 2 | rna-XM_016640248.1 | LOC107814785 | mRNA | 1822 | 885 | -1.169 | aquaporin TIP2-1-like |
| 3 | rna-XM_016621605.1 | LOC107798595 | mRNA | 1488 | 666 | -1.287 | uncharacterized LOC107798595 |
| 4 | rna-XM_016621745.1 | LOC107798716 | mRNA | 287 | 83 | -1.917 | RNA-dependent RNA polymerase 6 |
| 5 | rna-XR_001647814.1 | LOC107785172 | misc_RNA | 259 | 71 | -1.994 | **auxin response factor 3-like, transcript variant X1** |
| 6 | rna-XR_001647815.1 | LOC107785172 | misc_RNA | 259 | 71 | -1.994 | **auxin response factor 3-like, transcript variant X3** |
| 7 | rna-XM_016606410.1 | LOC107785172 | mRNA | 259 | 71 | -1.994 | **auxin response factor 3-like, transcript variant X2** |
| 8 | rna-XM_016627510.1 | LOC107803745 | mRNA | 484 | 125 | -2.080 | **auxin response factor 3-like, transcript variant X2** |
| 9 | rna-XR_001652067.1 | LOC107803745 | misc_RNA | 484 | 124 | -2.092 | **auxin response factor 3-like, transcript variant X1** |
| 10 | rna-XR_001652068.1 | LOC107803745 | misc_RNA | 484 | 124 | -2.092 | **auxin response factor 3-like, transcript variant X3** |
| 11 | rna-XM_016653169.1 | LOC107826218 | mRNA | 236 | 51 | -2.338 | **auxin response factor 4-like** |
| 12 | rna-XM_016606813.1 | LOC107785490 | mRNA | 293 | 55 | -2.541 | **auxin response factor 4-like** |

Table S13. The differentially expressed genes in flowers of wild-type plants vs TAS3i plants

|  |  |  |  | expression amounts in flowers of | |  |  |
| --- | --- | --- | --- | --- | --- | --- | --- |
|  | Gene_ID | Gene | Gbkey | wild type plants | TAS3i plants | m.value [log2(expression ratio)] | Product |
| 1 | rna-XM_016641299.1 | LOC107815675 | mRNA | 0 | 207 | 7.282 | zinc finger BED domain-containing protein RICESLEEPER 3-like |
| 2 | rna-XM_016638433.1 | LOC107813195 | mRNA | 1 | 109 | 6.357 | uncharacterized LOC107813195, transcript variant X2 |
| 3 | rna-XM_016638425.1 | LOC107813195 | mRNA | 1 | 109 | 6.357 | uncharacterized LOC107813195, transcript variant X1 |
| 4 | rna-XM_016655152.1 | LOC107827919 | mRNA | 25 | 209 | 2.652 | putative UPF0481 protein At3g02645, transcript variant X2 |
| 5 | rna-XM_016655151.1 | LOC107827919 | mRNA | 25 | 209 | 2.652 | putative UPF0481 protein At3g02645, transcript variant X1 |
| 6 | rna-XM_016623090.1 | LOC107799946 | mRNA | 139 | 625 | 1.757 | protein phosphatase 1 regulatory subunit pprA-like, transcript variant X2 |
| 7 | rna-XM_016623089.1 | LOC107799946 | mRNA | 139 | 625 | 1.757 | protein phosphatase 1 regulatory subunit pprA-like, transcript variant X1 |
| 8 | rna-XM_016647624.1 | LOC107821202 | mRNA | 388 | 1459 | 1.499 | galactinol synthase 2 |
| 9 | rna-XM_016631569.1 | LOC107807226 | mRNA | 262 | 982 | 1.495 | aquaporin TIP1-3-like |
| 10 | rna-XM_016613856.1 | LOC107791735 | mRNA | 656 | 2127 | 1.286 | defensin-like protein |
| 1 | rna-XM_016644886.1 | LOC107818832 | mRNA | 3598 | 2069 | -1.210 | beta-D-xylosidase 1-like |
| 2 | rna-XM_016599645.1 | LOC107779248 | mRNA | 7832 | 4474 | -1.219 | senescence-specific cysteine protease SAG12-like |
| 3 | rna-XM_016607761.1 | LOC107786280 | mRNA | 5066 | 2891 | -1.221 | senescence-specific cysteine protease SAG12-like |
| 4 | rna-XM_016649544.1 | LOC107822960 | mRNA | 1686 | 950 | -1.239 | beta-D-xylosidase 1-like, transcript variant X1 |
| 5 | rna-XM_016600359.1 | LOC107779861 | mRNA | 888 | 468 | -1.336 | non-specific lipid-transfer protein 3-like |
| 6 | rna-XM_016596103.1 | LOC107776233 | mRNA | 590 | 300 | -1.387 | sulfate transporter 3.1-like |
| 7 | rna-XM_016646551.1 | LOC107820290 | mRNA | 929 | 469 | -1.398 | protein EXORDIUM-like 5 |
| 8 | rna-XM_016634720.1 | LOC107810003 | mRNA | 382 | 178 | -1.513 | protein BPS1, chloroplastic-like |
| 9 | rna-XM_016577348.1 | LOC107759406 | mRNA | 641 | 293 | -1.541 | intracellular ribonuclease LX-like |
| 10 | rna-XM_016577916.1 | LOC107759913 | mRNA | 906 | 413 | -1.545 | 1-aminocyclopropane-1-carboxylate oxidase-like |
| 11 | rna-XM_016591148.1 | LOC107771705 | mRNA | 481 | 218 | -1.553 | snakin-2-like |
| 12 | rna-XM_016629138.1 | LOC107805144 | mRNA | 455 | 200 | -1.597 | pectin acetylesterase 8-like |
| 13 | rna-XM_016601774.1 | LOC107781126 | mRNA | 1448 | 540 | -1.835 | 1-aminocyclopropane-1-carboxylate oxidase |
| 14 | rna-XM_016649299.1 | LOC107822731 | mRNA | 709 | 240 | -1.974 | protein EXORDIUM-like |
| 15 | rna-XM_016613522.1 | LOC107791444 | mRNA | 426 | 142 | -1.996 | wound-induced protein 1-like |
| 16 | rna-XM_016645620.1 | LOC107819508 | mRNA | 246 | 80 | -2.032 | beta-amyrin 28-oxidase-like |
| 17 | rna-XM_016655666.1 | LOC107828379 | mRNA | 209 | 58 | -2.261 | protein trichome birefringence-like 41 |
| 18 | rna-XM_016624212.1 | LOC107800941 | mRNA | 116 | 31 | -2.315 | protein EXORDIUM-like |
| 19 | rna-XM_016656264.1 | LOC107828876 | mRNA | 247 | 66 | -2.315 | protein EXORDIUM-like |
| 20 | rna-XM_016649300.1 | LOC107822732 | mRNA | 153 | 35 | -2.540 | protein EXORDIUM-like |
| 21 | rna-XM_016591891.1 | LOC107772392 | mRNA | 117 | 26 | -2.581 | probable glutathione S-transferase, transcript variant X1 |
| 22 | rna-XM_016630594.1 | LOC107806436 | mRNA | 480 | 77 | -3.052 | indole-3-acetic acid-amido synthetase GH3.6-like |
| 23 | rna-XM_016615641.1 | LOC107793308 | mRNA | 577 | 87 | -3.141 | pectinesterase 2-like |
| 24 | rna-XM_016634470.1 | LOC107809780 | mRNA | 141 | 21 | -3.159 | lichenase-like |
| 25 | rna-XM_016589414.1 | LOC107770142 | mRNA | 271 | 29 | -3.636 | pectinesterase 2-like |
| 26 | rna-XM_016641812.1 | LOC107816122 | mRNA | 519 | 53 | -3.703 | proteinase inhibitor type-2 |
| 27 | rna-XM_016624663.1 | LOC107801355 | mRNA | 266 | 26 | -3.766 | protein NEN4-like |
| 28 | rna-XM_016643194.1 | LOC107817388 | mRNA | 155 | 15 | -3.781 | indole-3-acetic acid-amido synthetase GH3.6-like |
| 29 | rna-XM_016639520.1 | LOC107814170 | mRNA | 63 | 6 | -3.804 | uclacyanin-3-like |
| 30 | rna-XM_016658347.1 | LOC107830713 | mRNA | 119 | 11 | -3.847 | laccase-14-like |
| 31 | rna-XM_016640264.1 | LOC107814806 | mRNA | 122 | 11 | -3.883 | lichenase |
| 32 | rna-XM_016641890.1 | LOC107816200 | mRNA | 50 | 4 | -4.055 | probable protein phosphatase 2C 72 |
| 33 | rna-XM_016616341.1 | LOC107793895 | mRNA | 54 | 4 | -4.166 | suberization-associated anionic peroxidase-like |
| 34 | rna-XM_016605567.1 | LOC107784434 | mRNA | 118 | 8 | -4.294 | endochitinase 3 |
| 35 | rna-XM_016588905.1 | LOC107769668 | mRNA | 66 | 4 | -4.456 | alpha-amylase-like |
| 36 | rna-XM_016591618.1 | LOC107772133 | mRNA | 452 | 19 | -4.984 | wound-induced protein WIN1-like |
| 37 | rna-XM_016600920.1 | LOC107780391 | mRNA | 149 | 6 | -5.046 | endochitinase 3-like |
| 38 | rna-XM_016624436.1 | LOC107801151 | mRNA | 152 | 5 | -5.337 | glucan endo-1,3-beta-glucosidase, basic vacuolar |
| 39 | rna-XM_016614910.1 | LOC107792673 | mRNA | 135 | 4 | -5.488 | laccase-7-like |
| 40 | rna-XM_016644720.1 | LOC107818683 | mRNA | 92 | 2 | -5.935 | laccase-7-like |
| 41 | rna-XM_016653822.1 | LOC107826794 | mRNA | 138 | 3 | -5.935 | endochitinase B |
| 42 | rna-XM_016650053.1 | LOC107823411 | mRNA | 559 | 11 | -6.079 | glucan endo-1,3-beta-glucosidase, basic vacuolar |
| 43 | rna-XM_016609426.1 | LOC107787819 | mRNA | 359 | 7 | -6.092 | osmotin |
| 44 | rna-XM_016616741.1 | LOC107794263 | mRNA | 117 | 2 | -6.282 | wound-induced protein WIN1-like |
| 45 | rna-XM_016606113.1 | LOC107784916 | mRNA | 69 | 1 | -6.520 | laccase-14-like |
| 46 | rna-XM_016576862.1 | LOC107759005 | mRNA | 129 | 1 | -7.423 | endochitinase A |
| 47 | rna-XM_016609409.1 | LOC107787802 | mRNA | 62 | 0 | -6.366 | osmotin-like protein |
| 48 | rna-XM_016609419.1 | LOC107787812 | mRNA | 97 | 0 | -7.011 | osmotin-like protein |
| 49 | rna-XM_016616970.1 | LOC107794477 | mRNA | 43 | 0 | -5.838 | osmotin-like protein |
| 50 | rna-XM_016616971.1 | LOC107794478 | mRNA | 67 | 0 | -6.478 | osmotin-like protein |
| 51 | rna-XM_016616972.1 | LOC107794479 | mRNA | 34 | 0 | -5.499 | osmotin-like |
| 52 | rna-XM_016635289.1 | LOC107810500 | mRNA | 78 | 0 | -6.697 | polyphenol oxidase, chloroplastic-like |
| 53 | rna-XM_016635290.1 | LOC107810501 | mRNA | 53 | 0 | -6.139 | polyphenol oxidase, chloroplastic-like |

Table S14. The differentially expressed genes in buds of wild-type plants vs TAS3i plants

|  |  |  |  | expression amounts in buds of | |  |  |
| --- | --- | --- | --- | --- | --- | --- | --- |
|  | Gene_ID | Gene | Gbkey | wild type plants | TAS3i plants | m.value [log2(expression ratio)] | Product |
| 1 | rna-XM_016605916.1 | LOC107784740 | mRNA | 1 | 922 | 9.940 | stamen-specific protein FIL1-like |
| 2 | rna-XM_016611858.1 | LOC107789975 | mRNA | 0 | 619 | 9.365 | endochitinase EP3-like |
| 3 | rna-XM_016639596.1 | LOC107814221 | mRNA | 2 | 1005 | 9.064 | protein 108-like |
| 4 | rna-XM_016623749.1 | LOC107800558 | mRNA | 0 | 453 | 8.915 | glycine-rich cell wall structural protein-like |
| 5 | rna-XM_016609596.1 | LOC107787967 | mRNA | 3 | 936 | 8.377 | patatin-like protein 2 |
| 6 | rna-XM_016637635.1 | LOC107812512 | mRNA | 1 | 249 | 8.051 | protein trichome birefringence-like 28 |
| 7 | rna-XM_016621411.1 | LOC107798423 | mRNA | 5 | 1202 | 8.000 | protein LIM1-like |
| 8 | rna-XM_016602246.1 | LOC107781529 | mRNA | 0 | 192 | 7.676 | AT-hook motif nuclear-localized protein 16-like |
| 9 | rna-XM_016641299.1 | LOC107815675 | mRNA | 0 | 129 | 7.102 | zinc finger BED domain-containing protein RICESLEEPER 3-like |
| 10 | rna-XM_016609278.1 | LOC107787674 | mRNA | 4 | 448 | 6.899 | glycine-rich cell wall structural protein-like |
| 11 | rna-XM_016578483.1 | LOC107760438 | mRNA | 0 | 104 | 6.792 | transcription repressor MYB4-like |
| 12 | rna-XM_016609793.1 | LOC107788133 | mRNA | 0 | 96 | 6.676 | AT-hook motif nuclear-localized protein 16-like |
| 13 | rna-XM_016578721.1 | LOC107760638 | mRNA | 1 | 92 | 6.615 | endochitinase EP3-like |
| 14 | rna-XM_016611533.1 | LOC107789674 | mRNA | 8 | 619 | 6.365 | protein TAP1-like |
| 15 | rna-XM_016586702.1 | LOC107767635 | mRNA | 7 | 487 | 6.212 | subtilisin-like protease SBT2.5 |
| 16 | rna-XM_016641408.1 | LOC107815778 | mRNA | 0 | 68 | 6.179 | putative non-specific lipid-transfer protein 14 |
| 17 | rna-XM_016583818.1 | LOC107765199 | mRNA | 2 | 99 | 5.721 | probable methyltransferase PMT15, transcript variant X2 |
| 18 | rna-XM_016583817.1 | LOC107765199 | mRNA | 2 | 99 | 5.721 | probable methyltransferase PMT15, transcript variant X1 |
| 19 | rna-XM_016650678.1 | LOC107823969 | mRNA | 0 | 43 | 5.517 | patatin-like protein 2 |
| 20 | rna-XM_016596370.1 | LOC107776467 | mRNA | 1 | 43 | 5.517 | uncharacterized LOC107776467 |
| 21 | gene-LOC107796867 | LOC107796867 | exon | 6 | 252 | 5.483 |  |
| 22 | rna-XM_016596613.1 | LOC107776702 | mRNA | 5 | 209 | 5.477 | putative non-specific lipid-transfer protein 14 |
| 23 | rna-XM_016620172.1 | LOC107797301 | mRNA | 0 | 41 | 5.449 | protein trichome birefringence-like 28 |
| 24 | rna-XM_016593863.1 | LOC107774364 | mRNA | 0 | 35 | 5.220 | vinorine synthase-like |
| 25 | rna-XM_016591162.1 | LOC107771719 | mRNA | 11 | 268 | 4.698 | type III polyketide synthase B-like |
| 26 | rna-XM_016593621.1 | LOC107774140 | mRNA | 2 | 45 | 4.583 | probable methyltransferase PMT15 |
| 27 | rna-XM_016656209.1 | LOC107828833 | mRNA | 18 | 399 | 4.561 | glycine-rich cell wall structural protein-like |
| 28 | rna-XM_016626815.1 | LOC107803179 | mRNA | 12 | 260 | 4.529 | type III polyketide synthase B-like |
| 29 | rna-XM_016635773.1 | LOC107810938 | mRNA | 3 | 53 | 4.234 | glycine-rich cell wall structural protein 1-like |
| 30 | rna-XM_016656108.1 | LOC107828740 | mRNA | 24 | 384 | 4.091 | LRR receptor-like serine/threonine-protein kinase EFR |
| 31 | rna-XM_016578732.1 | LOC107760646 | mRNA | 8 | 123 | 4.034 | chitinase 6-like |
| 32 | rna-XM_016584260.1 | LOC107765592 | mRNA | 7 | 94 | 3.838 | glycine-rich protein DOT1-like |
| 33 | rna-XM_016615552.1 | LOC107793239 | mRNA | 11 | 131 | 3.665 | leucine-rich repeat receptor-like serine/threonine-protein kinase BAM1 |
| 34 | rna-XM_016648345.1 | LOC107821889 | mRNA | 11 | 118 | 3.514 | protein STRICTOSIDINE SYNTHASE-LIKE 13-like |
| 35 | rna-XM_016598064.1 | LOC107777898 | mRNA | 16 | 118 | 2.974 | chitinase 4-like |
| 36 | rna-XM_016627075.1 | LOC107803374 | mRNA | 24 | 162 | 2.846 | subtilisin-like protease SBT2.5 |
| 37 | rna-XM_016603345.1 | LOC107782459 | mRNA | 17 | 109 | 2.772 | mannan endo-1,4-beta-mannosidase 5-like |
| 38 | rna-XM_016579984.1 | LOC107761721 | mRNA | 88 | 537 | 2.700 | protein STRICTOSIDINE SYNTHASE-LIKE 13-like |
| 39 | rna-XM_016591649.1 | LOC107772158 | mRNA | 28 | 163 | 2.633 | mavicyanin-like |
| 40 | rna-XM_016627603.1 | LOC107803813 | mRNA | 225 | 1291 | 2.612 | 4-coumarate--CoA ligase-like 1 |
| 41 | rna-XM_016613468.1 | LOC107791402 | mRNA | 19 | 108 | 2.598 | abscisic acid 8'-hydroxylase 1-like |
| 42 | rna-XM_016639446.1 | LOC107814113 | mRNA | 161 | 840 | 2.474 | 4-coumarate--CoA ligase-like 1 |
| 43 | rna-XM_016651854.1 | LOC107825022 | mRNA | 22 | 100 | 2.276 | transcription factor MYB35-like |
| 44 | rna-XM_016580401.1 | LOC107762081 | mRNA | 42 | 164 | 2.056 | mannan endo-1,4-beta-mannosidase 5-like |
| 45 | rna-XM_016602640.1 | LOC107781842 | mRNA | 35 | 127 | 1.951 | uncharacterized LOC107781842 |
| 46 | rna-XM_016652482.1 | LOC107825604 | mRNA | 41 | 142 | 1.883 | endoglucanase 11-like |
| 47 | rna-XM_016623090.1 | LOC107799946 | mRNA | 149 | 455 | 1.702 | protein phosphatase 1 regulatory subunit pprA-like, transcript variant X2 |
| 48 | rna-XM_016623089.1 | LOC107799946 | mRNA | 149 | 455 | 1.702 | protein phosphatase 1 regulatory subunit pprA-like, transcript variant X1 |
| 49 | rna-XM_016581465.1 | LOC107763048 | mRNA | 189 | 565 | 1.671 | tetraketide alpha-pyrone reductase 2-like |
| 50 | rna-XM_016613202.1 | LOC107791187 | mRNA | 100 | 259 | 1.464 | pectinesterase 2.1-like |
| 51 | rna-XM_016657558.1 | LOC107830074 | mRNA | 248 | 627 | 1.429 | uncharacterized LOC107830074, transcript variant X1 |
| 52 | rna-XM_016657559.1 | LOC107830074 | mRNA | 228 | 563 | 1.395 | uncharacterized LOC107830074, transcript variant X2 |
| 53 | rna-XM_016610823.1 | LOC107789055 | mRNA | 138 | 329 | 1.345 | laccase-11-like |
| 54 | rna-XM_016605919.1 | LOC107784744 | mRNA | 141 | 326 | 1.300 | short-chain dehydrogenase reductase ATA1-like |
| 55 | rna-XM_016636360.1 | LOC107811434 | mRNA | 281 | 606 | 1.200 | laccase-4-like |
| 56 | rna-XM_016588315.1 | LOC107769133 | mRNA | 191 | 400 | 1.158 | cellulose synthase A catalytic subunit 7 [UDP-forming] |
| 57 | rna-XM_016606355.1 | LOC107785130 | mRNA | 286 | 596 | 1.150 | uncharacterized LOC107785130 |
| 58 | rna-XM_016635596.1 | LOC107810774 | mRNA | 220 | 451 | 1.127 | caffeoyl-CoA O-methyltransferase 6 |
| 59 | rna-XM_016586485.1 | LOC107767471 | mRNA | 371 | 749 | 1.105 | isoflavone reductase-like protein |
| 60 | rna-XM_016648633.1 | LOC107822129 | mRNA | 640 | 1256 | 1.064 | isoflavone reductase-like protein |
| 61 | rna-XM_016642891.1 | LOC107817116 | mRNA | 420 | 773 | 0.971 | metallothionein-like protein type 2 |
| 1 | rna-XM_016656244.1 | LOC107828858 | mRNA | 6196 | 3550 | -0.712 | protein 108-like |
| 2 | rna-XM_016578217.1 | LOC107760202 | mRNA | 4472 | 2528 | -0.732 | putative UDP-rhamnose:rhamnosyltransferase 1 |
| 3 | rna-XM_016632484.1 | LOC107808002 | mRNA | 3800 | 2138 | -0.739 | aspartic proteinase A1-like |
| 4 | rna-XM_016642256.1 | LOC107816532 | mRNA | 1129 | 583 | -0.862 | probable cellulose synthase A catalytic subunit 9 [UDP-forming] |
| 5 | rna-XM_016592743.1 | LOC107773310 | mRNA | 806 | 395 | -0.938 | transcription factor ABORTED MICROSPORES-like |
| 6 | rna-XM_016659974.1 | LOC107832164 | mRNA | 2165 | 1058 | -0.942 | transcription factor ABORTED MICROSPORES-like, transcript variant X4 |
| 7 | rna-XM_016659971.1 | LOC107832164 | mRNA | 2165 | 1058 | -0.942 | transcription factor ABORTED MICROSPORES-like, transcript variant X1 |
| 8 | rna-XM_016659972.1 | LOC107832164 | mRNA | 2165 | 1058 | -0.942 | transcription factor ABORTED MICROSPORES-like, transcript variant X2 |
| 9 | rna-XM_016607369.1 | LOC107785940 | mRNA | 993 | 485 | -0.943 | glutelin type-B 2-like |
| 10 | rna-XM_016659976.1 | LOC107832164 | mRNA | 2165 | 1056 | -0.945 | transcription factor ABORTED MICROSPORES-like, transcript variant X5 |
| 11 | rna-XM_016659973.1 | LOC107832164 | mRNA | 2165 | 1056 | -0.945 | transcription factor ABORTED MICROSPORES-like, transcript variant X3 |
| 12 | rna-XM_016658955.1 | LOC107831213 | mRNA | 1201 | 585 | -0.947 | phenylalanine ammonia-lyase G4-like |
| 13 | rna-XM_016650815.1 | LOC107824090 | mRNA | 755 | 364 | -0.961 | Niemann-Pick C1 protein-like |
| 14 | rna-XM_016622107.1 | LOC107799043 | mRNA | 688 | 329 | -0.973 | heparanase-like protein 2 |
| 15 | rna-XM_016650109.1 | LOC107823443 | mRNA | 5568 | 2597 | -1.009 | protein 108-like |
| 16 | rna-XM_016585417.1 | LOC107766605 | mRNA | 973 | 451 | -1.018 | LOB domain-containing protein 20-like |
| 17 | rna-XM_016634901.1 | LOC107810163 | mRNA | 834 | 378 | -1.051 | probable fructokinase-5 |
| 18 | rna-XM_016625045.1 | LOC107801681 | mRNA | 754 | 341 | -1.054 | expansin-like A2 |
| 19 | rna-XM_016656094.1 | LOC107828729 | mRNA | 1868 | 834 | -1.072 | O-acyltransferase WSD1-like |
| 20 | rna-XM_016649299.1 | LOC107822731 | mRNA | 486 | 212 | -1.106 | protein EXORDIUM-like |
| 21 | rna-XM_016605912.1 | LOC107784736 | mRNA | 1220 | 524 | -1.128 | O-acyltransferase WSD1-like |
| 22 | rna-XM_016630788.1 | LOC107806600 | mRNA | 934 | 399 | -1.136 | glutelin type-B 2-like |
| 23 | rna-XM_016604448.1 | LOC107783475 | mRNA | 1295 | 553 | -1.136 | ABC transporter G family member 26-like |
| 24 | rna-XM_016590749.1 | LOC107771395 | mRNA | 712 | 304 | -1.137 | ornithine decarboxylase-like |
| 25 | rna-XM_016607864.1 | LOC107786397 | mRNA | 657 | 280 | -1.139 | lysosomal beta glucosidase-like |
| 26 | rna-XM_016636021.1 | LOC107811142 | mRNA | 536 | 224 | -1.168 | subtilisin-like protease SBT5.4 |
| 27 | rna-XM_016615122.1 | LOC107792874 | mRNA | 506 | 211 | -1.171 | fatty acid amide hydrolase-like, transcript variant X1 |
| 28 | rna-XM_016615123.1 | LOC107792874 | mRNA | 504 | 210 | -1.172 | fatty acid amide hydrolase-like, transcript variant X2 |
| 29 | rna-XM_016611289.1 | LOC107789470 | mRNA | 522 | 213 | -1.202 | ornithine decarboxylase-like |
| 30 | rna-XM_016618724.1 | LOC107796014 | mRNA | 714 | 283 | -1.244 | probable leucine-rich repeat receptor-like protein kinase At1g68400, transcript variant X1 |
| 31 | rna-XM_016603966.1 | LOC107783011 | mRNA | 1416 | 558 | -1.252 | beta-glucosidase BoGH3B-like |
| 32 | rna-XM_016636226.1 | LOC107811319 | mRNA | 334 | 131 | -1.259 | sugar carrier protein C-like |
| 33 | rna-XM_016585396.1 | LOC107766587 | mRNA | 976 | 380 | -1.270 | sugar transport protein 14-like |
| 34 | rna-XM_016648613.1 | LOC107822110 | mRNA | 695 | 269 | -1.278 | beta-galactosidase 15-like |
| 35 | rna-XM_016638896.1 | LOC107813610 | mRNA | 312 | 120 | -1.287 | 60S acidic ribosomal protein P0-like |
| 36 | rna-XM_016618726.1 | LOC107796014 | mRNA | 592 | 226 | -1.298 | probable leucine-rich repeat receptor-like protein kinase At1g68400, transcript variant X3 |
| 37 | rna-XM_016618725.1 | LOC107796014 | mRNA | 690 | 263 | -1.300 | probable leucine-rich repeat receptor-like protein kinase At1g68400, transcript variant X2 |
| 38 | rna-XM_016586939.1 | LOC107767841 | mRNA | 593 | 219 | -1.346 | probable pectinesterase 68 |
| 39 | rna-XM_016616836.1 | LOC107794356 | mRNA | 386 | 142 | -1.352 | serine/threonine-protein kinase tricorner-like |
| 40 | rna-XM_016621227.1 | LOC107798256 | mRNA | 1611 | 589 | -1.360 | sugar carrier protein C-like |
| 41 | rna-XM_016641190.1 | LOC107815580 | mRNA | 422 | 154 | -1.363 | bidirectional sugar transporter SWEET6a-like |
| 42 | rna-XM_016586497.1 | LOC107767486 | mRNA | 270 | 97 | -1.386 | ABC transporter G family member 26-like |
| 43 | rna-XM_016630367.1 | LOC107806241 | mRNA | 2188 | 778 | -1.401 | beta-galactosidase 15-like |
| 44 | gene-LOC107798170 | LOC107798170 | exon | 644 | 226 | -1.420 |  |
| 45 | rna-XM_016621229.1 | LOC107798257 | mRNA | 455 | 159 | -1.426 | BAG family molecular chaperone regulator 3-like |
| 46 | rna-XM_016641454.1 | LOC107815817 | mRNA | 306 | 106 | -1.438 | remorin-like |
| 47 | rna-XM_016605567.1 | LOC107784434 | mRNA | 396 | 134 | -1.472 | endochitinase 3 |
| 48 | rna-XM_016609741.1 | LOC107788085 | mRNA | 1324 | 446 | -1.479 | aldose 1-epimerase-like |
| 49 | rna-XM_016583300.1 | LOC107764698 | mRNA | 229 | 77 | -1.481 | protein SULFUR DEFICIENCY-INDUCED 1-like |
| 50 | rna-XM_016654250.1 | LOC107827166 | mRNA | 4025 | 1349 | -1.486 | spermidine hydroxycinnamoyl transferase-like, transcript variant X2 |
| 51 | rna-XM_016654249.1 | LOC107827166 | mRNA | 4025 | 1349 | -1.486 | spermidine hydroxycinnamoyl transferase-like, transcript variant X1 |
| 52 | rna-XM_016610227.1 | LOC107788550 | mRNA | 1513 | 506 | -1.489 | spermidine hydroxycinnamoyl transferase-like |
| 53 | rna-XM_016600920.1 | LOC107780391 | mRNA | 449 | 149 | -1.500 | endochitinase 3-like |
| 54 | rna-XM_016650869.1 | LOC107824131 | mRNA | 2311 | 749 | -1.534 | beta-xylosidase/alpha-L-arabinofuranosidase 1-like |
| 55 | rna-XM_016656495.1 | LOC107829067 | mRNA | 1019 | 312 | -1.616 | cinnamoyl-CoA reductase 1-like |
| 56 | rna-XM_016648351.1 | LOC107821894 | mRNA | 332 | 99 | -1.655 | uncharacterized LOC107821894 |
| 57 | rna-XM_016657099.1 | LOC107829645 | mRNA | 311 | 91 | -1.682 | aldose 1-epimerase-like |
| 58 | rna-XM_016616812.1 | LOC107794333 | mRNA | 603 | 175 | -1.694 | cinnamoyl-CoA reductase 2-like |
| 59 | rna-XM_016603160.1 | LOC107782292 | mRNA | 214 | 61 | -1.720 | xyloglucan endotransglucosylase/hydrolase protein 24-like |
| 60 | rna-XM_016635858.1 | LOC107811019 | mRNA | 356 | 101 | -1.726 | fatty acid amide hydrolase-like |
| 61 | rna-XR_001652067.1 | LOC107803745 | misc_RNA | 648 | 183 | -1.733 | **auxin response factor 3-like, transcript variant X1** |
| 62 | rna-XR_001652068.1 | LOC107803745 | misc_RNA | 648 | 183 | -1.733 | **auxin response factor 3-like, transcript variant X3** |
| 63 | rna-XM_016627510.1 | LOC107803745 | mRNA | 648 | 183 | -1.733 | **auxin response factor 3-like, transcript variant X2** |
| 64 | rna-XM_016632979.1 | LOC107808450 | mRNA | 1470 | 412 | -1.744 | non-specific lipid-transfer protein 13-like, transcript variant X2 |
| 65 | rna-XM_016632978.1 | LOC107808450 | mRNA | 1470 | 412 | -1.744 | non-specific lipid-transfer protein 13-like, transcript variant X1 |
| 66 | rna-XM_016632980.1 | LOC107808450 | mRNA | 1469 | 411 | -1.746 | non-specific lipid-transfer protein 13-like, transcript variant X3 |
| 67 | rna-XR_001653136.1 | LOC107808450 | misc_RNA | 1176 | 325 | -1.764 | non-specific lipid-transfer protein 13-like, transcript variant X4 |
| 68 | rna-XR_001647814.1 | LOC107785172 | misc_RNA | 377 | 102 | -1.795 | **auxin response factor 3-like, transcript variant X1** |
| 69 | rna-XR_001647815.1 | LOC107785172 | misc_RNA | 377 | 102 | -1.795 | **auxin response factor 3-like, transcript variant X3** |
| 70 | rna-XM_016606410.1 | LOC107785172 | mRNA | 377 | 102 | -1.795 | **auxin response factor 3-like, transcript variant X2** |
| 71 | rna-XM_016656261.1 | LOC107828874 | mRNA | 174 | 47 | -1.797 | protein DETOXIFICATION 33-like |
| 72 | rna-XM_016628192.1 | LOC107804318 | mRNA | 804 | 217 | -1.798 | probable beta-D-xylosidase 5 |
| 73 | rna-XM_016639259.1 | LOC107813934 | mRNA | 809 | 215 | -1.821 | probable xyloglucan endotransglucosylase/hydrolase protein 23 |
| 74 | rna-XM_016597020.1 | LOC107777056 | mRNA | 1021 | 268 | -1.839 | S-type anion channel SLAH2-like, transcript variant X3 |
| 75 | rna-XM_016597019.1 | LOC107777056 | mRNA | 1021 | 268 | -1.839 | S-type anion channel SLAH2-like, transcript variant X2 |
| 76 | rna-XM_016597018.1 | LOC107777056 | mRNA | 1022 | 268 | -1.840 | S-type anion channel SLAH2-like, transcript variant X1 |
| 77 | rna-XM_016649224.1 | LOC107822668 | mRNA | 658 | 169 | -1.870 | uncharacterized LOC107822668 |
| 78 | rna-XM_016600018.1 | LOC107779568 | mRNA | 1072 | 272 | -1.887 | caffeoylshikimate esterase-like |
| 79 | rna-XM_016584882.1 | LOC107766138 | mRNA | 317 | 79 | -1.913 | sugar carrier protein C-like |
| 80 | rna-XM_016597357.1 | LOC107777350 | mRNA | 1199 | 293 | -1.942 | protein HOTHEAD-like |
| 81 | rna-XM_016628947.1 | LOC107804979 | mRNA | 258 | 63 | -1.943 | sugar transport protein 12-like |
| 82 | rna-XM_016601040.1 | LOC107780491 | mRNA | 378 | 88 | -2.012 | caffeoylshikimate esterase-like |
| 83 | rna-XM_016634881.1 | LOC107810139 | mRNA | 194 | 45 | -2.017 | binding partner of ACD11 1-like, transcript variant X3 |
| 84 | rna-XM_016634880.1 | LOC107810139 | mRNA | 194 | 45 | -2.017 | binding partner of ACD11 1-like, transcript variant X2 |
| 85 | rna-XM_016634879.1 | LOC107810139 | mRNA | 194 | 45 | -2.017 | binding partner of ACD11 1-like, transcript variant X1 |
| 86 | rna-XM_016594643.1 | LOC107774983 | mRNA | 3360 | 769 | -2.036 | tetraketide alpha-pyrone reductase 1-like |
| 87 | rna-XM_016635928.1 | LOC107811073 | mRNA | 666 | 151 | -2.050 | protein HOTHEAD-like |
| 88 | rna-XM_016644978.1 | LOC107818905 | mRNA | 129 | 29 | -2.062 | putative F-box protein At1g65770 |
| 89 | rna-XM_016606813.1 | LOC107785490 | mRNA | 973 | 218 | -2.067 | **auxin response factor 4-like** |
| 90 | rna-XM_016580091.1 | LOC107761808 | mRNA | 429 | 93 | -2.115 | uncharacterized LOC107761808 |
| 91 | gene-LOC107793266 | LOC107793266 | exon | 140 | 30 | -2.131 |  |
| 92 | gene-LOC107799858 | LOC107799858 | exon | 4521 | 914 | -2.215 |  |
| 93 | rna-XM_016639945.1 | LOC107814514 | mRNA | 134 | 27 | -2.220 | S-type anion channel SLAH2-like, transcript variant X2 |
| 94 | rna-XM_016639944.1 | LOC107814514 | mRNA | 134 | 27 | -2.220 | S-type anion channel SLAH2-like, transcript variant X1 |
| 95 | rna-XM_016653169.1 | LOC107826218 | mRNA | 815 | 153 | -2.322 | **auxin response factor 4-like** |
| 96 | rna-XM_016613483.1 | LOC107791425 | mRNA | 268 | 48 | -2.390 | cytochrome P450 704B1-like |
| 97 | rna-XM_016635784.1 | LOC107810949 | mRNA | 169 | 30 | -2.403 | LOB domain-containing protein 6-like |
| 98 | rna-XM_016596307.1 | LOC107776414 | mRNA | 108 | 19 | -2.416 | cysteine proteinase RD21a-like |
| 99 | rna-XM_016634320.1 | LOC107809664 | mRNA | 1603 | 264 | -2.511 | cytochrome P450 704B1-like |
| 100 | rna-XM_016596931.1 | LOC107776975 | mRNA | 112 | 18 | -2.546 | abscisic acid 8'-hydroxylase 1-like |
| 101 | rna-XM_016643252.1 | LOC107817422 | mRNA | 104 | 15 | -2.702 | lysine histidine transporter-like 2 |
| 102 | rna-XM_016610673.1 | LOC107788932 | mRNA | 1082 | 116 | -3.130 | cytochrome P450 703A2-like |
| 103 | rna-XM_016615588.1 | LOC107793260 | mRNA | 474 | 48 | -3.213 | pathogenesis-related protein 5-like |
| 104 | rna-XM_016657724.1 | LOC107830227 | mRNA | 79 | 7 | -3.405 | histone deacetylase 6-like |
| 105 | rna-XM_016588444.1 | LOC107769241 | mRNA | 91 | 8 | -3.417 | RING-H2 finger protein ATL74-like |
| 106 | rna-XM_016639856.1 | LOC107814441 | mRNA | 448 | 36 | -3.546 | cytochrome P450 703A2-like |
| 107 | rna-XM_016617087.1 | LOC107794590 | mRNA | 5061 | 343 | -3.792 | fatty acyl-CoA reductase 2-like |
| 108 | rna-XM_016580156.1 | LOC107761866 | mRNA | 473 | 30 | -3.888 | WSC domain-containing protein ARB_07867-like |
| 109 | rna-XM_016641560.1 | LOC107815909 | mRNA | 81 | 5 | -3.927 | rhamnogalacturonate lyase-like |
| 110 | rna-XM_016586439.1 | LOC107767430 | mRNA | 65 | 4 | -3.931 | NAC domain-containing protein 68-like |
| 111 | rna-XM_016627488.1 | LOC107803725 | mRNA | 189 | 11 | -4.012 | WSC domain-containing protein ARB_07867-like |
| 112 | rna-XM_016584460.1 | LOC107765773 | mRNA | 2468 | 141 | -4.038 | fatty acyl-CoA reductase 2-like |
| 113 | rna-XM_016626267.1 | LOC107802698 | mRNA | 96 | 5 | -4.172 | pathogenesis-related protein 5-like |
| 114 | rna-XM_016640030.1 | LOC107814593 | mRNA | 174 | 9 | -4.182 | cytochrome b5-like |
| 115 | rna-XM_016589425.1 | LOC107770154 | mRNA | 55 | 2 | -4.690 | transcription factor MYB86-like |
| 116 | rna-XM_016643049.1 | LOC107817249 | mRNA | 111 | 3 | -5.118 | exopolygalacturonase-like |
| 117 | rna-XM_016652449.1 | LOC107825575 | mRNA | 85 | 2 | -5.318 | cation/H(+) antiporter 18-like, transcript variant X1 |
| 118 | rna-XM_016652456.1 | LOC107825575 | mRNA | 85 | 2 | -5.318 | cation/H(+) antiporter 18-like, transcript variant X2 |
| 125 | rna-XM_016641985.1 | LOC107816279 | mRNA | 49 | 0 | -5.524 | uncharacterized LOC107816279 |
| 119 | rna-XM_016578282.1 | LOC107760259 | mRNA | 56 | 1 | -5.716 | protein DETOXIFICATION 33-like |
| 126 | rna-XM_016579585.1 | LOC107761357 | mRNA | 58 | 0 | -5.767 | uncharacterized LOC107761357 |
| 120 | rna-XM_016627958.1 | LOC107804131 | mRNA | 70 | 1 | -6.038 | cation/H(+) antiporter 18-like |
| 121 | rna-XM_016633195.1 | LOC107808657 | mRNA | 102 | 1 | -6.581 | aquaporin-like, transcript variant X1 |
| 122 | rna-XM_016633197.1 | LOC107808657 | mRNA | 102 | 1 | -6.581 | aquaporin-like, transcript variant X3 |
| 123 | rna-XM_016633196.1 | LOC107808657 | mRNA | 102 | 1 | -6.581 | aquaporin-like, transcript variant X2 |
| 124 | rna-XM_016633778.1 | LOC107809188 | mRNA | 391 | 2 | -7.520 | aquaporin-1-like |

Table S15. The GO analysis of up-regulated genes in leaves (wild-type vs TAS3i plants)

| No | GO_term | GOid | All | DEG | P-value | Ontologies |
| --- | --- | --- | --- | --- | --- | --- |
| 1 | carbon utilization | GO:0015976 | 29 | 11 | 1.83E-24 | biological_process |
| 2 | carbonate dehydratase activity | GO:0004089 | 56 | 11 | 5.03E-22 | molecular_function |
| 3 | serine-type endopeptidase inhibitor activity | GO:0004867 | 44 | 8 | 2.25E-15 | molecular_function |
| 4 | negative regulation of endopeptidase activity | GO:0010951 | 174 | 10 | 4.04E-15 | biological_process |
| 5 | lyase activity | GO:0016829 | 671 | 13 | 2.40E-14 | molecular_function |
| 6 | killing of cells of other organism | GO:0031640 | 46 | 3 | 0.001393 | biological_process |
| 7 | response to wounding | GO:0009611 | 134 | 3 | 0.023408 | biological_process |
| 8 | glucose-1-phosphate adenylyltransferase activity | GO:0008878 | 41 | 2 | 0.023408 | molecular_function |
| 9 | defense response to fungus | GO:0050832 | 175 | 3 | 0.044548 | biological_process |
| 10 | endopeptidase inhibitor activity | GO:0004866 | 31 | 2 | 0.051831 | molecular_function |
| 11 | glycogen biosynthetic process | GO:0005978 | 41 | 2 | 0.080336 | biological_process |
| 12 | intramolecular transferase activity | GO:0016866 | 43 | 2 | 0.080686 | molecular_function |
| 13 | zinc ion binding | GO:0008270 | 7543 | 12 | 0.086397 | molecular_function |
| 14 | starch biosynthetic process | GO:0019252 | 47 | 2 | 0.086397 | biological_process |
| 15 | vacuole | GO:0005773 | 384 | 3 | 0.25796 | cellular_component |
| 16 | transferase activity, transferring hexosyl groups | GO:0016758 | 399 | 3 | 0.269746 | molecular_function |
| 17 | terpene synthase activity | GO:0010333 | 142 | 2 | 0.556849 | molecular_function |
| 18 | myrcene synthase activity | GO:0050551 | 4 | 1 | 0.556849 | molecular_function |
| 19 | (4S)-limonene synthase activity | GO:0050552 | 4 | 1 | 0.556849 | molecular_function |
| 20 | cell wall | GO:0005618 | 559 | 3 | 0.55932 | cellular_component |

Table S16. The GO analysis of down-regulated genes in leaves (wild-type vs TAS3i plants)

| No | GO_term | GOid | All | DEG | P-value | Ontologies |
| --- | --- | --- | --- | --- | --- | --- |
| 1 | vegetative phase change | GO:0010050 | 8 | 6 | 2.66E-15 | biological_process |
| 2 | abaxial cell fate specification | GO:0010158 | 10 | 6 | 3.55E-15 | biological_process |
| 3 | response to hormone | GO:0009725 | 148 | 8 | 1.10E-13 | biological_process |
| 4 | **auxin-activated signaling pathway** | GO:0009734 | 326 | 8 | 3.87E-11 | biological_process |
| 5 | floral meristem determinacy | GO:0010582 | 22 | 4 | 4.77E-08 | biological_process |
| 6 | transcription, DNA-templated | GO:0006351 | 2881 | 8 | 0.000522 | biological_process |
| 7 | regulation of transcription, DNA-templated | GO:0006355 | 4728 | 8 | 0.016193 | biological_process |
| 8 | DNA binding | GO:0003677 | 6192 | 8 | 0.091326 | molecular_function |
| 9 | nucleus | GO:0005634 | 7597 | 8 | 0.314414 | cellular_component |
| 10 | transcription factor activity, sequence-specific DNA binding | GO:0003700 | 2085 | 4 | 0.855039 | molecular_function |

Table S17. The GO analysis of up-regulated genes in flowers (wild-type vs TAS3i plants)

| No | GO_term | GOid | All | DEG | P-value | Ontologies |
| --- | --- | --- | --- | --- | --- | --- |
| 1 | adenylate cyclase activity | GO:0004016 | 5 | 2 | 0.000696 | molecular_function |
| 2 | cAMP biosynthetic process | GO:0006171 | 5 | 2 | 0.000696 | biological_process |

Table S18. The GO analysis of down-regulated genes in flowers (wild-type vs TAS3i plants)

| No | GO_term | GOid | All | DEG | P-value | Ontologies |
| --- | --- | --- | --- | --- | --- | --- |
| 1 | chitin binding | GO:0008061 | 30 | 6 | 2.67E-09 | molecular_function |
| 2 | response to biotic stimulus | GO:0009607 | 158 | 7 | 2.95E-07 | biological_process |
| 3 | defense response | GO:0006952 | 1665 | 14 | 3.61E-07 | biological_process |
| 4 | cell wall macromolecule catabolic process | GO:0016998 | 25 | 4 | 1.22E-05 | biological_process |
| 5 | hydrolase activity, acting on glycosyl bonds | GO:0016798 | 739 | 9 | 1.68E-05 | molecular_function |
| 6 | carbohydrate metabolic process | GO:0005975 | 1391 | 11 | 2.86E-05 | biological_process |
| 7 | vacuole | GO:0005773 | 384 | 7 | 3.25E-05 | cellular_component |
| 8 | apoplast | GO:0048046 | 279 | 6 | 0.000102 | cellular_component |
| 9 | chitinase activity | GO:0004568 | 60 | 4 | 0.000128 | molecular_function |
| 10 | chitin catabolic process | GO:0006032 | 59 | 4 | 0.000128 | biological_process |
| 11 | hydroquinone:oxygen oxidoreductase activity | GO:0052716 | 72 | 4 | 0.000213 | molecular_function |
| 12 | lignin catabolic process | GO:0046274 | 72 | 4 | 0.000213 | biological_process |
| 13 | polysaccharide catabolic process | GO:0000272 | 88 | 4 | 0.000425 | biological_process |
| 14 | licheninase activity | GO:0042972 | 2 | 2 | 0.001323 | molecular_function |
| 15 | 1-aminocyclopropane-1-carboxylate oxidase activity | GO:0009815 | 3 | 2 | 0.002057 | molecular_function |
| 16 | hydrolase activity | GO:0016787 | 5182 | 16 | 0.002713 | molecular_function |
| 17 | seed coat development | GO:0010214 | 10 | 2 | 0.011931 | biological_process |
| 18 | ethylene biosynthetic process | GO:0009693 | 11 | 2 | 0.01331 | biological_process |
| 19 | metabolic process | GO:0008152 | 3072 | 11 | 0.015802 | biological_process |
| 20 | indole-3-acetic acid amido synthetase activity | GO:0010279 | 13 | 2 | 0.016108 | molecular_function |
| 21 | pigment biosynthetic process | GO:0046148 | 16 | 2 | 0.020387 | biological_process |
| 22 | catechol oxidase activity | GO:0004097 | 16 | 2 | 0.020387 | molecular_function |
| 23 | hydrolase activity, hydrolyzing O-glycosyl compounds | GO:0004553 | 869 | 6 | 0.020387 | molecular_function |
| 24 | alpha-L-arabinofuranosidase activity | GO:0046556 | 19 | 2 | 0.026758 | molecular_function |
| 25 | copper ion binding | GO:0005507 | 314 | 4 | 0.028519 | molecular_function |
| 26 | **auxin homeostasis** | GO:0010252 | 27 | 2 | 0.047543 | biological_process |
| 27 | L-ascorbic acid binding | GO:0031418 | 48 | 2 | 0.136554 | molecular_function |
| 28 | oxidoreductase activity, oxidizing metal ions | GO:0016722 | 49 | 2 | 0.136977 | molecular_function |
| 29 | oxidoreductase activity | GO:0016491 | 3755 | 10 | 0.212758 | molecular_function |
| 30 | unidimensional cell growth | GO:0009826 | 79 | 2 | 0.319585 | biological_process |
| 31 | defense response to bacterium | GO:0042742 | 107 | 2 | 0.504498 | biological_process |
| 32 | nucleic acid binding | GO:0003676 | 10114 | 1 | 0.534134 | molecular_function |
| 33 | pectinesterase activity | GO:0030599 | 154 | 2 | 0.880553 | molecular_function |
| 34 | aspartyl esterase activity | GO:0045330 | 152 | 2 | 0.880553 | molecular_function |
| 35 | cell wall modification | GO:0042545 | 152 | 2 | 0.880553 | biological_process |
| 36 | extracellular region | GO:0005576 | 909 | 4 | 0.880553 | cellular_component |
| 37 | oxidation-reduction process | GO:0055114 | 4882 | 10 | 0.950823 | biological_process |

Table S19. The GO analysis of up-regulated genes in buds (wild-type vs TAS3i plants)

| No | GO_term | GOid | All | DEG | P-value | Ontologies |
| --- | --- | --- | --- | --- | --- | --- |
| 1 | pollen exine formation | GO:0010584 | 19 | 4 | 1.63E-05 | biological_process |
| 2 | cell wall macromolecule catabolic process | GO:0016998 | 25 | 4 | 2.18E-05 | biological_process |
| 3 | chitin binding | GO:0008061 | 30 | 4 | 2.83E-05 | molecular_function |
| 4 | sporopollenin biosynthetic process | GO:0080110 | 9 | 3 | 0.000128 | biological_process |
| 5 | chitin catabolic process | GO:0006032 | 59 | 4 | 0.00019 | biological_process |
| 6 | chitinase activity | GO:0004568 | 60 | 4 | 0.00019 | molecular_function |
| 7 | negative regulation of transposition | GO:0010529 | 2 | 2 | 0.001749 | biological_process |
| 8 | polyketide biosynthetic process | GO:0030639 | 2 | 2 | 0.001749 | biological_process |
| 9 | seed trichome initiation | GO:0090377 | 2 | 2 | 0.001749 | biological_process |
| 10 | tetraketide alpha-pyrone synthase activity | GO:0090439 | 2 | 2 | 0.001749 | molecular_function |
| 11 | metabolic process | GO:0008152 | 3072 | 12 | 0.004171 | biological_process |
| 12 | adenylate cyclase activity | GO:0004016 | 5 | 2 | 0.004702 | molecular_function |
| 13 | cAMP biosynthetic process | GO:0006171 | 5 | 2 | 0.004702 | biological_process |
| 14 | chromatin DNA binding | GO:0031490 | 6 | 2 | 0.005818 | molecular_function |
| 15 | pollen wall assembly | GO:0010208 | 7 | 2 | 0.006978 | biological_process |
| 16 | positive regulation of histone H3-K9 dimethylation | GO:1900111 | 8 | 2 | 0.008173 | biological_process |
| 17 | hydrolase activity, acting on glycosyl bonds | GO:0016798 | 739 | 6 | 0.009783 | molecular_function |
| 18 | negative regulation of histone acetylation | GO:0035067 | 12 | 2 | 0.01466 | biological_process |
| 19 | extracellular region | GO:0005576 | 909 | 6 | 0.02691 | cellular_component |
| 20 | carbohydrate metabolic process | GO:0005975 | 1391 | 7 | 0.034814 | biological_process |
| 21 | strictosidine synthase activity | GO:0016844 | 21 | 2 | 0.034814 | molecular_function |
| 22 | S-adenosylmethionine-dependent methyltransferase activity | GO:0008757 | 138 | 3 | 0.044752 | molecular_function |
| 23 | pollen development | GO:0009555 | 157 | 3 | 0.062002 | biological_process |
| 24 | mannan endo-1,4-beta-mannosidase activity | GO:0016985 | 32 | 2 | 0.064378 | molecular_function |
| 25 | mannan catabolic process | GO:0046355 | 32 | 2 | 0.064378 | biological_process |
| 26 | biosynthetic process | GO:0009058 | 415 | 4 | 0.067275 | biological_process |
| 27 | lignin biosynthetic process | GO:0009809 | 34 | 2 | 0.067275 | biological_process |
| 28 | peptidase activity | GO:0008233 | 934 | 5 | 0.172993 | molecular_function |
| 29 | hydrolase activity | GO:0016787 | 5182 | 12 | 0.183157 | molecular_function |
| 30 | trans-Golgi network | GO:0005802 | 252 | 3 | 0.183157 | cellular_component |
| 31 | vacuolar membrane | GO:0005774 | 260 | 3 | 0.193609 | cellular_component |
| 32 | lignin catabolic process | GO:0046274 | 72 | 2 | 0.223107 | biological_process |
| 33 | hydroquinone:oxygen oxidoreductase activity | GO:0052716 | 72 | 2 | 0.223107 | molecular_function |
| 34 | salutaridinol 7-O-acetyltransferase activity | GO:0047180 | 1 | 1 | 0.223107 | molecular_function |
| 35 | vegetative to reproductive phase transition of meristem | GO:0010228 | 75 | 2 | 0.234413 | biological_process |
| 36 | transferase activity, transferring acyl groups other than amino-acyl groups | GO:0016747 | 306 | 3 | 0.263573 | molecular_function |
| 37 | endosome | GO:0005768 | 319 | 3 | 0.280545 | cellular_component |
| 38 | endoplasmic reticulum | GO:0005783 | 759 | 4 | 0.415474 | cellular_component |
| 39 | nucleic acid binding | GO:0003676 | 10114 | 1 | 0.451371 | molecular_function |
| 40 | transferase activity, transferring acyl groups | GO:0016746 | 440 | 3 | 0.616915 | molecular_function |
| 41 | anther wall tapetum development | GO:0048658 | 6 | 1 | 0.616915 | biological_process |
| 42 | lipid catabolic process | GO:0016042 | 151 | 2 | 0.728297 | biological_process |
| 43 | caffeoyl-CoA O-methyltransferase activity | GO:0042409 | 10 | 1 | 0.903773 | molecular_function |

Table S20. The GO analysis of down-regulated genes in buds (wild-type vs TAS3i plants)

| No | GO_term | GOid | All | DEG | P-value | Ontologies |
| --- | --- | --- | --- | --- | --- | --- |
| 1 | anther wall tapetum cell differentiation | GO:0048657 | 6 | 6 | 9.24E-11 | biological_process |
| 2 | anther morphogenesis | GO:0048654 | 6 | 6 | 9.24E-11 | biological_process |
| 3 | anther development | GO:0048653 | 8 | 6 | 1.50E-10 | biological_process |
| 4 | vegetative phase change | GO:0010050 | 8 | 6 | 1.50E-10 | biological_process |
| 5 | abaxial cell fate specification | GO:0010158 | 10 | 6 | 3.18E-10 | biological_process |
| 6 | pollen exine formation | GO:0010584 | 19 | 6 | 5.78E-09 | biological_process |
| 7 | cellular ion homeostasis | GO:0006873 | 15 | 5 | 2.33E-07 | biological_process |
| 8 | response to hormone | GO:0009725 | 148 | 8 | 4.93E-07 | biological_process |
| 9 | voltage-gated anion channel activity | GO:0008308 | 19 | 5 | 4.93E-07 | molecular_function |
| 10 | carbohydrate metabolic process | GO:0005975 | 1391 | 16 | 9.38E-06 | biological_process |
| 11 | regulation of anion transmembrane transport | GO:1903959 | 49 | 5 | 2.86E-05 | biological_process |
| 12 | spermidine hydroxycinnamate conjugate biosynthetic process | GO:0080088 | 3 | 3 | 3.78E-05 | biological_process |
| 13 | spermidine:feruloyl CoA N-acyltransferase activity | GO:0080075 | 3 | 3 | 3.78E-05 | molecular_function |
| 14 | spermidine:caffeoyl CoA N-acyltransferase activity | GO:0080074 | 3 | 3 | 3.78E-05 | molecular_function |
| 15 | spermidine:coumaroyl CoA N-acyltransferase activity | GO:0080073 | 3 | 3 | 3.78E-05 | molecular_function |
| 16 | spermidine:sinapoyl CoA N-acyltransferase activity | GO:0080072 | 3 | 3 | 3.78E-05 | molecular_function |
| 17 | floral meristem determinacy | GO:0010582 | 22 | 4 | 4.86E-05 | biological_process |
| 18 | **auxin-activated signaling pathway** | GO:0009734 | 326 | 8 | 8.10E-05 | biological_process |
| 19 | N-acyltransferase activity | GO:0016410 | 5 | 3 | 8.88E-05 | molecular_function |
| 20 | transmembrane transport | GO:0055085 | 2027 | 17 | 0.000137931 | biological_process |
| 21 | pollen development | GO:0009555 | 157 | 6 | 0.000177394 | biological_process |
| 22 | monovalent cation:proton antiporter activity | GO:0005451 | 10 | 3 | 0.000388984 | molecular_function |
| 23 | hydrolase activity, acting on glycosyl bonds | GO:0016798 | 739 | 10 | 0.000445286 | molecular_function |
| 24 | hydrolase activity, hydrolyzing O-glycosyl compounds | GO:0004553 | 869 | 10 | 0.001715521 | molecular_function |
| 25 | nucleic acid binding | GO:0003676 | 10114 | 3 | 0.002116779 | molecular_function |
| 26 | substrate-specific transmembrane transporter activity | GO:0022891 | 169 | 5 | 0.003984633 | molecular_function |
| 27 | regulation of pH | GO:0006885 | 38 | 3 | 0.01135604 | biological_process |
| 28 | ornithine decarboxylase activity | GO:0004586 | 5 | 2 | 0.01198827 | molecular_function |
| 29 | transporter activity | GO:0005215 | 908 | 9 | 0.01219219 | molecular_function |
| 30 | sporopollenin biosynthetic process | GO:0080110 | 9 | 2 | 0.02915766 | biological_process |
| 31 | galactose oxidase activity | GO:0045480 | 13 | 2 | 0.05359891 | molecular_function |
| 32 | pollen maturation | GO:0010152 | 15 | 2 | 0.06708514 | biological_process |
| 33 | glucan catabolic process | GO:0009251 | 16 | 2 | 0.07223551 | biological_process |
| 34 | cell | GO:0005623 | 345 | 5 | 0.07642786 | cellular_component |
| 35 | transmembrane transporter activity | GO:0022857 | 355 | 5 | 0.08430284 | molecular_function |
| 36 | hexose metabolic process | GO:0019318 | 19 | 2 | 0.08679453 | biological_process |
| 37 | aldose 1-epimerase activity | GO:0004034 | 19 | 2 | 0.08679453 | molecular_function |
| 38 | carbohydrate binding | GO:0030246 | 373 | 5 | 0.0967304 | molecular_function |
| 39 | 3-beta-hydroxy-delta5-steroid dehydrogenase activity | GO:0003854 | 24 | 2 | 0.1268108 | molecular_function |
| 40 | cell wall macromolecule catabolic process | GO:0016998 | 25 | 2 | 0.1334478 | biological_process |
| 41 | fatty-acyl-CoA reductase (alcohol-forming) activity | GO:0080019 | 26 | 2 | 0.1401155 | molecular_function |
| 42 | chitin binding | GO:0008061 | 30 | 2 | 0.1786816 | molecular_function |
| 43 | carbon-nitrogen ligase activity, with glutamine as amido-N-donor | GO:0016884 | 125 | 3 | 0.1931251 | molecular_function |
| 44 | polyamine biosynthetic process | GO:0006596 | 32 | 2 | 0.1931251 | biological_process |
| 45 | glycerolipid biosynthetic process | GO:0045017 | 34 | 2 | 0.2110929 | biological_process |
| 46 | zinc ion binding | GO:0008270 | 7543 | 4 | 0.2714599 | molecular_function |
| 47 | solute:proton antiporter activity | GO:0015299 | 145 | 3 | 0.2730441 | molecular_function |
| 48 | lipase activity | GO:0016298 | 44 | 2 | 0.3215157 | molecular_function |
| 49 | endomembrane system | GO:0012505 | 159 | 3 | 0.3377223 | cellular_component |
| 50 | transport | GO:0006810 | 2879 | 13 | 0.3567959 | biological_process |
| 51 | diacylglycerol O-acyltransferase activity | GO:0004144 | 48 | 2 | 0.3567959 | molecular_function |
| 52 | steroid biosynthetic process | GO:0006694 | 49 | 2 | 0.3638974 | biological_process |
| 53 | cell wall | GO:0005618 | 559 | 5 | 0.3918993 | cellular_component |
| 54 | chitin catabolic process | GO:0006032 | 59 | 2 | 0.4970645 | biological_process |
| 55 | chitinase activity | GO:0004568 | 60 | 2 | 0.5038326 | molecular_function |
| 56 | vacuole | GO:0005773 | 384 | 4 | 0.5267091 | cellular_component |
| 57 | beta-galactosidase activity | GO:0004565 | 66 | 2 | 0.5816491 | molecular_function |
| 58 | cell wall organization | GO:0071555 | 403 | 4 | 0.599584 | biological_process |
| 59 | extracellular region | GO:0005576 | 909 | 6 | 0.6340551 | cellular_component |
| 60 | beta-glucosidase activity | GO:0008422 | 71 | 2 | 0.6340551 | molecular_function |
| 61 | hydrogen ion transmembrane transport | GO:1902600 | 223 | 3 | 0.6523393 | biological_process |
| 62 | regulation of defense response to fungus | GO:1900150 | 4 | 1 | 0.6523393 | biological_process |
| 63 | alkane 1-monooxygenase activity | GO:0018685 | 4 | 1 | 0.6523393 | molecular_function |
| 64 | maintenance of meristem identity | GO:0010074 | 4 | 1 | 0.6523393 | biological_process |
| 65 | monooxygenase activity | GO:0004497 | 703 | 5 | 0.8060048 | molecular_function |
| 66 | ATP binding | GO:0005524 | 8057 | 6 | 0.8512816 | molecular_function |
| 67 | polysaccharide catabolic process | GO:0000272 | 88 | 2 | 0.8512816 | biological_process |
| 68 | regulation of sulfur utilization | GO:0006792 | 6 | 1 | 0.8587086 | biological_process |
| 69 | oxidoreductase activity, acting on paired donors, with incorporation or reduction of molecular oxygen | GO:0016705 | 737 | 5 | 0.9132508 | molecular_function |

Table S21. The top ten gene ontology terms among the differentially expressed genes^†^ (*rdr6* vs. TAS3i plants)

|  | GO_term^‡^ (All, DEGs^§^) | GO_term (All, DEGs) | GO_term (All, DEGs) |
| --- | --- | --- | --- |
|  | Leaves | Flowers | Buds |
| UP | carbon utilization (29, 10)  carbonate dehydratase activity (56, 10)  lyase activity (671, 16)  negative regulation of endopeptidase activity (174, 7)  serine-type endopeptidase inhibitor activity (44, 5)  glucose-1-phosphate adenylyltransferase activity (18, 4)  glycogen biosynthetic process (41, 4)  starch biosynthetic process (47, 4)  killing of cells of other organism (46, 3)  adenylate cyclase activity (5, 2)  cAMP biosynthetic process (5, 2) | photosynthesis, light harvesting (90, 13)  chlorophyll-binding (117, 13)  protein-chromophore linkage (121, 13)  photosystem I (146, 13)  photosystem II (175, 13)  photosynthesis (284, 14)  thylakoid (319, 13)  chloroplast thylakoid membrane (464, 13)  plastid (827, 14)  chloroplast (2576, 16) | adenylate cyclase activity (5, 2)  cAMP biosynthetic process (5, 2)  integral component of membrane (22126, 7)  membrane (22913, 7)  extracellular region (909, 3)  transmembrane transport (2027, 3)  hydrolase activity (5182, 3)  photosystem II (175, 2)  photosynthesis (284, 2)  thylakoid (319, 2) |
| DOWN | vegetative phase change (8, 6)  abaxial cell fate specification (10, 6)  response to hormones (148, 8)  **auxin-activated signaling pathway (326, 8)**  floral meristem determinacy (22, 4)  transcription, DNA-templated (2881, 8)  DNA binding (6192, 9)  regulation of transcription, DNA-templated (4728, 8)  plant-type cell wall organization (138, 2)  nucleus (7597, 8) | vegetative phase change (8, 6)  abaxial cell fate specification (10, 6)  response to hormones (148, 8)  **auxin-activated signaling pathway (326, 9)**  floral meristem determinacy (22, 4)  transcription, DNA-templated (2881, 8)  extracellular region (909, 4)  xyloglucan metabolic process (100, 2)  cellular glucan metabolic process (101, 2)  xyloglucan:xyloglucosyl transferase activity (101, 2) | vegetative phase change (8, 6)  abaxial cell fate specification (10, 6)  response to hormones (148, 8)  pectin catabolic process (212, 8)  **auxin-activated signaling pathway (326, 8)**  floral meristem determinacy (22, 4)  transporter activity (908, 11)  extracellular space (90, 5)  cell wall modification (152, 5)  aspartyl esterase activity (152, 5)  pectinesterase activity (154, 5) |

^†^Sorted by p-values; ^‡^ GO – gene ontology; ^§^ DEGs – differentially expressed genes

Table S22. The differentially expressed genes in leaves of *rdr6* plants vs TAS3i plants

|  |  |  |  | expression amounts in leaves of | |  |  |
| --- | --- | --- | --- | --- | --- | --- | --- |
|  | Gene_ID | Gene | Gbkey | rdr6 plants | TAS3i plants | m.value [log2(expression ratio)] | Product |
| 1 | rna-XM_016641299.1 | LOC107815675 | mRNA | 0 | 202 | 7.410 | zinc finger BED domain-containing protein RICESLEEPER 3-like |
| 2 | rna-XM_016613856.1 | LOC107791735 | mRNA | 1 | 129 | 6.763 | defensin-like protein |
| 3 | rna-XM_016659003.1 | LOC107831250 | mRNA | 1 | 80 | 6.074 | (-)-alpha-terpineol synthase-like |
| 4 | rna-XM_016659547.1 | LOC107831753 | mRNA | 9 | 322 | 4.913 | defensin-like protein |
| 5 | rna-XM_016659546.1 | LOC107831752 | mRNA | 4 | 116 | 4.610 | defensin-like protein |
| 6 | rna-XM_016621555.1 | LOC107798543 | mRNA | 3 | 73 | 4.357 | (-)-camphene/tricyclene synthase, chloroplastic-like |
| 7 | rna-XM_016598669.1 | LOC107778417 | mRNA | 7 | 100 | 3.589 | glucose-6-phosphate/phosphate translocator 2, chloroplastic |
| 8 | rna-XM_016631900.1 | LOC107807494 | mRNA | 21 | 251 | 3.331 | pelargonidin 3-O-(6-caffeoylglucoside) 5-O-(6-O-malonylglucoside) 4'''-malonyltransferase-like |
| 9 | rna-XM_016655118.1 | LOC107827891 | mRNA | 392 | 3486 | 2.905 | proteinase inhibitor I-B-like |
| 10 | rna-XM_016618457.1 | LOC107795773 | mRNA | 447 | 3740 | 2.817 | uncharacterized LOC107795773 |
| 11 | rna-XM_016637536.1 | LOC107812321 | mRNA | 358 | 2506 | 2.559 | carbonic anhydrase, chloroplastic-like, transcript variant X3 |
| 12 | rna-XM_016596315.1 | LOC107776420 | mRNA | 29 | 199 | 2.531 | glucose-6-phosphate/phosphate translocator 2, chloroplastic-like |
| 13 | rna-XM_016612493.1 | LOC107790557 | mRNA | 268 | 1827 | 2.521 | proteinase inhibitor I-B-like |
| 14 | rna-XM_016637608.1 | LOC107812321 | mRNA | 443 | 2831 | 2.428 | carbonic anhydrase, chloroplastic-like, transcript variant X4 |
| 15 | rna-XM_016637681.1 | LOC107812321 | mRNA | 443 | 2831 | 2.428 | carbonic anhydrase, chloroplastic-like, transcript variant X5 |
| 16 | rna-XM_016637404.1 | LOC107812321 | mRNA | 443 | 2831 | 2.428 | carbonic anhydrase, chloroplastic-like, transcript variant X1 |
| 17 | rna-XM_016637478.1 | LOC107812321 | mRNA | 443 | 2831 | 2.428 | carbonic anhydrase, chloroplastic-like, transcript variant X2 |
| 18 | rna-XM_016614664.1 | LOC107792454 | mRNA | 434 | 2773 | 2.428 | wound-induced proteinase inhibitor 2-like |
| 19 | rna-XM_016602129.1 | LOC107781430 | mRNA | 659 | 3974 | 2.344 | wound-induced proteinase inhibitor 2-like |
| 20 | rna-XM_016642073.1 | LOC107816366 | mRNA | 227 | 1341 | 2.315 | cysteine protease inhibitor 8-like |
| 21 | rna-XM_016623020.1 | LOC107799889 | mRNA | 68 | 369 | 2.192 | cysteine protease inhibitor 8-like |
| 22 | rna-XM_016655179.1 | LOC107827944 | mRNA | 772 | 3603 | 1.975 | putative UDP-rhamnose:rhamnosyltransferase 1 |
| 23 | rna-XM_016623090.1 | LOC107799946 | mRNA | 73 | 320 | 1.884 | protein phosphatase 1 regulatory subunit pprA-like, transcript variant X2 |
| 24 | rna-XM_016623089.1 | LOC107799946 | mRNA | 73 | 320 | 1.884 | protein phosphatase 1 regulatory subunit pprA-like, transcript variant X1 |
| 25 | rna-XM_016588188.1 | LOC107769016 | mRNA | 237 | 920 | 1.709 | beta carbonic anhydrase 6, mitochondrial-like, transcript variant X1 |
| 26 | rna-XM_016588189.1 | LOC107769016 | mRNA | 237 | 920 | 1.709 | beta carbonic anhydrase 6, mitochondrial-like, transcript variant X2 |
| 27 | rna-XM_016606330.1 | LOC107785105 | mRNA | 192 | 730 | 1.679 | GDSL esterase/lipase At2g38180-like |
| 28 | rna-XR_001650672.1 | LOC107797352 | ncRNA | 554 | 1994 | 1.600 | uncharacterized LOC107797352 |
| 29 | rna-XM_016628544.1 | LOC107804626 | mRNA | 156 | 555 | 1.583 | uncharacterized LOC107804626, transcript variant X1 |
| 30 | rna-XM_016623777.1 | LOC107800585 | mRNA | 334 | 1188 | 1.583 | isoflavone reductase homolog |
| 31 | rna-XM_016628546.1 | LOC107804626 | mRNA | 134 | 476 | 1.581 | uncharacterized LOC107804626, transcript variant X2 |
| 32 | rna-XM_016581980.1 | LOC107763496 | mRNA | 1474 | 5205 | 1.572 | glucose-6-phosphate/phosphate translocator 2, chloroplastic-like |
| 33 | rna-XM_016619110.1 | LOC107796351 | mRNA | 2467 | 8673 | 1.566 | proteinase inhibitor I-B-like |
| 34 | rna-XM_016650070.1 | LOC107823429 | mRNA | 262 | 918 | 1.561 | GDSL esterase/lipase At2g38180-like |
| 35 | gene-LOC107762192 | LOC107762192 | exon | 785 | 2737 | 1.554 |  |
| 36 | rna-XM_016657558.1 | LOC107830074 | mRNA | 752 | 2616 | 1.551 | uncharacterized LOC107830074, transcript variant X1 |
| 37 | rna-XM_016586888.1 | LOC107767796 | mRNA | 222 | 762 | 1.531 | carbonic anhydrase, chloroplastic-like, transcript variant X1 |
| 38 | rna-XM_016586889.1 | LOC107767796 | mRNA | 222 | 762 | 1.531 | carbonic anhydrase, chloroplastic-like, transcript variant X2 |
| 39 | rna-XM_016657559.1 | LOC107830074 | mRNA | 691 | 2359 | 1.523 | uncharacterized LOC107830074, transcript variant X2 |
| 40 | gene-LOC107798614 | LOC107798614 | exon | 423 | 1429 | 1.508 |  |
| 41 | rna-XM_016617806.1 | LOC107795210 | mRNA | 196 | 661 | 1.506 | alcohol dehydrogenase-like |
| 42 | rna-XM_016589864.1 | LOC107770515 | mRNA | 141 | 468 | 1.483 | beta-amyrin synthase-like, transcript variant X1 |
| 43 | rna-XM_016589922.1 | LOC107770515 | mRNA | 141 | 468 | 1.483 | beta-amyrin synthase-like, transcript variant X2 |
| 44 | rna-XM_016578154.1 | LOC107760136 | mRNA | 424 | 1377 | 1.451 | transmembrane protein 184C-like |
| 45 | rna-XM_016653552.1 | LOC107826565 | mRNA | 839 | 2681 | 1.428 | glucose-1-phosphate adenylyltransferase large subunit 1-like, transcript variant X1 |
| 46 | rna-XM_016653553.1 | LOC107826565 | mRNA | 837 | 2673 | 1.427 | glucose-1-phosphate adenylyltransferase large subunit 1-like, transcript variant X2 |
| 47 | rna-XM_016623650.1 | LOC107800476 | mRNA | 167 | 528 | 1.413 | alcohol dehydrogenase-like |
| 48 | rna-XM_016600714.1 | LOC107780184 | mRNA | 327 | 1021 | 1.395 | probable isoaspartyl peptidase/L-asparaginase 2 |
| 49 | rna-XM_016594876.1 | LOC107775179 | mRNA | 163 | 508 | 1.392 | transmembrane protein 184C-like, transcript variant X1 |
| 50 | rna-XM_016594880.1 | LOC107775179 | mRNA | 163 | 508 | 1.392 | transmembrane protein 184C-like, transcript variant X5 |
| 51 | rna-XM_016594879.1 | LOC107775179 | mRNA | 163 | 508 | 1.392 | transmembrane protein 184C-like, transcript variant X4 |
| 52 | rna-XM_016594877.1 | LOC107775179 | mRNA | 163 | 508 | 1.392 | transmembrane protein 184C-like, transcript variant X2 |
| 53 | rna-XM_016594878.1 | LOC107775179 | mRNA | 163 | 508 | 1.392 | transmembrane protein 184C-like, transcript variant X3 |
| 54 | rna-XM_016594881.1 | LOC107775179 | mRNA | 163 | 508 | 1.392 | transmembrane protein 184C-like, transcript variant X6 |
| 55 | rna-XM_016581069.1 | LOC107762694 | mRNA | 808 | 2413 | 1.330 | glucose-1-phosphate adenylyltransferase large subunit 1, transcript variant X1 |
| 56 | rna-XM_016581070.1 | LOC107762694 | mRNA | 808 | 2413 | 1.330 | glucose-1-phosphate adenylyltransferase large subunit 1, transcript variant X2 |
| 57 | rna-XM_016613293.1 | LOC107791262 | mRNA | 463 | 1288 | 1.228 | trans-cinnamate 4-monooxygenase-like |
| 58 | rna-XM_016653519.1 | LOC107826530 | mRNA | 1340 | 3531 | 1.150 | trans-cinnamate 4-monooxygenase |
| 59 | rna-XM_016608272.1 | LOC107786762 | mRNA | 1977 | 4925 | 1.069 | phenylalanine ammonia-lyase |
| 60 | rna-XM_016614904.1 | LOC107792668 | mRNA | 6041 | 14659 | 1.031 | phenylalanine ammonia-lyase |
| 1 | rna-XM_016621605.1 | LOC107798595 | mRNA | 1278 | 666 | -1.188 | uncharacterized LOC107798595 |
| 2 | rna-XM_016640248.1 | LOC107814785 | mRNA | 1988 | 885 | -1.415 | aquaporin TIP2-1-like |
| 3 | rna-XM_016599462.1 | LOC107779121 | mRNA | 590 | 259 | -1.436 | expansin-A4-like |
| 4 | rna-XM_016626020.1 | LOC107802501 | mRNA | 4358 | 1864 | -1.473 | aquaporin TIP2-1-like |
| 5 | rna-XM_016617367.1 | LOC107794830 | mRNA | 328 | 97 | -2.006 | peroxidase 21-like |
| 6 | rna-XM_016638433.1 | LOC107813195 | mRNA | 286 | 67 | -2.342 | uncharacterized LOC107813195, transcript variant X2 |
| 7 | rna-XM_016638425.1 | LOC107813195 | mRNA | 286 | 67 | -2.342 | uncharacterized LOC107813195, transcript variant X1 |
| 8 | rna-XR_001647814.1 | LOC107785172 | misc_RNA | 359 | 71 | -2.586 | **auxin response factor 3-like, transcript variant X1** |
| 9 | rna-XR_001647815.1 | LOC107785172 | misc_RNA | 359 | 71 | -2.586 | **auxin response factor 3-like, transcript variant X3** |
| 10 | rna-XM_016606410.1 | LOC107785172 | mRNA | 359 | 71 | -2.586 | **auxin response factor 3-like, transcript variant X2** |
| 11 | rna-XM_016627510.1 | LOC107803745 | mRNA | 633 | 125 | -2.588 | **auxin response factor 3-like, transcript variant X2** |
| 12 | rna-XR_001652067.1 | LOC107803745 | misc_RNA | 633 | 124 | -2.600 | **auxin response factor 3-like, transcript variant X1** |
| 13 | rna-XR_001652068.1 | LOC107803745 | misc_RNA | 633 | 124 | -2.600 | **auxin response factor 3-like, transcript variant X3** |
| 14 | rna-XR_001657810.1 | LOC107828916 | ncRNA | 132 | 15 | -3.385 | uncharacterized LOC107828916 |
| 15 | rna-XM_016653169.1 | LOC107826218 | mRNA | 813 | 51 | -4.243 | **auxin response factor 4-like** |
| 16 | rna-XM_016606813.1 | LOC107785490 | mRNA | 1003 | 55 | -4.437 | **auxin response factor 4-like** |

Table S23. The differentially expressed genes in flowers of *rdr6* plants vs TAS3i plants

|  |  |  |  | expression amounts in flowers of | |  |  |
| --- | --- | --- | --- | --- | --- | --- | --- |
|  | Gene_ID | Gene | Gbkey | rdr6 plants | TAS3i plants | m.value [log2(expression ratio)] | Product |
| 1 | rna-XM_016641299.1 | LOC107815675 | mRNA | 0 | 207 | 7.392 | zinc finger BED domain-containing protein RICESLEEPER 3-like |
| 2 | rna-XM_016641004.1 | LOC107815427 | mRNA | 1 | 169 | 7.099 | defensin-like protein 19 |
| 3 | rna-XM_016638433.1 | LOC107813195 | mRNA | 3 | 109 | 4.882 | uncharacterized LOC107813195, transcript variant X2 |
| 4 | rna-XM_016638425.1 | LOC107813195 | mRNA | 3 | 109 | 4.882 | uncharacterized LOC107813195, transcript variant X1 |
| 5 | rna-XM_016635038.1 | LOC107810284 | mRNA | 3 | 71 | 4.263 | peroxidase 72-like |
| 6 | rna-XM_016624910.1 | LOC107801566 | mRNA | 7 | 140 | 4.020 | peroxidase 72-like |
| 7 | rna-XM_016623821.1 | LOC107800618 | mRNA | 22 | 238 | 3.134 | cytochrome P450 78A6-like |
| 8 | rna-XM_016624551.1 | LOC107801261 | mRNA | 17 | 171 | 3.029 | WAG22 antigen-like |
| 9 | rna-XM_016642073.1 | LOC107816366 | mRNA | 384 | 2137 | 2.175 | cysteine protease inhibitor 8-like |
| 10 | rna-XM_016616425.1 | LOC107793976 | mRNA | 43 | 231 | 2.124 | protochlorophyllide reductase-like |
| 11 | rna-XM_016657559.1 | LOC107830074 | mRNA | 59 | 308 | 2.083 | uncharacterized LOC107830074, transcript variant X2 |
| 12 | rna-XM_016600162.1 | LOC107779687 | mRNA | 54 | 279 | 2.068 | transcription factor EMB1444-like, transcript variant X1 |
| 13 | rna-XM_016600170.1 | LOC107779687 | mRNA | 54 | 279 | 2.068 | transcription factor EMB1444-like, transcript variant X2 |
| 14 | rna-XM_016657558.1 | LOC107830074 | mRNA | 66 | 341 | 2.068 | uncharacterized LOC107830074, transcript variant X1 |
| 15 | rna-XM_016631569.1 | LOC107807226 | mRNA | 198 | 982 | 2.009 | aquaporin TIP1-3-like |
| 16 | rna-XM_016646225.1 | LOC107820018 | mRNA | 93 | 435 | 1.924 | aquaporin TIP1-3-like |
| 17 | rna-XM_016646587.1 | LOC107820326 | mRNA | 164 | 758 | 1.907 | protochlorophyllide reductase-like |
| 18 | rna-XM_016593918.1 | LOC107774398 | mRNA | 280 | 1291 | 1.904 | ribulose bisphosphate carboxylase small chain S41, chloroplastic-like |
| 19 | rna-XM_016631601.1 | LOC107807254 | mRNA | 109 | 495 | 1.882 | chlorophyll a-b binding protein 7, chloroplastic |
| 20 | gene-LOC107796867 | LOC107796867 | exon | 78 | 338 | 1.814 |  |
| 21 | rna-XM_016590181.1 | LOC107770844 | mRNA | 3109 | 13055 | 1.769 | chlorophyll a-b binding protein 50, chloroplastic-like |
| 22 | rna-XM_016623020.1 | LOC107799889 | mRNA | 125 | 523 | 1.763 | cysteine protease inhibitor 8-like |
| 23 | rna-XM_016612928.1 | LOC107790956 | mRNA | 172 | 703 | 1.730 | chlorophyll a-b binding protein 36, chloroplastic-like |
| 24 | rna-XM_016603253.1 | LOC107782372 | mRNA | 125 | 494 | 1.681 | pectinesterase-like |
| 25 | rna-XM_016584879.1 | LOC107766137 | mRNA | 126 | 485 | 1.643 | ABC transporter G family member 22-like, transcript variant X1 |
| 26 | rna-XM_016584881.1 | LOC107766137 | mRNA | 126 | 485 | 1.643 | ABC transporter G family member 22-like, transcript variant X3 |
| 27 | rna-XM_016604537.1 | LOC107783553 | mRNA | 126 | 485 | 1.643 | cyanidin-3-O-glucoside 2-O-glucuronosyltransferase-like |
| 28 | rna-XM_016584880.1 | LOC107766137 | mRNA | 126 | 484 | 1.640 | ABC transporter G family member 22-like, transcript variant X2 |
| 29 | rna-XM_016613856.1 | LOC107791735 | mRNA | 555 | 2127 | 1.637 | defensin-like protein |
| 30 | rna-XM_016659547.1 | LOC107831753 | mRNA | 1211 | 4515 | 1.597 | defensin-like protein |
| 31 | rna-XM_016623090.1 | LOC107799946 | mRNA | 173 | 625 | 1.552 | protein phosphatase 1 regulatory subunit pprA-like, transcript variant X2 |
| 32 | rna-XM_016623089.1 | LOC107799946 | mRNA | 173 | 625 | 1.552 | protein phosphatase 1 regulatory subunit pprA-like, transcript variant X1 |
| 33 | rna-XM_016658144.1 | LOC107830543 | mRNA | 701 | 2513 | 1.540 | chlorophyll a-b binding protein 4, chloroplastic-like |
| 34 | gene-LOC107768296 | LOC107768296 | exon | 678 | 2398 | 1.521 |  |
| 35 | rna-XM_016659546.1 | LOC107831752 | mRNA | 390 | 1349 | 1.489 | defensin-like protein |
| 36 | rna-XM_016614043.1 | LOC107791896 | mRNA | 548 | 1885 | 1.481 | chlorophyll a-b binding protein 7, chloroplastic |
| 37 | rna-XM_016600904.1 | LOC107780379 | mRNA | 2639 | 8992 | 1.467 | chlorophyll a-b binding protein 50, chloroplastic |
| 38 | rna-XM_016586428.1 | LOC107767419 | mRNA | 342 | 1152 | 1.451 | photosystem I subunit O-like |
| 39 | rna-XM_016643125.1 | LOC107817323 | mRNA | 1351 | 4547 | 1.449 | chlorophyll a-b binding protein 8, chloroplastic-like |
| 40 | rna-XM_016657680.1 | LOC107830195 | mRNA | 3452 | 11580 | 1.445 | chlorophyll a-b binding protein 13, chloroplastic-like |
| 41 | rna-XM_016583686.1 | LOC107765079 | mRNA | 1106 | 3704 | 1.442 | chlorophyll a-b binding protein 13, chloroplastic-like |
| 42 | rna-XM_016634545.1 | LOC107809852 | mRNA | 5978 | 19942 | 1.437 | chlorophyll a-b binding protein 36, chloroplastic-like |
| 43 | rna-XM_016631014.1 | LOC107806780 | mRNA | 412 | 1351 | 1.412 | chlorophyll a-b binding protein 40, chloroplastic-like |
| 44 | rna-XM_016632344.1 | LOC107807891 | mRNA | 4944 | 15406 | 1.338 | chlorophyll a-b binding protein 21, chloroplastic-like |
| 45 | rna-XM_016606190.1 | LOC107784985 | mRNA | 2767 | 8317 | 1.286 | photosystem I subunit O-like |
| 46 | rna-XM_016605780.1 | LOC107784622 | mRNA | 1963 | 5866 | 1.278 | chlorophyll a-b binding protein 8, chloroplastic-like |
| 1 | rna-XM_016647624.1 | LOC107821202 | mRNA | 2951 | 1459 | -1.318 | galactinol synthase 2 |
| 2 | rna-XM_016625309.1 | LOC107801897 | mRNA | 715 | 300 | -1.554 | histone H3.3 |
| 3 | rna-XM_016616708.1 | LOC107794227 | mRNA | 380 | 140 | -1.742 | probable xyloglucan endotransglucosylase/hydrolase protein 23, transcript variant X2 |
| 4 | rna-XM_016616706.1 | LOC107794227 | mRNA | 380 | 140 | -1.742 | probable xyloglucan endotransglucosylase/hydrolase protein 23, transcript variant X1 |
| 5 | rna-XM_016624615.1 | LOC107801304 | mRNA | 540 | 197 | -1.756 | bidirectional sugar transporter SWEET12-like |
| 6 | rna-XM_016591710.1 | LOC107772218 | mRNA | 236 | 74 | -1.975 | bidirectional sugar transporter SWEET10-like |
| 7 | rna-XM_016660375.1 | LOC107832511 | mRNA | 1368 | 419 | -2.009 | probable aspartic protease At2g35615 |
| 8 | rna-XM_016601930.1 | LOC107781251 | mRNA | 357 | 106 | -2.053 | non-specific lipid-transfer protein 3-like |
| 9 | rna-XM_016627510.1 | LOC107803745 | mRNA | 1684 | 471 | -2.140 | **auxin response factor 3-like, transcript variant X2** |
| 10 | rna-XR_001652067.1 | LOC107803745 | misc_RNA | 1683 | 470 | -2.142 | **auxin response factor 3-like, transcript variant X1** |
| 11 | rna-XR_001652068.1 | LOC107803745 | misc_RNA | 1683 | 470 | -2.142 | **auxin response factor 3-like, transcript variant X3** |
| 12 | rna-XM_016638376.1 | LOC107813154 | mRNA | 256 | 71 | -2.152 | gibberellin-regulated protein 1-like, transcript variant X2 |
| 13 | rna-XM_016638375.1 | LOC107813154 | mRNA | 256 | 71 | -2.152 | gibberellin-regulated protein 1-like, transcript variant X1 |
| 14 | rna-XM_016600359.1 | LOC107779861 | mRNA | 1701 | 468 | -2.163 | non-specific lipid-transfer protein 3-like |
| 15 | rna-XR_001647814.1 | LOC107785172 | misc_RNA | 907 | 234 | -2.256 | **auxin response factor 3-like, transcript variant X1** |
| 16 | rna-XR_001647815.1 | LOC107785172 | misc_RNA | 907 | 234 | -2.256 | **auxin response factor 3-like, transcript variant X3** |
| 17 | rna-XM_016606410.1 | LOC107785172 | mRNA | 907 | 234 | -2.256 | **auxin response factor 3-like, transcript variant X2** |
| 18 | rna-XM_016630594.1 | LOC107806436 | mRNA | 299 | 77 | -2.259 | indole-3-acetic acid-amido synthetase GH3.6-like |
| 19 | rna-XM_016591148.1 | LOC107771705 | mRNA | 848 | 218 | -2.261 | snakin-2-like |
| 20 | rna-XM_016591891.1 | LOC107772392 | mRNA | 133 | 26 | -2.656 | probable glutathione S-transferase, transcript variant X1 |
| 21 | rna-XM_016606813.1 | LOC107785490 | mRNA | 1091 | 160 | -3.071 | **auxin response factor 4-like** |
| 22 | rna-XM_016653169.1 | LOC107826218 | mRNA | 913 | 115 | -3.290 | **auxin response factor 4-like** |
| 23 | rna-XM_016650053.1 | LOC107823411 | mRNA | 97 | 11 | -3.442 | glucan endo-1,3-beta-glucosidase, basic vacuolar |
| 24 | rna-XM_016616341.1 | LOC107793895 | mRNA | 63 | 4 | -4.279 | suberization-associated anionic peroxidase-like |
| 25 | rna-XM_016655118.1 | LOC107827891 | mRNA | 380 | 22 | -4.412 | proteinase inhibitor I-B-like |
| 26 | rna-XM_016609426.1 | LOC107787819 | mRNA | 128 | 7 | -4.494 | osmotin |

Table S24. The differentially expressed genes in buds of *rdr6* plants vs TAS3i plants

|  |  |  |  | expression amounts in buds of | |  |  |
| --- | --- | --- | --- | --- | --- | --- | --- |
|  | Gene_ID | Gene | Gbkey | rdr6 plants | TAS3i plants | m.value [log2(expression ratio)] | Product |
| 1 | rna-XM_016641299.1 | LOC107815675 | mRNA | 0 | 129 | 7.064 | zinc finger BED domain-containing protein RICESLEEPER 3-like |
| 2 | rna-XM_016609596.1 | LOC107787967 | mRNA | 146 | 936 | 2.733 | patatin-like protein 2 |
| 3 | rna-XM_016628544.1 | LOC107804626 | mRNA | 33 | 143 | 2.168 | uncharacterized LOC107804626, transcript variant X1 |
| 4 | rna-XM_016657558.1 | LOC107830074 | mRNA | 146 | 627 | 2.155 | uncharacterized LOC107830074, transcript variant X1 |
| 5 | rna-XM_016657559.1 | LOC107830074 | mRNA | 136 | 563 | 2.102 | uncharacterized LOC107830074, transcript variant X2 |
| 6 | rna-XM_016586702.1 | LOC107767635 | mRNA | 123 | 487 | 2.038 | subtilisin-like protease SBT2.5 |
| 7 | rna-XM_016605916.1 | LOC107784740 | mRNA | 234 | 922 | 2.031 | stamen-specific protein FIL1-like |
| 8 | rna-XM_016639596.1 | LOC107814221 | mRNA | 269 | 1005 | 1.954 | protein 108-like |
| 9 | rna-XM_016654280.1 | LOC107827188 | mRNA | 67 | 237 | 1.876 | L-ascorbate oxidase homolog |
| 10 | rna-XM_016596613.1 | LOC107776702 | mRNA | 60 | 209 | 1.853 | putative non-specific lipid-transfer protein 14 |
| 11 | rna-XM_016623090.1 | LOC107799946 | mRNA | 134 | 455 | 1.817 | protein phosphatase 1 regulatory subunit pprA-like, transcript variant X2 |
| 12 | rna-XM_016623089.1 | LOC107799946 | mRNA | 134 | 455 | 1.817 | protein phosphatase 1 regulatory subunit pprA-like, transcript variant X1 |
| 13 | rna-XM_016611858.1 | LOC107789975 | mRNA | 194 | 619 | 1.727 | endochitinase EP3-like |
| 14 | rna-XM_016631569.1 | LOC107807226 | mRNA | 204 | 566 | 1.525 | aquaporin TIP1-3-like |
| 15 | rna-XM_016612037.1 | LOC107790131 | mRNA | 199 | 510 | 1.411 | peroxidase 16-like |
| 16 | rna-XM_016617904.1 | LOC107795297 | mRNA | 556 | 1405 | 1.390 | GDSL esterase/lipase At4g26790-like |
| 17 | rna-XM_016629048.1 | LOC107805069 | mRNA | 803 | 1860 | 1.265 | fatty-acid-binding protein 1-like |
| 18 | rna-XM_016598812.1 | LOC107778533 | mRNA | 4017 | 8488 | 1.132 | photosystem II 22 kDa protein, chloroplastic |
| 19 | rna-XM_016658144.1 | LOC107830543 | mRNA | 2941 | 6191 | 1.127 | chlorophyll a-b binding protein 4, chloroplastic-like |
| 1 | rna-XM_016604448.1 | LOC107783475 | mRNA | 1346 | 553 | -1.230 | ABC transporter G family member 26-like |
| 2 | rna-XM_016642899.1 | LOC107817126 | mRNA | 4398 | 1691 | -1.326 | anther-specific protein LAT52-like |
| 3 | rna-XM_016605728.1 | LOC107784583 | mRNA | 692 | 265 | -1.332 | 1-aminocyclopropane-1-carboxylate oxidase-like |
| 4 | rna-XM_016600018.1 | LOC107779568 | mRNA | 734 | 272 | -1.379 | caffeoylshikimate esterase-like |
| 5 | rna-XM_016594643.1 | LOC107774983 | mRNA | 2265 | 769 | -1.506 | tetraketide alpha-pyrone reductase 1-like |
| 6 | gene-LOC107799858 | LOC107799858 | exon | 2845 | 914 | -1.585 |  |
| 7 | rna-XM_016601951.1 | LOC107781266 | mRNA | 883 | 259 | -1.717 | type III polyketide synthase A-like |
| 8 | rna-XM_016620041.1 | LOC107797177 | mRNA | 848 | 246 | -1.732 | sugar transport protein 10-like |
| 9 | rna-XM_016597357.1 | LOC107777350 | mRNA | 1076 | 293 | -1.824 | protein HOTHEAD-like |
| 10 | rna-XM_016585778.1 | LOC107766889 | mRNA | 797 | 202 | -1.927 | L-ascorbate oxidase homolog |
| 11 | rna-XM_016640326.1 | LOC107814848 | mRNA | 5715 | 1443 | -1.933 | L-ascorbate oxidase homolog |
| 12 | rna-XM_016622136.1 | LOC107799065 | mRNA | 242 | 59 | -1.983 | pectin acetylesterase 7-like |
| 13 | rna-XM_016581596.1 | LOC107763147 | mRNA | 1246 | 303 | -1.987 | sugar transport protein 8-like |
| 14 | rna-XM_016658653.1 | LOC107830954 | mRNA | 211 | 51 | -1.996 | type III polyketide synthase A-like |
| 15 | rna-XM_016608743.1 | LOC107787207 | mRNA | 3188 | 764 | -2.008 | sugar transport protein 8-like |
| 16 | rna-XR_001652067.1 | LOC107803745 | misc_RNA | 768 | 183 | -2.016 | **auxin response factor 3-like, transcript variant X1** |
| 17 | rna-XR_001652068.1 | LOC107803745 | misc_RNA | 768 | 183 | -2.016 | **auxin response factor 3-like, transcript variant X3** |
| 18 | rna-XM_016627510.1 | LOC107803745 | mRNA | 768 | 183 | -2.016 | **auxin response factor 3-like, transcript variant X2** |
| 19 | rna-XR_001647814.1 | LOC107785172 | misc_RNA | 431 | 102 | -2.026 | **auxin response factor 3-like, transcript variant X1** |
| 20 | rna-XR_001647815.1 | LOC107785172 | misc_RNA | 431 | 102 | -2.026 | **auxin response factor 3-like, transcript variant X3** |
| 21 | rna-XM_016606410.1 | LOC107785172 | mRNA | 431 | 102 | -2.026 | **auxin response factor 3-like, transcript variant X2** |
| 22 | rna-XM_016635776.1 | LOC107810940 | mRNA | 238 | 55 | -2.061 | probable aquaporin NIP-type, transcript variant X2 |
| 23 | rna-XM_016635775.1 | LOC107810940 | mRNA | 238 | 55 | -2.061 | probable aquaporin NIP-type, transcript variant X1 |
| 24 | rna-XM_016635928.1 | LOC107811073 | mRNA | 655 | 151 | -2.064 | protein HOTHEAD-like |
| 25 | rna-XM_016607761.1 | LOC107786280 | mRNA | 171 | 39 | -2.080 | senescence-specific cysteine protease SAG12-like |
| 26 | rna-XM_016653577.1 | LOC107826589 | mRNA | 203 | 46 | -2.089 | two-component response regulator-like APRR1 |
| 27 | rna-XM_016607296.1 | LOC107785898 | mRNA | 345 | 78 | -2.092 | ADP,ATP carrier protein 1, mitochondrial-like, transcript variant X2 |
| 28 | rna-XM_016607294.1 | LOC107785898 | mRNA | 345 | 78 | -2.092 | ADP,ATP carrier protein 1, mitochondrial-like, transcript variant X1 |
| 29 | rna-XM_016613483.1 | LOC107791425 | mRNA | 227 | 48 | -2.189 | cytochrome P450 704B1-like |
| 30 | rna-XM_016599645.1 | LOC107779248 | mRNA | 253 | 53 | -2.202 | senescence-specific cysteine protease SAG12-like |
| 31 | rna-XM_016591148.1 | LOC107771705 | mRNA | 295 | 60 | -2.245 | snakin-2-like |
| 32 | rna-XM_016634320.1 | LOC107809664 | mRNA | 1362 | 264 | -2.314 | cytochrome P450 704B1-like |
| 33 | rna-XM_016607676.1 | LOC107786229 | mRNA | 157 | 27 | -2.487 | cystathionine beta-lyase, chloroplastic-like |
| 34 | rna-XM_016636216.1 | LOC107811316 | mRNA | 246 | 42 | -2.497 | desiccation-related protein PCC13-62-like |
| 35 | rna-XM_016580156.1 | LOC107761866 | mRNA | 182 | 30 | -2.548 | WSC domain-containing protein ARB_07867-like |
| 36 | rna-XM_016626713.1 | LOC107803097 | mRNA | 110 | 18 | -2.558 | dihydroflavonol-4-reductase-like |
| 37 | rna-XM_016596165.1 | LOC107776287 | mRNA | 342 | 55 | -2.584 | glyceraldehyde-3-phosphate dehydrogenase, cytosolic |
| 38 | rna-XM_016620104.1 | LOC107797232 | mRNA | 292 | 46 | -2.613 | dihydroflavonol-4-reductase |
| 39 | gene-LOC107777646 | LOC107777646 | exon | 167 | 25 | -2.687 |  |
| 40 | rna-XM_016621785.1 | LOC107798752 | mRNA | 615 | 87 | -2.769 | glucan endo-1,3-beta-glucosidase 8-like |
| 41 | rna-XM_016587497.1 | LOC107768376 | mRNA | 1198 | 169 | -2.773 | pectinesterase-like |
| 42 | rna-XM_016615588.1 | LOC107793260 | mRNA | 345 | 48 | -2.793 | pathogenesis-related protein 5-like |
| 43 | rna-XM_016582845.1 | LOC107764290 | mRNA | 572 | 71 | -2.957 | pectinesterase-like |
| 44 | rna-XM_016647544.1 | LOC107821133 | mRNA | 262 | 31 | -3.026 | polygalacturonase-like |
| 45 | rna-XM_016640257.1 | LOC107814800 | mRNA | 373 | 44 | -3.031 | non-specific lipid-transfer protein 2 |
| 46 | rna-XM_016604982.1 | LOC107783954 | mRNA | 188 | 22 | -3.042 | uncharacterized LOC107783954 |
| 47 | rna-XM_016602699.1 | LOC107781884 | mRNA | 173 | 20 | -3.060 | pectinesterase-like |
| 48 | rna-XM_016605970.1 | LOC107784787 | mRNA | 82 | 9 | -3.135 | probable methyltransferase PMT27 |
| 49 | rna-XM_016617087.1 | LOC107794590 | mRNA | 3202 | 343 | -3.170 | fatty acyl-CoA reductase 2-like |
| 50 | rna-XM_016610673.1 | LOC107788932 | mRNA | 1104 | 116 | -3.198 | cytochrome P450 703A2-like |
| 51 | rna-XM_016604204.1 | LOC107783237 | mRNA | 255 | 26 | -3.241 | glucan endo-1,3-beta-glucosidase 8-like |
| 52 | rna-XM_016639856.1 | LOC107814441 | mRNA | 357 | 36 | -3.257 | cytochrome P450 703A2-like |
| 53 | rna-XM_016582050.1 | LOC107763563 | mRNA | 659 | 64 | -3.311 | beta-galactosidase 13-like |
| 54 | rna-XM_016584460.1 | LOC107765773 | mRNA | 1604 | 141 | -3.455 | fatty acyl-CoA reductase 2-like |
| 55 | rna-XM_016593286.1 | LOC107773859 | mRNA | 487 | 42 | -3.483 | beta-fructofuranosidase, insoluble isoenzyme 1-like |
| 56 | rna-XM_016606813.1 | LOC107785490 | mRNA | 2598 | 218 | -3.522 | **auxin response factor 4-like** |
| 57 | rna-XM_016643361.1 | LOC107817512 | mRNA | 97 | 8 | -3.547 | GPI-anchored protein LORELEI-like |
| 58 | rna-XM_016595805.1 | LOC107776000 | mRNA | 99 | 8 | -3.576 | phosphoenolpyruvate carboxykinase [ATP]-like |
| 59 | rna-XM_016660016.1 | LOC107832171 | mRNA | 311 | 24 | -3.643 | late embryogenesis abundant protein 1-like |
| 60 | rna-XM_016580091.1 | LOC107761808 | mRNA | 1211 | 93 | -3.650 | uncharacterized LOC107761808 |
| 61 | rna-XM_016626267.1 | LOC107802698 | mRNA | 66 | 5 | -3.670 | pathogenesis-related protein 5-like |
| 62 | rna-XM_016628700.1 | LOC107804769 | mRNA | 522 | 38 | -3.727 | ATPase 9, plasma membrane-type |
| 63 | rna-XM_016653169.1 | LOC107826218 | mRNA | 2107 | 153 | -3.731 | **auxin response factor 4-like** |
| 64 | rna-XM_016604121.1 | LOC107783148 | mRNA | 726 | 50 | -3.807 | ATPase 8, plasma membrane-type |
| 65 | rna-XM_016607668.1 | LOC107786220 | mRNA | 149 | 9 | -3.996 | major pollen allergen Ole e 6-like |
| 66 | rna-XM_016640030.1 | LOC107814593 | mRNA | 158 | 9 | -4.081 | cytochrome b5-like |
| 67 | rna-XM_016644487.1 | LOC107818462 | mRNA | 77 | 4 | -4.214 | external alternative NAD(P)H-ubiquinone oxidoreductase B3, mitochondrial-like |
| 68 | rna-XM_016593809.1 | LOC107774312 | mRNA | 58 | 3 | -4.220 | vicilin-like seed storage protein At2g28490 |
| 69 | rna-XM_016580587.1 | LOC107762241 | mRNA | 430 | 21 | -4.303 | anther-specific protein LAT52-like |
| 70 | rna-XM_016612677.1 | LOC107790719 | mRNA | 64 | 3 | -4.362 | vicilin-like seed storage protein At2g28490 |
| 71 | rna-XM_016588294.1 | LOC107769110 | mRNA | 425 | 19 | -4.430 | anther-specific protein LAT52-like |
| 72 | rna-XM_016647509.1 | LOC107821107 | mRNA | 206 | 8 | -4.634 | late embryogenesis abundant protein 1-like |
| 73 | rna-XM_016612117.1 | LOC107790214 | mRNA | 289 | 8 | -5.122 | stress-induced protein KIN2-like |
| 74 | rna-XM_016639178.1 | LOC107813865 | mRNA | 77 | 2 | -5.214 | classical arabinogalactan protein 6-like |
| 75 | rna-XM_016625802.1 | LOC107802330 | mRNA | 117 | 3 | -5.232 | histone H2B-like |
| 76 | rna-XM_016643049.1 | LOC107817249 | mRNA | 125 | 3 | -5.328 | exopolygalacturonase-like |
| 77 | rna-XM_016592973.1 | LOC107773574 | mRNA | 734 | 14 | -5.659 | L-ascorbate oxidase homolog |
| 78 | gene-LOC107832625 | LOC107832625 | exon | 84 | 1 | -6.339 |  |
| 79 | rna-XM_016633195.1 | LOC107808657 | mRNA | 95 | 1 | -6.517 | aquaporin-like, transcript variant X1 |
| 80 | rna-XM_016633197.1 | LOC107808657 | mRNA | 95 | 1 | -6.517 | aquaporin-like, transcript variant X3 |
| 81 | rna-XM_016633196.1 | LOC107808657 | mRNA | 95 | 1 | -6.517 | aquaporin-like, transcript variant X2 |
| 82 | rna-XM_016660639.1 | LOC107832765 | mRNA | 108 | 1 | -6.702 | pectate lyase-like |
| 83 | rna-XM_016578652.1 | LOC107760582 | mRNA | 223 | 2 | -6.748 | WAT1-related protein At2g39510-like |
| 84 | rna-XM_016659821.1 | LOC107832019 | mRNA | 261 | 2 | -6.975 | pectinesterase 4-like, transcript variant X1 |
| 85 | rna-XM_016659822.1 | LOC107832019 | mRNA | 261 | 2 | -6.975 | pectinesterase 4-like, transcript variant X2 |
| 86 | rna-XM_016633778.1 | LOC107809188 | mRNA | 391 | 2 | -7.558 | aquaporin-1-like |
| 87 | rna-XM_016641191.1 | LOC107815555 | mRNA | 48 | 0 | -5.532 | stress-induced protein KIN2-like |
| 88 | rna-XM_016576817.1 | LOC107758965 | mRNA | 40 | 0 | -5.269 | pyruvate decarboxylase 2-like |
| 89 | rna-XM_016606551.1 | LOC107785281 | mRNA | 68 | 0 | -6.035 | stress-induced protein KIN1-like |
| 90 | rna-XM_016641985.1 | LOC107816279 | mRNA | 55 | 0 | -5.728 | uncharacterized LOC107816279 |
| 91 | rna-XM_016578957.1 | LOC107760849 | mRNA | 71 | 0 | -6.097 | WAT1-related protein At2g39510-like |
| 92 | rna-XM_016579585.1 | LOC107761357 | mRNA | 52 | 0 | -5.647 | uncharacterized LOC107761357 |
| 93 | rna-XM_016585282.1 | LOC107766486 | mRNA | 55 | 0 | -5.728 | V-type proton ATPase subunit E-like |
| 94 | rna-XR_001645813.1 | LOC107775692 | ncRNA | 63 | 0 | -5.924 | uncharacterized LOC107775692 |
| 95 | rna-XM_016630823.1 | LOC107806631 | mRNA | 57 | 0 | -5.780 | pectate lyase-like, transcript variant X1 |
| 96 | rna-XM_016630824.1 | LOC107806631 | mRNA | 57 | 0 | -5.780 | pectate lyase-like, transcript variant X2 |

Table S25. The GO analysis of up-regulated genes in leaves (*rdr6* vs TAS3i plants)

| No | GO_term | GOid | All | DEG | P-value | Ontologies |
| --- | --- | --- | --- | --- | --- | --- |
| 1 | carbon utilization | GO:0015976 | 29 | 10 | 8.30E-20 | biological_process |
| 2 | carbonate dehydratase activity | GO:0004089 | 56 | 10 | 1.35E-17 | molecular_function |
| 3 | lyase activity | GO:0016829 | 671 | 16 | 2.83E-16 | molecular_function |
| 4 | negative regulation of endopeptidase activity | GO:0010951 | 174 | 7 | 1.01E-07 | biological_process |
| 5 | serine-type endopeptidase inhibitor activity | GO:0004867 | 44 | 5 | 3.03E-07 | molecular_function |
| 6 | glucose-1-phosphate adenylyltransferase activity | GO:0008878 | 18 | 4 | 1.39E-06 | molecular_function |
| 7 | glycogen biosynthetic process | GO:0005978 | 41 | 4 | 2.40E-05 | biological_process |
| 8 | starch biosynthetic process | GO:0019252 | 47 | 4 | 3.52E-05 | biological_process |
| 9 | killing of cells of other organism | GO:0031640 | 46 | 3 | 0.0032633 | biological_process |
| 10 | adenylate cyclase activity | GO:0004016 | 5 | 2 | 0.0043785 | molecular_function |
| 11 | cAMP biosynthetic process | GO:0006171 | 5 | 2 | 0.0043785 | biological_process |
| 12 | cinnamic acid biosynthetic process | GO:0009800 | 8 | 2 | 0.0073615 | biological_process |
| 13 | ammonia-lyase activity | GO:0016841 | 8 | 2 | 0.0073615 | molecular_function |
| 14 | phenylalanine ammonia-lyase activity | GO:0045548 | 8 | 2 | 0.0073615 | molecular_function |
| 15 | L-phenylalanine catabolic process | GO:0006559 | 11 | 2 | 0.0118924 | biological_process |
| 16 | phenylpropanoid metabolic process | GO:0009698 | 17 | 2 | 0.0243729 | biological_process |
| 17 | response to wounding | GO:0009611 | 134 | 3 | 0.0375214 | biological_process |
| 18 | biosynthetic process | GO:0009058 | 415 | 4 | 0.0629555 | biological_process |
| 19 | endopeptidase inhibitor activity | GO:0004866 | 31 | 2 | 0.0629555 | molecular_function |
| 20 | defense response to fungus | GO:0050832 | 175 | 3 | 0.068803 | biological_process |
| 21 | plastid | GO:0009536 | 827 | 5 | 0.0787181 | cellular_component |
| 22 | nucleotidyltransferase activity | GO:0016779 | 476 | 4 | 0.0878654 | molecular_function |
| 23 | intramolecular transferase activity | GO:0016866 | 43 | 2 | 0.096961 | molecular_function |
| 24 | vacuole | GO:0005773 | 384 | 3 | 0.5327812 | cellular_component |
| 25 | myrcene synthase activity | GO:0050551 | 4 | 1 | 0.6187819 | molecular_function |
| 26 | (4S)-limonene synthase activity | GO:0050552 | 4 | 1 | 0.6187819 | molecular_function |
| 27 | terpene synthase activity | GO:0010333 | 142 | 2 | 0.819843 | molecular_function |

Table S26. The GO analysis of down-regulated genes in leaves (*rdr6* vs TAS3i plants)

| No | GO_term | GOid | All | DEG | P-value | Ontologies |
| --- | --- | --- | --- | --- | --- | --- |
| 1 | vegetative phase change | GO:0010050 | 8 | 6 | 6.82E-15 | biological_process |
| 2 | abaxial cell fate specification | GO:0010158 | 10 | 6 | 9.08E-15 | biological_process |
| 3 | response to hormone | GO:0009725 | 148 | 8 | 3.88E-13 | biological_process |
| 4 | auxin-activated signaling pathway | GO:0009734 | 326 | 8 | 1.36E-10 | biological_process |
| 5 | floral meristem determinacy | GO:0010582 | 22 | 4 | 8.85E-08 | biological_process |
| 6 | transcription, DNA-templated | GO:0006351 | 2881 | 8 | 0.0016732 | biological_process |
| 7 | DNA binding | GO:0003677 | 6192 | 9 | 0.0474277 | molecular_function |
| 8 | regulation of transcription, DNA-templated | GO:0006355 | 4728 | 8 | 0.0474277 | biological_process |
| 9 | plant-type cell wall organization | GO:0009664 | 138 | 2 | 0.3933254 | biological_process |
| 10 | nucleus | GO:0005634 | 7597 | 8 | 0.7662766 | cellular_component |

Table S27. GO analysis of up-regulated genes in flowers (*rdr6* vs TAS3i plants)

| No | GO_term | GOid | All | DEG | P-value | Ontologies |
| --- | --- | --- | --- | --- | --- | --- |
| 1 | photosynthesis, light harvesting | GO:0009765 | 90 | 13 | 3.01E-20 | biological_process |
| 2 | chlorophyll binding | GO:0016168 | 117 | 13 | 3.59E-19 | molecular_function |
| 3 | protein-chromophore linkage | GO:0018298 | 121 | 13 | 3.61E-19 | biological_process |
| 4 | photosystem I | GO:0009522 | 146 | 13 | 2.70E-18 | cellular_component |
| 5 | photosystem II | GO:0009523 | 175 | 13 | 2.01E-17 | cellular_component |
| 6 | photosynthesis | GO:0015979 | 284 | 14 | 1.37E-16 | biological_process |
| 7 | thylakoid | GO:0009579 | 319 | 13 | 2.47E-14 | cellular_component |
| 8 | chloroplast thylakoid membrane | GO:0009535 | 464 | 13 | 2.28E-12 | cellular_component |
| 9 | plastid | GO:0009536 | 827 | 14 | 1.41E-10 | cellular_component |
| 10 | chloroplast | GO:0009507 | 2576 | 16 | 5.01E-06 | cellular_component |
| 11 | killing of cells of other organism | GO:0031640 | 46 | 4 | 8.51E-05 | biological_process |
| 12 | adenylate cyclase activity | GO:0004016 | 5 | 2 | 0.007049 | molecular_function |
| 13 | cAMP biosynthetic process | GO:0006171 | 5 | 2 | 0.007049 | biological_process |
| 14 | defense response to fungus | GO:0050832 | 175 | 4 | 0.010854 | biological_process |
| 15 | central vacuole | GO:0042807 | 9 | 2 | 0.015959 | cellular_component |
| 16 | protochlorophyllide reductase activity | GO:0016630 | 12 | 2 | 0.024705 | molecular_function |
| 17 | cellular water homeostasis | GO:0009992 | 19 | 2 | 0.047532 | biological_process |
| 18 | water channel activity | GO:0015250 | 19 | 2 | 0.047532 | molecular_function |
| 19 | glycerol channel activity | GO:0015254 | 19 | 2 | 0.047532 | molecular_function |
| 20 | photosynthesis, light harvesting in photosystem I | GO:0009768 | 20 | 2 | 0.047532 | biological_process |
| 21 | pigment binding | GO:0031409 | 20 | 2 | 0.047532 | molecular_function |
| 22 | water transport | GO:0006833 | 21 | 2 | 0.04966 | biological_process |
| 23 | glycerol transport | GO:0015793 | 25 | 2 | 0.065729 | biological_process |
| 24 | endopeptidase inhibitor activity | GO:0004866 | 31 | 2 | 0.094384 | molecular_function |
| 25 | response to light stimulus | GO:0009416 | 45 | 2 | 0.176748 | biological_process |
| 26 | nucleic acid binding | GO:0003676 | 10114 | 1 | 0.176748 | molecular_function |
| 27 | extracellular region | GO:0005576 | 909 | 5 | 0.371923 | cellular_component |
| 28 | cell wall | GO:0005618 | 559 | 4 | 0.390057 | cellular_component |
| 29 | metal ion binding | GO:0046872 | 7430 | 16 | 0.566237 | molecular_function |
| 30 | plant-type vacuole membrane | GO:0009705 | 91 | 2 | 0.566237 | cellular_component |
| 31 | plastoglobule | GO:0010287 | 91 | 2 | 0.566237 | cellular_component |
| 32 | vacuole | GO:0005773 | 384 | 3 | 0.94442 | cellular_component |

Table S28. The GO analysis of down-regulated genes in flowers (*rdr6* vs TAS3i plants)

| No | GO_term | GOid | All | DEG | P-value | Ontologies |
| --- | --- | --- | --- | --- | --- | --- |
| 1 | vegetative phase change | GO:0010050 | 8 | 6 | 1.15E-13 | biological_process |
| 2 | abaxial cell fate specification | GO:0010158 | 10 | 6 | 1.53E-13 | biological_process |
| 3 | response to hormone | GO:0009725 | 148 | 8 | 1.69E-11 | biological_process |
| 4 | auxin-activated signaling pathway | GO:0009734 | 326 | 9 | 9.35E-11 | biological_process |
| 5 | floral meristem determinacy | GO:0010582 | 22 | 4 | 5.71E-07 | biological_process |
| 6 | transcription, DNA-templated | GO:0006351 | 2881 | 8 | 0.048456 | biological_process |
| 7 | extracellular region | GO:0005576 | 909 | 4 | 0.480166 | cellular_component |
| 8 | xyloglucan metabolic process | GO:0010411 | 100 | 2 | 0.480166 | biological_process |
| 9 | cellular glucan metabolic process | GO:0006073 | 101 | 2 | 0.480166 | biological_process |
| 10 | xyloglucan:xyloglucosyl transferase activity | GO:0016762 | 101 | 2 | 0.480166 | molecular_function |
| 11 | cell wall biogenesis | GO:0042546 | 110 | 2 | 0.51514 | biological_process |
| 12 | regulation of transcription, DNA-templated | GO:0006355 | 4728 | 8 | 0.62518 | biological_process |
| 13 | DNA binding | GO:0003677 | 6192 | 9 | 0.727183 | molecular_function |
| 14 | cell wall | GO:0005618 | 559 | 3 | 0.727183 | cellular_component |
| 15 | carbohydrate transport | GO:0008643 | 153 | 2 | 0.727183 | biological_process |
| 16 | lipid transport | GO:0006869 | 186 | 2 | 0.978886 | biological_process |

Table S29. The GO analysis of up-regulated genes in buds (*rdr6* vs TAS3i plants)

| No | GO_term | GOid | All | DEG | P-value | Ontologies |
| --- | --- | --- | --- | --- | --- | --- |
| 1 | adenylate cyclase activity | GO:0004016 | 5 | 2 | 0.00353 | molecular_function |
| 2 | cAMP biosynthetic process | GO:0006171 | 5 | 2 | 0.00353 | biological_process |

Table S30. The GO analysis of down-regulated genes in buds (*rdr6* vs TAS3i plants)

| No | GO_term | GOid | All | DEG | P-value | Ontologies |
| --- | --- | --- | --- | --- | --- | --- |
| 1 | vegetative phase change | GO:0010050 | 8 | 6 | 6.79E-11 | biological_process |
| 2 | abaxial cell fate specification | GO:0010158 | 10 | 6 | 9.03E-11 | biological_process |
| 3 | response to hormone | GO:0009725 | 148 | 8 | 7.84E-08 | biological_process |
| 4 | pectin catabolic process | GO:0045490 | 212 | 8 | 9.02E-07 | biological_process |
| 5 | auxin-activated signaling pathway | GO:0009734 | 326 | 8 | 1.87E-05 | biological_process |
| 6 | floral meristem determinacy | GO:0010582 | 22 | 4 | 3.26E-05 | biological_process |
| 7 | transporter activity | GO:0005215 | 908 | 11 | 3.79E-05 | molecular_function |
| 8 | extracellular space | GO:0005615 | 90 | 5 | 0.000115 | cellular_component |
| 9 | cell wall modification | GO:0042545 | 152 | 5 | 0.00098 | biological_process |
| 10 | aspartyl esterase activity | GO:0045330 | 152 | 5 | 0.00098 | molecular_function |
| 11 | pectinesterase activity | GO:0030599 | 154 | 5 | 0.00098 | molecular_function |
| 12 | enzyme inhibitor activity | GO:0004857 | 195 | 5 | 0.002762 | molecular_function |
| 13 | transport | GO:0006810 | 2879 | 15 | 0.003196 | biological_process |
| 14 | negative regulation of catalytic activity | GO:0043086 | 259 | 5 | 0.009063 | biological_process |
| 15 | pectate lyase activity | GO:0030570 | 58 | 3 | 0.020457 | molecular_function |
| 16 | sporopollenin biosynthetic process | GO:0080110 | 9 | 2 | 0.023632 | biological_process |
| 17 | cell wall | GO:0005618 | 559 | 6 | 0.028405 | cellular_component |
| 18 | transmembrane transporter activity | GO:0022857 | 355 | 5 | 0.029197 | molecular_function |
| 19 | oxidation-reduction process | GO:0055114 | 4882 | 17 | 0.073185 | biological_process |
| 20 | pollen exine formation | GO:0010584 | 19 | 2 | 0.073185 | biological_process |
| 21 | oxidoreductase activity | GO:0016491 | 3755 | 14 | 0.111643 | molecular_function |
| 22 | coenzyme binding | GO:0050662 | 118 | 3 | 0.111643 | molecular_function |
| 23 | fatty-acyl-CoA reductase (alcohol-forming) activity | GO:0080019 | 26 | 2 | 0.115218 | molecular_function |
| 24 | hydrogen-exporting ATPase activity, phosphorylative mechanism | GO:0008553 | 30 | 2 | 0.144864 | molecular_function |
| 25 | substrate-specific transmembrane transporter activity | GO:0022891 | 169 | 3 | 0.258078 | molecular_function |
| 26 | ATP biosynthetic process | GO:0006754 | 42 | 2 | 0.258078 | biological_process |
| 27 | dihydrokaempferol 4-reductase activity | GO:0045552 | 1 | 1 | 0.40856 | molecular_function |
| 28 | hydrolase activity | GO:0016787 | 5182 | 16 | 0.415375 | molecular_function |
| 29 | transmembrane transport | GO:0055085 | 2027 | 8 | 0.894148 | biological_process |
| 30 | alkane 1-monooxygenase activity | GO:0018685 | 4 | 1 | 0.92382 | molecular_function |
